# Synthesis of 3-((4-Hydroxyphenyl)amino)propanoic Acid Derivatives as Promising Scaffolds for the Development of Antimicrobial Candidates Targeting Multidrug-Resistant Bacterial and Fungal Pathogens

**DOI:** 10.3390/antibiotics13020193

**Published:** 2024-02-17

**Authors:** Povilas Kavaliauskas, Birutė Grybaitė, Birutė Sapijanskaitė-Banevič, Rita Vaickelionienė, Vidmantas Petraitis, Rūta Petraitienė, Ethan Naing, Andrew Garcia, Ramunė Grigalevičiūtė, Vytautas Mickevičius

**Affiliations:** 1 Department of Organic Chemistry, Kaunas University of Technology, Radvilenu rd. 19, LT-50254 Kaunas, Lithuania; birute.grybaite@ktu.lt (B.G.); birute.sapijanskaite@ktu.lt (B.S.-B.); rita.vaickelioniene@ktu.lt (R.V.); vytautas.mickevicius@ktu.lt (V.M.); 2 Biological Research Center, Lithuanian University of Health Sciences, Tilzes Street 18, LT-47181 Kaunas, Lithuania; vip2007@med.cornell.edu (V.P.); ramune.grigaleviciute@lsmuni.lt (R.G.); 3 Joan and Sanford I. Weill Department of Medicine, Weill Cornell University, 1300 York Avenue, New York, NY 10065, USA; rop2016@med.cornell.edu (R.P.); etn2001@med.cornell.edu (E.N.); abg2014@med.cornell.edu (A.G.); 4 Department of Microbiology and Immunology, University of Maryland School of Medicine, 655 W. Baltimore Street, Baltimore, MD 21201, USA; 5 Institute of Infectious Diseases and Pathogenic Microbiology, Birstono Street 38A, LT-59116 Prienai, Lithuania; 6 Department of Animal Nutrition, Lithuanian University of Health Sciences, Tilzes Street 18, LT-47181 Kaunas, Lithuania

**Keywords:** ESKAPE group pathogens, amino acid derivatives, 4-hydroxyphenyl moiety

## Abstract

Infections caused by multidrug-resistant bacterial and fungal pathogens represent a significant global health concern, contributing to increased morbidity and mortality rates. Therefore, it is crucial to develop novel compounds targeting drug-resistant microbial strains. Herein, we report the synthesis of amino acid derivatives bearing an incorporated 4-hydroxyphenyl moiety with various substitutions. The resultant novel 3-((4-hydroxyphenyl)amino)propanoic acid derivatives **2**–**37** exhibited structure-dependent antimicrobial activity against both ESKAPE group bacteria and drug-resistant *Candida* species. Furthermore, these derivatives demonstrated substantial activity against *Candida auris*, with minimum inhibitory concentrations ranging from 0.5 to 64 µg/mL. Hydrazones **14**–**16**, containing heterocyclic substituents, showed the most potent and broad-spectrum antimicrobial activity. This activity extended to methicillin-resistant *Staphylococcus aureus* (MRSA) with MIC values ranging from 1 to 8 µg/mL, vancomycin-resistant *Enterococcus faecalis* (0.5–2 µg/mL), Gram-negative pathogens (MIC 8–64 µg/mL), and drug-resistant *Candida* species (MIC 8–64 µg/mL), including *Candida auris*. Collectively, these findings underscore the potential utility of the novel 3-((4-hydroxyphenyl)amino)propanoic acid scaffold for further development as a foundational platform for novel antimicrobial agents targeting emerging and drug-resistant bacterial and fungal pathogens.

## 1. Introduction

Antimicrobial resistance remains one of the biggest public health threats worldwide. The emergence of multidrug-resistant bacterial pathogens remains a growing concern, resulting in an increase in morbidity and mortality [1,2]. The World Health Organization (WHO) identified priority pathogens that are able to evade or escape conventional antimicrobial therapy due to their acquired resistance mechanisms and high virulence or pathogenicity [3,4,5]. These pathogens include *Enterococcus faecium*, *Staphylococcus aureus*, *Klebsiella pneumoniae*, *Acinetobacter baumannii*, *Pseudomonas aeruginosa*, and *Enterobacter* species, resulting in the ESKAPE abbreviation [4,5]. Furthermore, the rapid spread of azoles-resistant fungi such as *Candida auris* worsens treatment outcomes, especially in immunocompromised and elderly individuals [6,7]. Therefore, it is crucial to develop novel candidates targeting non-conventional microbial targets. 

Various unnatural amino acid-derived small molecules have been previously explored as biologically active compounds with antimicrobial properties [8,9,10,11]. Given the essential role of amino acids as building blocks in various biological processes, including cellular signaling, protein synthesis, and metabolism, compounds derived from amino acids present a compelling avenue for investigation as non-conventional antimicrobials in both prokaryotic and eukaryotic organisms. Consequently, synthetic approaches aimed at generating diverse amino acid-based derivatives have the potential to broaden the array of pre-clinical antimicrobial candidates, warranting further exploration and evaluation [9,10,11].

Earlier investigations have revealed that the predominant molecular targets susceptible to inhibition by various amino acid derivatives in pathogenic microorganisms are intricately involved in the synthesis of peptidoglycan or other crucial components of the cell wall [12,13]. These enzymes, including those in the MurA-F pathway, play a crucial role in catalyzing the oligopeptidization of *N*-acetylmuramic acid, ultimately leading to the formation of peptidoglycan [14,15]. These processes necessitate the involvement of non-proteogenic and D-amino acids, rendering them particularly attractive and selective targets for antimicrobial strategies. Moreover, the high synthetic versatility inherent in the amino acid scaffold, along with the capability to integrate various substituents such as small organic groups and heterocyclic and aromatic moieties, holds the promise of generating novel derivatives [10,11,12,13]. These derivatives have the potential to concurrently target multiple critical pathways or essential components in microbial cells, further advancing the development of antimicrobial agents capable of overcoming pre-existing multidrug-resistance phenotypes in pathogens.

Numerous compounds containing the phenolic (4-hydroxyphenyl) moiety are widely recognized for their potent biological activities [16,17,18]. Among them are various FDA-approved and investigational pharmaceuticals known for their anticancer, antimicrobial, anti-inflammatory, and antioxidant properties [16,17,18]. The hydroxyl group of phenols demonstrates remarkable versatility, participating in diverse chemical reactions such as oxidation, hydrogen bond formation, and nucleophilic substitutions [19,20,21]. This inherent property allows for interactions with a diverse array of biological targets. Consequently, the strategic integration of the phenol moiety into amino acid derivatives holds significant promise for the development of highly bioactive compounds, thereby expanding the spectrum of activity against a variety of pathogens.

Continuing our studies in the development of novel candidates targeting multidrug-resistant microbial pathogens, we have synthesized a library of amino acid derivatives featuring an incorporated 4-hydroxyphenyl moiety and diverse substituents. The resulting derivatives, identified as 3-((4-hydroxyphenyl)amino)propanoic acid derivatives, were systematically screened against prevalent ESKAPE group pathogens and drug-resistant fungal pathogens, such as *Candida auris*, characterized by genetically defined resistance profiles.

## 2. Results

**Synthesis of 3-((4-hydroxyphenyl)amino)propanoic acid derivatives 2** **–37**

**Scheme 1:**
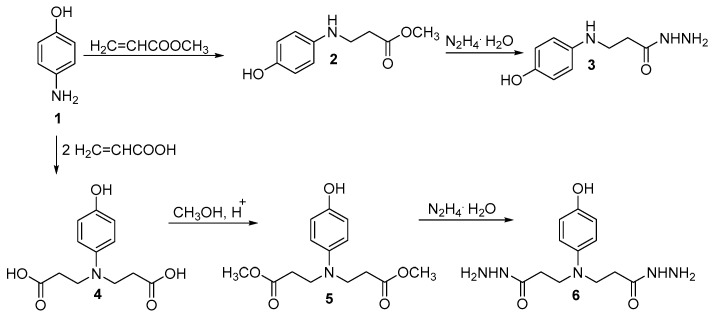
Synthesis of hydrazides **3** and **6**.

In the first stage of this work, by using a well-known methodology described in 1982, the initial compounds **2**–**4** were prepared [22]. According to the methodology, the reaction of 4-aminophenol (**1**) with methyl acrylate in 2-propanol or acrylic acid in water at reflux afforded intermediates *N*-(4-hydroxyphenyl)-*β*-alanine methyl ester (**2**) or 3,3′-((4-hydroxyphenyl)azanediyl)di(propanoic)acid (**4**) (Scheme 1). In continuation of our interest in the chemistry of *N*-substituted *β*-amino acids, *N*-(4-hydroxyphenyl)-*β*-alanine hydrazide (**3**) was synthesized. Dimethyl ester **5** was synthesized through esterification of 3,3′-((4-hydroxyphenyl)azanediyl)di(propanoic)acid (**4**) with an excess of methanol in the presence of a catalytic amount of sulfuric acid.

Subsequently, dihydrazide **6** was obtained through hydrazinolysis of dimethyl ester **5** in propan-2-ol under reflux (Scheme 1). Comparing the ^1^H NMR spectrum of dihydrazide **6** with the ^1^H NMR spectrum of diester **5**, the characteristic signal of the ester groups was not observed at 3.54 ppm. A broad singlet integrated for four protons (4.17 ppm) and two singlets (8.61 and 8.98 ppm), each integrated for one proton, has been attributed to the NH_2_ and NH groups of two hydrazide fragments in the ^1^H NMR spectrum of compound **6**.

**Scheme 2:**
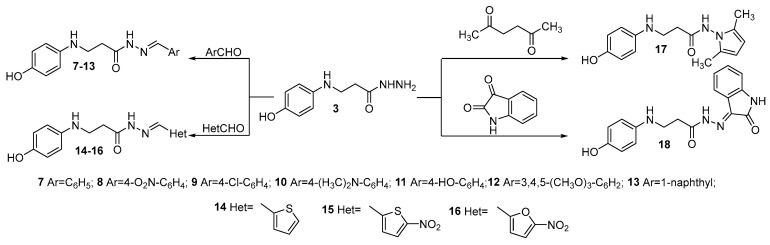
Synthesis of compounds **7**–**18**.

Hydrazones **7**–**13** were synthesized in good yields (58–94%) through the reaction of *N*-(4-hydroxyphenyl)-*β*-alanine hydrazide (**3**) with aromatic aldehydes in methanol at reflux temperature (Scheme 2). Hydrazones **14**–**17** were also obtained analogously, only after using heterocyclic aldehydes instead of aromatic aldehydes. The structures of hydrazones **7**–**16** have been established mainly on the basis of ^1^H and ^13^C NMR spectra (Figures S1–S65). Theoretically, such compounds possessing amide and azomethine groups can exist as inseparable mixtures of four isomers. The amide group determined a splitting of resonances in the ^1^H and ^13^C NMR spectra due to the restricted rotation around the amide bond. The data of the ^1^H and ^13^C NMR spectra led us to conclude that geometrical isomers of the azomethine group were not observed, and in the DMSO-*d_6_* solutions, hydrazones **7**–**16** existed as mixtures of *E*/*Z* isomers, where the *Z* isomer predominated due to the hindered rotation around the CO-NH bond.

Dimethylpyrrole derivative **17** was prepared by refluxing a mixture of the corresponding carbohydrazide **3**, 2,5-hexanedione, 2-propanol, and a catalytic amount of glacial acetic acid (Scheme 2), whereas with isatin, hydrazide **3** formed a hydrazone-type compound 3-((4-hydroxyphenyl)amino)-*N*′-(2-oxoindolin-3-ylidene)propanehydrazide (**18**). 

**Scheme 3:**
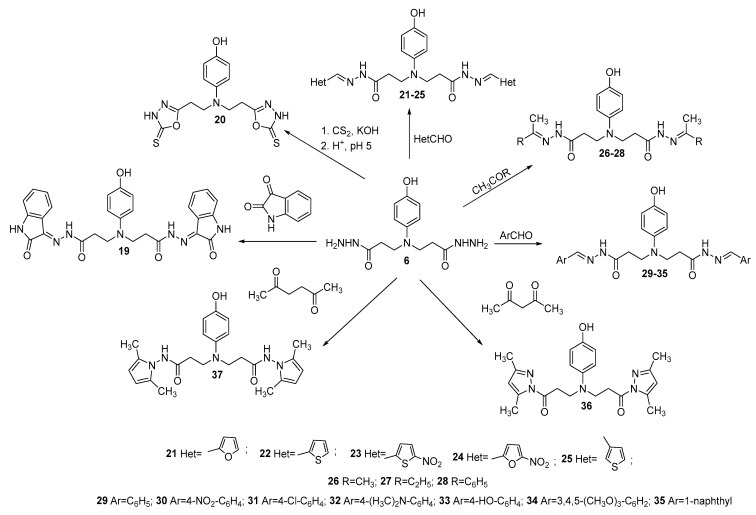
Synthesis of compounds **19**–**37**.

In the next stage of this work, condensation reactions of dihydrazide **6** with various carbonyl compounds were performed, during which a series of hydrazones **19** and **21**–**35**, as well as oxadiazole, dimethylpyrazole, and dimethylpyrrole heterocyclic compounds **20**, **36,** and **37,** were synthesized. Condensation of dihydrazide **6** with aromatic aldehydes and ketones gave the corresponding hydrazones **21**–**35** (Scheme 3) among the compounds due to the mode of substitution in the azomethine fragment. The presence of particular substitution patterns in the benzene ring as well as a mono substituent of the azomethine fragment caused the formation of geometrical isomers. Taking into account the two isomerism centers existing in each side chain, 10 isomers of compounds **21**–**35** can be formed. NMR did not provide conclusive information about the separate conformations but gave a time-averaged spectral view of the structures present in the solution. The restricted rotation around the CONH led to the formation in an isomeric mixture of hydrazones, where the *Z* isomer predominates. The obtained hydrazones **7**–**16** and **21**–**35** show double sets of resonances for the N=CH and CONH fragment protons with an intensity ratio of 0.35:0.65 (^1^H NMR). The structures of similar compounds are described in our previous works [23,24,25,26]. The synthesis of the compounds and their spectroscopic data are presented in the Supplementary Materials.

**Antimicrobial activity of 3-((4-hydroxyphenyl)amino)propanoic acid derivatives 2–37 against multidrug-resistant pathogens**

Following the synthesis and characterization of novel 3-((4-hydroxyphenyl)amino)propanoic acid derivatives **2**–**37**, our primary objective was to investigate their antimicrobial activity against a variety of pathogenic bacterial and fungal organisms. The synthesized compounds were screened against diverse libraries of clinically relevant bacterial and fungal pathogens, harboring various multidrug-resistance phenotypes. The selection of isolates was undertaken to represent pathogens identified by the World Health Organization as high-priority ESKAPE group organisms, given their escalating levels of antimicrobial resistance in the clinical field. 

The intermediate starting compound **2** showed weak antimicrobial activity against *S. aureus* and *E. faecalis*, while **4** demonstrated no antibacterial activity against both Gram-positive and Gram-negative bacterial strains (Table 1). Compounds **2** and **4** exhibited no antifungal activity against drug-resistant *Candida* species (Table 2). Subsequent chemical modifications of compound **2** yielded *N*-(4-hydroxyphenyl)-*β*-alanine hydrazide **3**, which did not display antibacterial activity across all tested bacterial strains (MIC > 64 µg/mL). Esterification of compound **4** resulted in dimethyl ether **5**, devoid of both antifungal and antibacterial properties. Further transformation of dimethyl ether **5** into dihydrazide **6** resulted in a compound exhibiting antimicrobial activity against Gram-negative pathogens. Specifically, the derivative showed activity against carbapenemase-producing *E. coli* AR-0001 and *K. pneumoniae* AR-003, with MIC values of 64 µg/mL. Moreover, compound **6** demonstrated activity against multidrug-resistant *P. aeruginosa* AR-1114 and *A. baumannii* AR-0273, with MIC values of 32 µg/mL, respectively (Table 1). 

Hydrazone **7**, bearing a phenyl substituent, demonstrated antimicrobial activity against the methicillin-resistant *S. aureus* TCH-1516 strain (MIC of 32 µg/mL) but displayed no activity against vancomycin-resistant *E. faecalis* AR-0671 (MIC > 64 µg/mL). Additionally, moderate activity was observed against *E. coli* AR-001 and *K. pneumoniae* AR-0003 (MIC 64 µg/mL), while no activity was detected against carbapenemases-producing *P. aeruginosa* AR-1114 or *A. baumannii* AR-0273. The introduction of a 4-NO_2_ substitution on the phenyl ring (compound **8**) significantly enhanced activity against *S. aureus* TCH-1516 and *E. faecalis* (MIC of 16 and 8 µg/mL, respectively) as well as *E. coli* (MIC of 16 µg/mL), albeit with reduced activity against *K. pneumoniae* AR-0003, *P. aeruginosa* AR-1114, and *A. baumannii* AR-0273 (MIC > 64 µg/mL) (Table 1).

Furthermore, the introduction of a 4-Cl substituent in the phenyl ring (compound **9**) augmented activity directed toward Gram-positive bacteria while concurrently diminishing activity against Gram-negative pathogens, with the exception of *A. baumannii*. Notably, the introduction of a dimethylamino substituent in the phenyl ring (compound **10**) resulted in a complete loss of antimicrobial activity against all tested bacterial strains. Moreover, the introduction of a 4-OH group (compound **11**) or a 3,4,5-(CH_3_O) moiety (compound **12**) failed to restore antimicrobial activity. Conversely, the introduction of a 1-naphthyl substituent (compound **13**) resulted in the restoration of antimicrobial activity against *S. aureus* TCH-1516 and *E. faecalis* AR-0671 (MIC of 16 µg/mL), as well as *E. coli* AR-0001 (MIC of 32 µg/mL), *K. pneumoniae* AR-0003 (MIC of 64 µg/mL), and *A. baumannii* AR-0273 (MIC of 64 µg/mL).

Hydrazones **14**–**17**, bearing various heterocyclic substituents, exhibited notably enhanced antimicrobial efficacy against bacterial and fungal pathogens. Specifically, compound **14**, bearing a 2-thiophene substituent, demonstrated a broad spectrum of antibacterial and antifungal activity against all tested bacterial strains (MIC of 8–64 µg/mL). Notably, hydrazone **14** exhibited substantial activity against *S. aureus* TCH-1516 and *E. faecalis* AR-0671 (8 µg/mL), with comparatively weaker activity observed against *E. coli* AR-0001, *K. pneumoniae* AR-0003, *P. aeruginosa* AR-1114, and *A. baumannii* AR-0273 (64 µg/mL). The introduction of a nitro group into the thiophene backbone remarkably enhanced the antimicrobial activity of compound **15**. This derivative displayed potent activity against *S. aureus* (MIC of 1 µg/mL) and *E. faecalis* AR-0671 (MIC < 0.5 µg/mL), as well as *E. coli* AR-0001 (MIC of 8 µg/mL), *A. baumannii* AR-0276 (MIC 16 µg/mL), and *K. pneumoniae* AR-003 (MIC of 32 µg/mL). Interestingly, the incorporation of the nitro group resulted in a reduction in the antifungal spectrum of compound **15**, confining its activity solely to *C. albicans*. These findings underscore the structure–activity relationship of the hydrazone derivatives and highlight the potential of compound **15** as a potent antimicrobial agent with selectivity toward specific bacterial and fungal strains. The replacement of nitrothiophene substituent with nitrofurane (compound **16**) preserved Gram-positive bacteria-targeted activity, while decreasing the antimicrobial spectrum against Gram-negative pathogens. On the other hand, the introduction of nitrofurane substitution enhanced the antifungal activity against *C. albicans* (MIC of 8 µg/mL), *C. parapsilosis* (MIC of 16 µg/mL), and *C. auris* strains (MIC of 32 µg/mL). Finally, dimethylpyrole containing derivative **17** showed no antibacterial activity (>64 µg/mL) against the majority of strains, although favorable activity was observed against *A. baumannii* (MIC of 16 µg/mL). Furthermore, strong antifungal activity was observed against drug-resistant *Candida* species (MIC of 8–16 µg/mL). Compounds with an indolinone moiety (compounds **18** and **19**) had fully diminished antifungal and antibacterial activity. 

Upon elucidating the structure–activity relationships of hydrazide derivatives, our investigation progressed toward the examination of symmetric dihydrazide-based transformations, postulating potential enhancements in antimicrobial properties. Specifically, dihydrazide **6** served as the starting point, undergoing a series of chemical transformations wherein it was condensed with diverse carbonyl compounds, yielding hydrazone, oxadiazole, dimethylpyrazole, and dimethylpyrrole heterocyclic derivatives, each harboring distinct structural substitutions. Of note, oxadiazole derivative **20** showed promising antimicrobial activity against *S. aureus* TCH-1516 and *E. faecalis* AR-0671, with a minimum inhibitory concentration (MIC) of 32 µg/mL. These compounds exhibited a lack of antimicrobial activity against Gram-negative pathogens and fungal strains. 

We then evaluated whether the incorporation of heterocyclic substitutions would have an impact on the antimicrobial activity of synthesized compounds. Compound **21**, bearing a 2-furyl ring, displayed enhanced antimicrobial efficacy against tested *S. aureus* and *E. faecalis* strains, with an MIC of 16 µg/mL. Additionally, compound **21** demonstrated activity against *E. coli* and *K. pneumoniae*, while showing no activity on *P. aeruginosa* (MIC >64 µg/mL). The replacement of furane with thiophene in compound **22** expanded the antimicrobial spectrum, particularly increasing the activity against tested *P. aeruginosa*, with an MIC of 64 µg/mL. Intriguingly, the introduction of a nitro group into the thiophene substituent, as observed in compound **23**, resulted in a complete loss of antimicrobial activity against all tested bacterial and fungal strains. In contrast, the incorporation of a nitro substituent into furane, as observed in compound **24**, remarkably restored antimicrobial activity against both Gram-positive and Gram-negative pathogens. Finally, the addition of a thien-3-yl moiety in compound **25** resulted in modest activity against Gram-positive pathogens (MIC of 32 and 64 µg/mL) and negligible efficacy against Gram-negative pathogens or fungal strains. 

Following the characterization of the impact of heterocycle substitution on the antimicrobial activity of dihydrazide derivatives, our investigation extended to the examination of how the inclusion of methyl or ethyl substitutions influences biological activity. Compound **26**, bearing a CH_3_ substituent, and compound **27**, containing a C_2_H_5_ substituent, demonstrated no discernible antimicrobial activity against all tested strains (MIC >64 µg/mL). Conversely, the incorporation of a phenyl substituent in compound **28** resulted in weak antimicrobial activity against *E. coli* (MIC of 32 µg/mL), *K. pneumoniae* (MIC of 64 µg/mL), and *A. baumannii* (MIC of 64 µg/mL). 

Further exploration of the influence of various aromatic substituents on the antimicrobial activity of 3-((4-hydroxyphenyl)amino)propanoic acid derivatives revealed that the inclusion of a phenyl substituent in compound **29** exhibited antimicrobial activity against *S. aureus* (MIC of 16 µg/mL) but not *E. faecalis* (MIC >64 µg/mL). The incorporation of a 4-NO_2_ substitution in the phenyl ring in compound **30** led to enhanced activity against *S. aureus* and *E. faecalis* (MIC of 16 µg/mL), as well as *E. coli* (MIC of 32 µg/mL) and *K. pneumoniae* (MIC of 64 µg/mL). The substitution of a nitro group with 4-Cl resulted in compound **31** demonstrating increased activity against Gram-negative pathogens (MIC of 32–64 µg/mL), including efficacy against *P. aeruginosa* (MIC of 64 µg/mL) and *A. baumannii (*MIC of 32 µg/mL).

Notably, the incorporation of a basic substitution such as dimethylamino (compound **32**) nullified activity against all tested organisms, except for *A. baumannii* (MIC of 16 µg/mL). Additionally, the introduction of a 4-OH substituent in compound **33** yielded substantial antimicrobial activity against both Gram-positive (MIC of 8–16 µg/mL) and Gram-negative pathogens (MIC of 16–64 µg/mL), including *P. aeruginosa* (MIC of 16 µg/mL) and *A. baumannii* (MIC of 16 µg/mL). The dimethylpyrazole derivative **36** demonstrated activity primarily against *S. aureus* and *E. faecalis* (MIC of 32 and 64 µg/mL, respectively), while the inclusion of dimethylpyrrole in compound **37** resulted in slightly expanded activity against *E. coli* (MIC of 16 µg/mL).

Collectively, these results demonstrate that 3-((4-hydroxyphenyl)amino)propanoic acid derivatives exhibit a structure-dependent and potent antimicrobial activity against ESKAPE group pathogens and drug-resistant fungi. 

**3-((4-hydroxyphenyl)amino)propanoic acid scaffold demonstrates favorable pharmacological ADME properties**

After characterizing the antimicrobial activity of 3-((4-hydroxyphenyl)amino)propanoic acid derivatives and establishing SAR relations, we further selected the most promising antibacterial and antifungal compounds and subjected them to in silico absorption, distribution, metabolism, and excretion (ADME) characterization to better understand the pharmacological properties of the 3-((4-hydroxyphenyl)amino)propanoic acid scaffold. 

**Figure 1:**
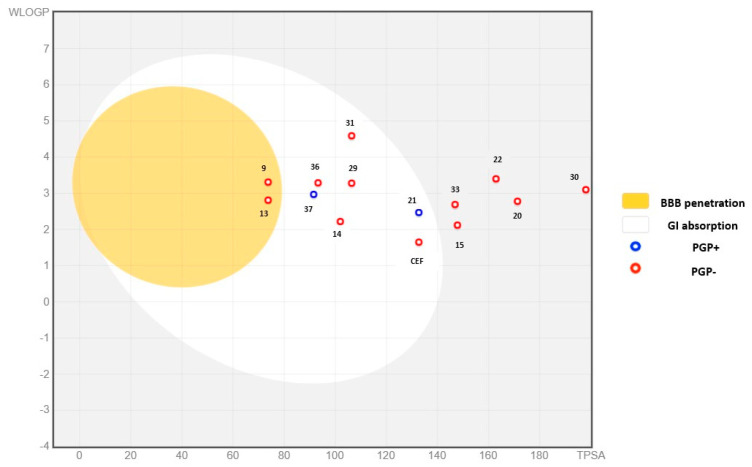
The boiled egg model illustrates the ADME properties of selected antibacterial derivatives of 3-((4-hydroxyphenyl)amino)propanoic acid. Blue dots indicate compounds predicted to be P-gp substrates, potentially undergoing active efflux from the central nervous system (CNS). Conversely, red dots indicate compounds predicted not to be P-gp substrates and not expected to undergo active efflux from the CNS.

**Figure 2:**
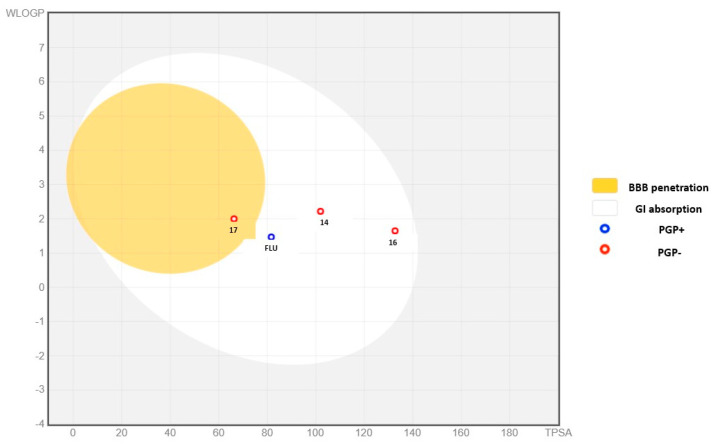
The boiled egg model illustrates the ADME properties of selected antifungal derivatives of 3-((4-hydroxyphenyl)amino)propanoic acid. Blue dots indicate compounds predicted to be P--gp substrates, potentially undergoing active efflux from the central nervous system (CNS). Conversely, red dots indicate compounds predicted not to be P-gp substrates and not expected to undergo active efflux from the CNS.

The compounds selected for in silico ADME prediction were chosen to represent a broad-spectrum antibacterial activity against multiple MDR strains. Among such compounds, **9**, **13**–**16**, **20**–**22**, **24, 29**, **30**, **31**, **33**, and **36**–**37** were selected for in silico ADME analysis and compared to the reference antimicrobial drug, cefazolin (CEF) (Figure 1). For compounds with antifungal activity, 3-((4-hydroxyphenyl)amino)propanoic acid derivatives **14**, **16,** and **17** were selected and compared with fluconazole (FLU) (Figure 2). 

The physicochemical properties of selected antibacterial 3-((4-hydroxyphenyl)amino)propanoic acid derivatives were assessed to gain insights into their molecular characteristics. The molecular weight (MW) of the compounds ranged from 289.35 to 547.52 g/mol, with compound **30** exhibiting the highest MW. The number of heavy atoms varied between 20 and 40, with compound **14** having the lowest and compound **30** having the highest number (Table 3). The fraction of sp^3^-hybridized carbon atoms (Csp^3^) ranged from 0.1 to 0.43, reflecting differences in molecular rigidity. Compounds **14** and **15** showed a higher fraction of sp^3^ hybridization, indicative of increased aliphatic character. The number of rotatable bonds ranged from 7 to 15, with compound **14** having the lowest and compounds **30** and **31** having the highest numbers. Hydrogen bond acceptors and donors varied across compounds, with compound **24** having the highest number of acceptors (11) and compound **37** having the highest number of donors (3) (Table 3). 

Among the analyzed antibacterial compounds, eight (**9**, **13**, **14**, **29**, **31**, **36**, and **37**) were predicted to possess drug-like properties. Specifically, compounds **9** and **13** were predicted to exhibit the ability to passively permeate the blood–brain barrier (BBB) (Figure 1). These compounds displayed favorable physicochemical properties, including a molecular mass ranging from 317 to 333 g/mol, a suitable number of aromatic and heavy atoms, as well as an optimal count of rotatable bonds, and hydrogen acceptors and donors (Table 3).

Compounds **14**, **29**, **31**, **36**, and **37** demonstrated favorable gastrointestinal absorption (GI absorption) parameters (Figure 1, Table 4). Interestingly, only compound **37** was predicted to be a substrate of P-gp glycoprotein and to be actively exported from the CNS. 

The in silico ADME prediction results for selected antibacterial compounds and their interaction with the human cytochrome P450 (CYP) system reveal distinct profiles across various CYP isoforms. Compounds **9**, **13**, **14**, **15**, **21**, **22**, **24**, **29**, **30**, **31**, **36**, and **37** were investigated for their inhibitory effects on CYP1A2, CYP2C19, CYP2C9, CYP2D6, and CYP3A4. Compounds **9** and **13** exhibited significant inhibitory activity across all tested CYP isoforms, suggesting potential implications for drug metabolism and interactions. Compounds **14**, **15**, **21**, **22**, **24**, **29**, **30**, **31**, **36**, and **37** displayed varying degrees of inhibition, highlighting the importance of considering multiple CYP isoforms in drug development. Compound **20** selectively inhibited CYP3A4, indicating specificity for this isoform. Compound **16** and the reference antibiotic CEF showed negligible inhibitory effects on the tested CYP isoforms. These findings contribute valuable insights into the potential pharmacokinetic interactions and metabolic fate of the investigated antibacterial compounds, aiding in the rational design of safer and more effective therapeutic agents.

Among the selected compounds with antifungal activity against MDR fungal pathogens, the gastrointestinal (GI) absorption, blood–brain barrier (BBB) permeability, and ability to be a P-glycoprotein (P-gp) substrate was evaluated through in silico modeling. Compounds **14** and **16** exhibit high GI absorption, suggesting favorable oral absorption characteristics. However, both compounds are not BBB permeant and are not substrates for P-gp efflux transporters. Compound **17**, while also displaying high GI absorption, is BBB permeant but is not a P-gp substrate. Fluconazole (FLU) demonstrates high GI absorption, is not BBB permeant, but is recognized as a P-gp substrate, indicating a potential for active efflux at the BBB (Figure 2, Table 5). The assessed antifungal compounds demonstrate distinct inhibitory profiles against various CYP 450 isoforms. Compound **14** exhibits inhibitory activity against CYP1A2 but not against other tested isoforms (CYP2C19, CYP2C9, CYP2D6, and CYP3A4). In contrast, compound **16** does not show inhibitory effects on any of the assessed CYP isoforms. Compound **17** is an inhibitor of CYP1A2 and CYP2D6, suggesting potential interactions with these enzymes. Fluconazole acts as a CYP2C19 inhibitor while remaining inactive against the other tested isoforms (Table 5).

The molecular weight of the compounds ranges from 273.33 to 318.28 g/mol. Notably, compounds **14**, **16**, and fluconazole (FLU) exhibit varying heavy atom counts, ranging from 20 to 23. Aromatic heavy atom numbers range from 11 to 16, with FLU possessing the highest count. The fraction of sp^3^-hybridized carbon atoms (Csp3) varies among the compounds, indicating differences in molecular rigidity. Compounds 14, 16, and FLU display a fraction of 0.14, suggesting a comparable level of aliphatic character. The number of rotatable bonds ranges from 5 to 8, with compound **17** having the lowest and compound **16** having the highest (Table 6).

This comprehensive molecular modeling provides valuable information for understanding the structural diversity and potential pharmacokinetic properties of the investigated antibacterial and antifungal 3-((4-hydroxyphenyl)amino)propanoic acid-based compounds.

## 3. Discussion

The rising prevalence of antimicrobial resistance among clinically significant bacterial and fungal pathogens underscores the need for urgent exploration of novel candidates and pharmacophores for subsequent pre-clinical evaluation. This study delineates a synthetic methodology designed to generate a diverse array of xenogeneic amino acid derivatives based on 3-((4-hydroxyphenyl)amino)propanoic acid, incorporating various aromatic and heterocyclic substitutions. Through antimicrobial activity characterization using genetically defined ESKAPE group pathogens harboring emerging resistance mechanisms, we demonstrated that the 3-((4-hydroxyphenyl)amino)propanoic acid-based pharmacophore exhibits promising activity against bacterial and fungal pathogens. 

Various proteogenic and non-proteogenic amino acids play a crucial role in signaling, homeostasis, and protein synthesis in both eukaryotic and prokaryotic organisms, rendering the amino acid axis an attractive pathway for antimicrobial strategies [27,28,29]. The utilization of amino acid derivatives as antimicrobials has been previously suggested by several researchers. Synthetic pathways and compounds have also been explored through diverse chemical approaches and model systems [30,31,32]. Lopez et al. conducted a comprehensive investigation into the synthesis and antimicrobial activity of naphthoquinone moieties containing amino acid derivatives [30]. Their study revealed promising antimicrobial efficacy against wild-type bacterial strains. Meanwhile, the research by Chui et al. delved into the antimicrobial properties of hydroxypropyltrimethyl ammonium chitosan derivatives bearing amino acid Schiff bases. Notably, this study successfully demonstrated robust antimicrobial activity, primarily targeting *S. aureus* and fungal strains [33]. Despite the previously reported antimicrobial activity of various amino acid-based derivatives, it is noteworthy that a substantial portion of these studies predominantly involve wild-type and pan-susceptible bacterial isolates. This approach often overlooks the incorporation of multidrug-resistance mechanisms, thereby providing a less representative portrayal of the current resistance phenotypes circulating in clinical settings. Furthermore, drug-resistant bacterial and fungal pathogens frequently exhibit a spectrum of resistance mechanisms, capable of directly or indirectly modifying diverse chemical compounds or actively exporting them through the overexpression of efflux pumps.

In this study, we explored 3-((4-hydroxyphenyl)amino)propanoic acid derivatives for their antimicrobial efficacy against the prevalent ESKAPE pathogens (*Enterococcus* spp., *Staphylococcus aureus*, *Klebsiella pneumoniae*, *Acinetobacter baumannii*, and *Pseudomonas aeruginosa*) [34,35]. Furthermore, by performing in silico ADME characterization using selected antibacterial and antifungal derivatives, we successfully demonstrated the pharmacological applicability of 3-((4-hydroxyphenyl)amino)propanoic acid derivatives as promising drug-like hits suitable for future hit-to-lead optimization. The 4-hydroxyphenyl substituent serves as a well-established pharmacophore frequently encountered in numerous FDA-approved pharmaceuticals. This moiety plays a pivotal role in augmenting the hydrophilicity of compounds, thereby potentially improving their solubility. Additionally, the 4-hydroxyphenyl radical contributes to enhanced reactivity by serving as a site for hydrogen bond formation. Given these characteristics, we hypothesized that substituted amino acid derivatives based on 4-hydroxyphenyl and propionic acid, incorporating diverse substituents, could yield potent antimicrobial activity against a spectrum of microbial pathogens.

We demonstrated that hydrazones **7**–**13** exhibited selective antimicrobial activity against methicillin-resistant *S. aureus* and vancomycin-resistant *E. faecalis* strains. Interestingly, no activity was observed against MDR Gram-negative bacterial isolates or drug-resistant strains, suggesting the Gram-positive bacteria-directed activity of hydrazones **7**–**13**. Interestingly, the incorporation of heterocyclic substituents in hydrazone-based 3-((4-hydroxyphenyl)amino)propanoic acid derivatives (**14**–**17**) greatly enhanced the antimicrobial activity. These compounds showed activity against both Gram-positive and Gram-negative pathogens, as well as challenging fungal pathogens, including azole-resistant *Candida auris.* Among these compounds, hydrazone derivatives bearing nitro thiophene (**15**) and nitro furane (**16**) showed the most promising antibacterial activity, while compounds **14** and **17** bearing thiophene and dimethylpyrrole demonstrated promising antifungal activity against all tested isolates. Interestingly, the generation of dihydrazide derivatives with the identical substitutions decreased the antimicrobial activity.

## 4. Materials and Methods

**Chemical synthesis**

**General procedures**

Reagents and solvents were purchased from Sigma-Aldrich (St. Louis, MO, USA) and used without further purification. The reaction course and purity of the synthesized compounds were monitored by TLC using aluminum plates pre-coated with Silica gel with F_254_ nm (Merck KGaA, Darmstadt, Germany). Melting points were determined with a *B-540* melting point analyzer (Büchi Corporation, New Castle, DE, USA) and were uncorrected. IR spectra (ν, cm^−1^) were recorded on a Perkin–Elmer Spectrum BX FT–IR spectrometer (Perkin–Elmer Inc., Waltham, MA, USA) using KBr pellets. NMR spectra were recorded on a Brucker Avance III (400, 101 MHz) spectrometer (Bruker BioSpin AG, Fällanden, Switzerland). Chemical shifts were reported in (*δ*) ppm relative to tetramethylsilane (TMS), with the residual solvent as internal reference ([D*_6_*]DMSO, *δ* = 2.50 ppm for ^1^H and δ = 39.5 ppm for ^13^C). Data are reported as follows: chemical shift, multiplicity, coupling constant [Hz], integration, and assignment. Elemental analyses (C, H, and N) were conducted using the Elemental Analyzer CE-440, and their results were found to be in good agreement (±0.3%) with the calculated values. Mass spectra were measured on a Bruker maXis 4G mass spectrometer.

**Synthetic procedures**

*N-(4-hydroxyphenyl)-β-alanine methyl* ester (**2**)

was resynthesized according to the described procedure [22]. 

*N-(4-hydroxyphenyl)-β-alanine hydrazide* (**3**)

was resynthesized according to the described procedure [22]. 

*((4-Hydroxyphenyl)azanediyl)dipropanoic acid* (**4**)

was resynthesized according to the described procedure [22]. 

*((4-Hydroxyphenyl)azanediyl)dimethyl ester* (**5**)

was resynthesized according to the described procedure [23].

*3,3′-((4-Hydroxyphenyl)azanediyl)di(propanehydrazide)* (**6**)

A mixture of methyl ester **5** (5.26 g, 18.7 mmol), hydrazine hydrate (9.58 g, 191.27 mol), and 2-propanol (30 mL) was heated under reflux for 24 h and cooled. Crystalline product **6** was filtered off, washed with 2-propanol, and dried. White powder was obtained at a yield 5.12 g (97%), m.p. 149–150 °C (from 2-propanol). ^1^H NMR (400 MHz, DMSO-*d_6_*) δ: 2.19 (t, *J* = 7.2 Hz, 4H, 2× CH_2_CO), 3.34 (t, *J* = 7.2 Hz, 4H, 2× NCH_2_), 4.17 (s, 4H, 2× NH_2_), 6.56–6.67 (m, 4H, H_Ar_), 8.61 (s, 1H, OH), and 8.98 (s, 2H, 2× N*H*NH_2_). ^13^C NMR (101 MHz, DMSO-*d_6_*) δ: 31.64, 47.91, 115.29, 115.82, 140.56, 149.13, and 170.27. IR (KBr): ν_max_ (cm^−1^) = 3321 (OH), 3263 (NH), 3199 (NH_2_), and 1611 (C=O). Anal. Calcd. for C_12_H_19_N_5_O_3_, %: C, 51.23; H, 6.81; N, 24.90. Found: C, 50.93; H, 6.50; N, 24.61.

*General procedure for the preparation of hydrazones* **7**–**13**

To a solution of hydrazide **3** (1.5 mmol) in methanol (11 mL), the corresponding aromatic aldehyde was added (1.65 mmol); the mixture was heated at reflux temperature for 2 h, then cooled down, and the formed precipitate was filtered off, washed with methanol and diethyl ether, and recrystallized from 1,4-dioxane.

*N′-benzylidene-3-((4-hydroxyphenyl)amino)propanehydrazide (* **7** *)*

Grayish powder, yield 0.28 g, 67%, m.p. 178–179 °C. ^1^H NMR (400 MHz, DMSO-*d_6_*) δ: 2.45 and 2.87 (2t *J* = 7.1, 7.2 Hz, 2H, CH_2_CO), 3.18–3.30 (m, 2H, NCH_2_), 4.97 (s, 1H, N*H*CH_2_), 6.28–6.50 (m, 2H, H_Ar_), 6.51–6.59 (m, 2H, H_Ar_), 7.28–7.47 (m, 3H, H_Ar_), 7.53–7.71 (m, 2H, H_Ar_), 7.99 and 8.16 (2s, 1H, N=CH), and 8.42 (s, 1H, OH), and 11.32 and 11.38 (2s, 1H, NH). ^13^C NMR (101 MHz, DMSO-*d_6_*) δ: 32.35, 34.39, 113.51, 113.59, 126.66, 126.96, 128.81, 129.71, 134.28, 141.46, 142.75, 145.89, 148.39, 167.45, and 173.18. IR (KBr): ν_max_ (cm^−1^) = 3368 (OH), 3291 (NH), and 1661 (C=O). Anal. Calcd. for C_16_H_17_N_3_O_2_, %: C, 67.83; H, 6.05; N, 14.83. Found, %: C, 67.59; H, 5.94; N, 14.60.

*3-((4-Hydroxyphenyl)amino)-N′-(4-nitrobenzylidene)propanehydrazide (* **8** *)*

Yellowish powder, yield 0.46 g, 94%, m.p. 208–209 °C. ^1^H NMR (400 MHz, DMSO-*d_6_*) δ: 2.50 (one part of the signal of the CH_2_CO overlaps with the DMSO-*d_6_*) and 2.91 (t, *J* = 7.0 Hz, 2H, CH_2_CO), 3.19–3.31 (m, 2H, NCH_2_), 4.97 (s, 1H, N*H*CH_2_), 6.46 (d, *J* = 8.5 Hz, 2H, H_Ar_), 6.56 (d, *J* = 8.4 Hz, 2H, H_Ar_), 7.87 (d, *J* = 8.5 Hz, 2H, H_Ar_), 7.93 (d, *J* = 8.5 Hz, 2H, H_Ar_), 8.03–8.31 (m, 3H, H_Ar_, N=CH), 8.39–8.47 (m, 1H, OH), and 11.62 and 11.69 (2s, 1H, CONH). ^13^C NMR (101 MHz, DMSO-*d_6_*) δ: 32.14, 34.44, 113.55, 113.61, 115.72, 124.03, 127.54, 127.87, 140.40, 140.61, 140.76, 141.37, 141.41, 143.43, 147.57, 147.74, 148.44, 148.49, 167.93, and 173.65. IR (KBr): ν_max_ (cm^−1^) = 3325 (OH), 3276 (NH), and 1674 (C=O). Anal. Calcd. for C_16_H_16_N_4_O_4_, %: C, 58.53; H, 4.91; N, 17.06. Found, %: C, 58.30; H, 4.68; N, 16.92.

*N′-(4-chlorobenzylidene)-3-((4-hydroxyphenyl)amino)propanehydrazide (* **9** *)*

Gray powder, yield 0.24 g, 50%, m.p. 169–170 °C. ^1^H NMR (400 MHz, DMSO-*d_6_*) δ: 2.45 and 2.87 (2t, *J* = 7.0, 7.1 Hz, 2H, CH_2_CO), 3.18–3.29 (m, 2H, NCH_2_), 5.27 (s, 1H, N*H*CH_2_), 6.40–6.52 (m, 2H, H_Ar_), 6.57 (d, *J* = 8.4 Hz, 2H, H_Ar_), 7.38–7.55 (m, 2H, H_Ar_), 7.57–7.79 (m, 2H, H_Ar_), 7.98 and 8.15 (2s, 1H, N=CH), 8.37–8.57 (m, 1H, OH), and 11.38 and 11.45 (2s, 1H, CONH). ^13^C NMR (101 MHz, DMSO-*d_6_*) δ: 32.12, 34.29, 113.86, 115.74, 128.29, 128.60, 128.88, 133.23, 133.33, 134.11, 134.32, 141.01, 141.50, 144.61, 148.70, 167.52, and 173.22. IR (KBr): ν_max_ (cm^−1^) = 3341 (OH), 3269 (NH), and 1670 (C=O). Anal. Calcd. for C_16_H_16_ClN_3_O_2_, %: C, 60.48; H, 5.08; N, 13.22. Found, %: C, 60.20; H, 4.85; N, 13.01.

*N′-(4-(dimethylamino)benzylidene)-3-((4-hydroxyphenyl)amino)propanehydrazide (* **10** *)*

Gray powder, yield 0.36 g, 73%, m.p. 191–192 °C. ^1^H NMR (400 MHz, DMSO-*d_6_*) δ: 2.41 and 2.83 (2t, *J* = 7.0, 7.1 Hz, 2H, CH_2_CO), 2.95 (s, 6H, N(CH_3_)_2_), 3.16–3.28 (m, 2H, NCH_2_), 4.96 (s, 1H, N*H*CH_2_), 6.42–6.51 (m, 2H, H_Ar_), 6.52–6.61 (m, 2H, H_Ar_), 6.72 (d, *J* = 8.4 Hz, 2H, H_Ar_), 7.47 (t, *J* = 8.4 Hz, 2H, H_Ar_), 7.86 and 8.00 (2s, 1H, N=CH), 8.42 (s, 1H, OH), and 11.02 and 11.08 (2s, 1H, CONH). ^13^C NMR (101 MHz, DMSO-*d_6_*) δ: 32.40, 34.38, 40.50, 111.79, 111.83, 113.50, 113.58, 115.71, 121.64, 121.69, 127.92, 128.26, 141.45, 141.50, 143.64, 146.73, 148.36, 148.44, 151.25, 151.39, 166.82, and 172.58. IR (KBr): ν_max_ (cm^−1^) = 3361 (OH), 3236 (NH), and 1652 (C=O). Anal. Calcd. for C_18_H_22_N_4_O_2_, %: C, 66.24; H, 6.79; N, 17.17. Found, %: C, 66.00; H, 6.59; N, 16.98.

*N′-(4-hydroxybenzylidene)-3-((4-hydroxyphenyl)amino)propanehydrazide (* **11** *)*

Gray powder, yield 0.27 g, 60%, m.p. 208–209 °C. ^1^H NMR (400 MHz, DMSO-*d_6_*) δ: 2.41 and 2.83 (2t, *J* = 7.0, 7.1 Hz, 2H, CH_2_CO), 3.15–3.30 (m, 2H, NCH_2_), 4.96 (s, 1H, N*H*CH_2_), 6.42–6.51 (m, 2H, H_Ar_), 6.56 (d, *J* = 8.6 Hz, 2H, H_Ar_), 6.81 (d, *J* = 8.7 Hz, 2H, H_Ar_), 7.48 (t, *J* = 8.6 Hz, 2H, H_Ar_), 7.89 and 8.04 (2s, 1H, N=CH), 8.42 (s, 1H, OH), 9.87 and 9.88 (2s, 1H, N=CC_6_H_4_O*H*), and 11.11 and 11.17 (2s, 1H, CONH). ^13^C NMR (101 MHz, DMSO-*d_6_*) δ: 32.37, 34.37, 113.50, 113.59, 115.65, 115.68, 115.72, 125.31, 128.35, 128.70, 141.43, 141.48, 143.10, 146.23, 148.38, 148.45, 159.07, 159.25, 167.07, and 172.82. IR (KBr): ν_max_ (cm^−1^) = 3377 (OH), 3233 (2× NH), and 1648 (C=O). Anal. Calcd. for C_16_H_17_N_3_O_3_, %: C, 64.20; H, 5.72; N, 14.04. Found, %: C, 64.00; H, 5.50; N, 13.95.

*3-((4-Hydroxyphenyl)amino)-N′-(3,4,5-trimethoxybenzylidene)propanehydrazide (* **12** *)*

Gray powder, yield 0.40 g, 71%, m.p. 110–111 °C. ^1^H NMR (400 MHz, DMSO-*d_6_*) δ: 2.45 and 2.88 (2t, *J* = 7.0, 7.1 Hz, 2H, CH_2_CO), 3.17–3.31 (m, 2H, NCH_2_), 3.69, 3.79, and 3.81 (3s, 9H, 3× OCH_3_), 4.93 (s, 1H, N*H*CH_2_), 6.46 (d, *J* = 8.4 Hz, 2H, H_Ar_), 6.55 (t, *J* = 8.4 Hz, 2H, H_Ar_), 6.95 and 6.98 (2s, 2H, H_Ar_), 7.91 and 8.08 (2s, 1H, N=CH), 8.41 (s, 1H, OH), and 11.34 and 11.36 (2s, 1H, CONH). ^13^C NMR (101 MHz, DMSO-*d_6_*) δ: 32.26, 34.39, 55.92, 55.93, 60.12, 60.13, 103.96, 104.17, 113.57, 113.63, 115.69, 115.73, 129.83, 129.89, 138.93, 139.04, 141.42, 141.56, 142.66, 145.91, 148.42, 148.48, 153.17, 167.43, and 173.19. IR (KBr): ν_max_ (cm^−1^) = 3379 (OH), 3239 (NH), and 1635 (C=O). Anal. Calcd. for C_19_H_23_N_3_O_5_, %: C, 61.12; H, 6.21; N, 11.25. Found, %: C, 60.94; H, 6.01; N, 11.02.

*3-((4-Hydroxyphenyl)amino)-N′-(naphthalen-1-ylmethylene)propanehydrazide (* **13** *)*

Dark gray powder, yield 0.29 g, 58%, m.p. 176–177 °C. ^1^H NMR (400 MHz, DMSO-*d_6_*) δ: 2.50 (one part of the signal of the CH_2_CO overlaps with the DMSO-*d_6_*) and 2.96 (t, *J* = 7.0 Hz, 2H, CH_2_CO), 3.19–3.34 (m, 2H, NCH_2_), 5.03 (s, 1H, N*H*CH_2_), 6.38–6.68 (m, 4H, H_Ar_), 7.36–7.91 (m, 5H, H_Ar_), 8.00 (d, *J* = 8.4 Hz, 2H, H_Ar_), 8.43 (s, 1H, OH), 8.71 and 8.78 (2s, 1H, N=CH), and 11.38 and 11.50 (2s, 1H, CONH). ^13^C NMR (101 MHz, DMSO-*d_6_*) δ: 32.44, 34.42, 113.57, 113.61, 115.73, 123.51, 124.37, 125.53, 125.56, 126.23, 126.27, 126.58, 127.28, 127.96, 128.76, 128.84, 129.55, 130.08, 130.11, 130.14, 130.40, 133.50, 133.54, 141.43, 141.51, 142.28, 145.96, 148.41, 148.48, 167.48, and 173.14. IR (KBr): ν_max_ (cm^−1^) = 3387 (OH), 3231 (NH), and 1637 (C=O). Anal. Calcd. for C_20_H_19_N_3_O_2_, %: C, 72.05; H, 5.74; N, 12.60. Found, %: C, 71.93; H, 5.54; N, 12.39.

*General procedure for the preparation of hydrazones* **14**–**16**

To a solution of hydrazide **3** (2.6 mmol) in 1,4-dioxane (20 mL), the corresponding heterocyclic aldehyde was added (2.86 mmol) and the mixture was heated at reflux temperature for 7 h, then cooled down, and diluted with water; then, the formed precipitate was filtered off, washed with diethyl ether, and recrystallized from 2-propanol.

*3-((4-Hydroxyphenyl)amino)-N′-(thien-2-ylmethylene)propanehydrazide (* **14** *)*

Dark brown powder, yield 0.33 g, 44%, m.p. 118–119 °C. ^1^H NMR (400 MHz, DMSO-*d_6_*) δ: 2.43 and 2.79 (2t, *J* = 7.1, 7.2 Hz, 2H, CH_2_CO), 3.23 (t, *J* = 6.9 Hz, 2H, NCH_2_), 4.95 (br. s, 1H, N*H*CH_2_), 6.34–6.50 (m, 2H, H_Ar_), 6.51–6.60 (m, 2H, H_Ar_), 7.01–7.14 (m, 1H, H_Het_), 7.28–7.46 (m, 1H, H_Het_), 7.51–7.67 (m, 1H, H_Het_), 8.18 and 8.38 (2s, 1H, N=CH), 8.86 (s, 1H, OH), and 11.29 and 11.36 (2s, 1H, CONH). ^13^C NMR (101 MHz, DMSO-*d_6_*) δ: 32.29, 34.39, 112.50, 113.62, 113.69, 115.73, 115.76, 127.79, 127.85, 127.89, 128.15, 128.30, 128.64, 130.16, 130.62, 131.01, 133.82, 138.03, 138.42, 138.98, 139.17, 141.16, 141.40, 141.45, 148.38, 148.48, 148.56, 155.83, 167.34, and 172.82. IR (KBr): ν_max_ (cm^−1^) = 3343 (OH), 3191 (NH), and 1664 (C=O). Anal. Calcd. for C_14_H_15_N_3_O_2_S, %: C, 58.11; H, 5.23; N, 14.52. Found, %: C, 57.92; H, 5.03; N, 14.33.

*3-((4-Hydroxyphenyl)amino)-N′-((5-nitrothien-2-yl)methylene)propanehydrazide (* **15** *)*

Dark brown powder, yield 0.41 g, 47%, m.p. 123–124 °C. ^1^H NMR (400 MHz, DMSO-d_6_) δ: 2.41 and 2.85 (2t, *J* = 7.1, 7.2 Hz, 2H, CH_2_CO), 3.26 (t, *J* = 7.0 Hz, 2H, NCH_2_), 5.10 (s, 1H, NHCH_2_), 6.39–6.51 (m, 2H, H_Ar_), 6.53–6.61 (m, 2H, H_Ar_), 7.43–7.58 (m, 1H, H_Het_), 8.01–8.13 (m, 1H, H_Het_), 8.16 and 8.42 (2s, 1H, N=CH), 8.47 (s, 1H, OH), and 11.75 and 11.76 (2s, 1H, CONH). ^13^C NMR (101 MHz, DMSO-d_6_) δ: 31.83, 34.32, 115.80, 115.94, 128.92, 129.04, 129.40, 130.34, 130.47, 130.59, 133.46, 134.42, 134.58, 136.14, 136.18, 139.52, 143.78, 144.15, 146.70, 150.30, 152.32, 152.93, 153.86, 156.50, 156.66, 157.32 (C_Ar_), 167.94, and 173.19 (C=N, C=O). IR (KBr): ν_max_ (cm^−1^) = 3396 (OH), 3203 (NH), and 1671 (C=O). Anal. Calcd. for C_14_H_14_N_4_O_4_S, %: C, 50.29; H, 4.22; N, 16.76. Found, %: C, 50.03; H, 4.02; N, 16.53. HRMS *m*/*z* calculated for C_14_H_14_N_4_O_4_S [M + H]+: 335.0735; found: 335.0808.

*3-((4-Hydroxyphenyl)amino)-N′-((5-nitrofuryl-2-yl)methylene)propanehydrazide (* **16** *)*

Dark brown powder, yield 0.45 g, 54%, m.p. 123–124 °C. ^1^H NMR (400 MHz, DMSO-*d_6_*) δ: 2.50 (one part of the signal of the CH_2_CO overlaps with the DMSO-*d_6_*) and 2.86 (t, *J* = 7.0 Hz, 2H, CH_2_CO), 3.25 (t, *J* = 7.1 Hz, 2H, NCH_2_), 5.02 (br. s, 1H, N*H*CH_2_), 6.46 (d, *J* = 8.7 Hz, 2H, H_Ar_), 6.56 (d, *J* = 8.7 Hz, 2H, H_Ar_), 7.18 (dd, *J* = 11.6, 4.0 Hz, 1H, H_Het_), 7.76 (d, *J* = 8.5 Hz, 1H, H_Het_), 7.93 and 8.13 (2s, 1H, N=CH), 8.41 (s, 1H, OH), and 11.72 and 11.76 (2s, 1H, CONH). ^13^C NMR (101 MHz, DMSO-*d_6_*) δ: 32.06, 34.49, 113.59, 113.66, 114.66, 114.70, 114.75, 115.02, 115.75, 116.12, 123.66, 130.98, 133.89, 141.33, 141.45, 148.43, 148.53, 150.41, 150.81, 151.68, 151.73, 151.88, 155.74, 168.11, and 173.63. IR (KBr): ν_max_ (cm^−1^) = 3339 (OH), 3248 (NH), and 1664 (C=O). Anal. Calcd. for C_14_H_14_N_4_O_5_, %: C, 52.83; H, 4.43; N, 17.60. Found, %: C, 52.59; H, 4.21; N, 17.39.

*N-(2,5-dimethyl-1H-pyrrol-1-yl)-3-((4-hydroxyphenyl)amino)propanamide (* **17** *)*

To a solution of dihydrazide **3** (0.90 g, 4.6 mmol) in 2-propanol (50 mL), hexane-2,5-dione (1.05 g, 9.2 mmol) and a catalytic amount of acetic acid (0.25 mL) were added, and the mixture was heated at reflux for 2 h, then cooled down, diluted with water (20 mL), and the formed precipitate was filtered off, washed with water, and recrystallized from a mixture of 2-propanol and water.

Reddish powder, yield 0.61 g, 49%, m.p. 147–148 °C. ^1^H NMR (400 MHz, DMSO-*d_6_*) δ: 1.95 (s, 6H, 2× CH_3_), 2.50 (signal of the CH_2_CO (2H) overlaps with the DMSO-*d_6_*), 3.18–3.31 (m, 2H, NCH_2_), 5.61 (s, 2H, 2CH_Het_), 6.46 (d, *J* = 8.8 Hz, 2H, H_Ar_), 6.56 (d, *J* = 8.7 Hz, 2H, H_Ar_), 8.42 (s, 1H, OH), and 10.57 (s, 1H, NH). ^13^C NMR (101 MHz, DMSO-*d_6_*) δ: 10.97, 33.45, 40.47, 102.86, 113.68, 115.75, 126.74, 141.26, 148.56, and 170.52. IR (KBr) ν (cm^−1^) = 3343 (OH), 3219 (NH), and 1677 (C=O). Anal. Calcd. for C_15_H_19_N_3_O_2_, %: C, 65.91; H, 7.01; N, 15.37. Found, %: C, 65.72; H, 6.80; N, 15.11.

*3-((4-Hydroxyphenyl)amino)-N′-(2-oxoindolin-3-ylidene)propanehydrazide (* **18** *)*

To a solution of hydrazide **3** (1.5 mmol) in methanol (10 mL), isatin (2.55 mmol) and glacial acetic acid (3 drops) were added. The reaction mixture was heated under reflux for 30 min. Precipitate was filtered off, washed with methanol, and recrystallized from 2-propanol/H_2_O mixture. The resulting crude product was purified by column chromatography, eluting a system acetone/hexane ratio of 1:1.

Dark green powder, yield 0.31 g (64%), m.p. 180–181 °C. ^1^H NMR (400 MHz, DMSO-*d_6_*) δ: 2.61–3.08 (m, 2H, CH_2_CO), 3.29 (t, *J* = 6.9 Hz, 2H, NCH_2_), 5.08 (br. s, 1H, N*H*CH_2_), 6.26–6.71 (m, 4H, H_Ar_), 6.74–7.13 (m, 2H, H_Ar_), 7.36 (t, *J* = 7.7 Hz, 1H, H_Ar_), 7.68–8.21 (m, 1H, H_Ar_), 8.41 (br. s, 1H, OH), 10.77, (s, 1H, NHC=N), and 11.15 (s, 1H, NHN). ^13^C NMR (101 MHz, DMSO-*d_6_*) δ: 32.65 and 25.51 (*C*H_2_CO), 46.65 and 47.54 (NCH_2_), 110.57, 113.87, 115.28, 115.76, 121.63, 125.94, 132.44, 141.22, 143.62 (C_arom_), 164.68, and 172.76 (C=O). IR (KBr): ν_max_ (cm^−1^) = 3332 (OH), 3233 (NH), and 1727 and 1691 (C=O). Anal. Calcd. for C_17_H_16_N_4_O_3_, %: C, 62.95; H, 4.97; N, 17.27. Found: C, 62.71; H, 4.73; N, 17.01.

*3,*3′-((4-Hydroxyphenyl)azanediyl)bis(N′-(-(2-oxoindolin-3-yl)methylene)propanehydrazide) (** **19** *)*

To a solution of hydrazide **6** (1.07 mmol) in methanol (15 mL), isatin (4.27 mmol) and glacial acetic acid (5 drops) were added. The reaction mixture was heated under reflux for 25 min. Precipitate was filtered off, washed with methanol, and recrystallized from 2-propanol/H_2_O mixture. The resulting crude product was purified by column chromatography, eluting a system acetone/hexane ratio of 1:1.

Brownish powder, yield 0.41 g (71%), m.p. 193–194 °C. ^1^H NMR (400 MHz, DMSO-*d_6_*) δ: 2.67–3.11 (m, 4H, 2× CH_2_CO), 3.43–3.70 (m, 4H, 2× NCH_2_), 6.60–6.81 (m, 4H, H_Ar_), 6.82–6.94 (m, 2H, H_Ar_), 6.95–7.12 (m, 2H, H_Ar_), 7.18–7.41 (m, 2H, H_Ar_), 7.74–8.25 (m, 2H, H_Ar_), 8.65 (s, 1H, OH), and 10.76, 11.08, 11.21, 12.51, and 12.96 (5s, 4H, 4× NH). ^13^C NMR (101 MHz, DMSO-*d_6_*) δ: 30.97 25.51, 47.33 47.38, 110.53, 111.06, 112,20, 115.29, 115.97, 116.06, 121.62, 122.47, 124.70, 126.11, 131.37, 132.45, 138.37, 140,20, 142.30, 143.66, 164.62, and 170.88. IR (KBr): ν_max_ (cm^−1^) = 3325 (OH), 3222 (NH), and 1724 and 1689 (C=O). Anal. Calcd. for C_28_H_25_N_7_O_5_, %: C, 62.33; H, 4.67; N, 18.17. Found: C, 62.04; H, 4.43; N, 17.95.

*5,5′-(((4-Hydroxyphenyl)azanediyl)bis(ethane-2*,*1-diyl))bis(1*,*3*,*4-oxadiazole-2(3H)-thione) (***20***)*

A mixture of dihydrazyde **6** (1 g, 3.5 mmol), potassium hydroxide (0.95 g, 14 mmol), carbon disulfide (1.06 g, 14 mmol), and 50 mL methanol was refluxed for 24 h, and then the volatile fractions were separated under reduced pressure. The obtained residue was dissolved in water (30 mL), and the solution was acidified with acetic acid to pH 6. The formed solid was filtered off, washed with water, and dried. The resulting crude product was purified by column chromatography, eluting a system acetone/hexane ratio of 1:1.

White powder, yield 0.73g (72%), m.p. 156–157 °C. ^1^H NMR (400 MHz, DMSO-*d_6_*) δ: 2.86 (t, *J* = 7.1 Hz, 4H, 2CH_2_), 3.52 (t, *J* = 7.1 Hz, 4H, 2NCH_2_), 6.65 (s, 4H, H_Ar_), 8.77 (s, 1H, OH), and 14.30 (s, 2H, 2NH). ^13^C NMR (101 MHz, DMSO-*d_6_*) δ: 23.49, 47.70, 115.92, 116.57, 139.41, 150.18, 162.63 (N=C), and 177.63 (C=S). IR (KBr): ν_max_ (cm^−1^) = 3359 (OH), 3126 (NH), 1619 (C=N), 1514 (C=S), and 1164 (C-O-C). Anal. Calcd. for C_14_H_15_N_5_O_3_S_2_, %: C, 46.01; H, 4.14; N, 19.16. Found: C, 45.82; H, 3.95; N, 18.94.

*General procedure for the preparation of hydrazones* **21**–**25**.

To a boiling solution of hydrazide **6** (0.96 mmol, 0.27 g) in propan-2-ol (60 mL), the corresponding heterocyclic aldehyde (2.4 mmol) and a catalytic amount of acetic acid (1 drops) were added, and the mixture was heated under reflux for 3 h, then cooled down. The formed solid was filtered off, washed with 2-propanol and diethyl ether, and recrystallized from 2-propanol to give the appropriate title compounds **21**–**25**.

*3,3′-((4-Hydroxyphenyl)azanediyl)bis(N′-(furan-2-ylmethylene)propanehydrazide) (* **21** *)*

Dark gray powder, yield 0.22 g (52% from 1,4-dioxane), m.p. 172–173 °C. ^1^H NMR (400 MHz, DMSO-*d_6_*) δ: 2.33–2.45 (m, 2H, CH_2_), 2.68–2.83 (m, 2H, CH_2_), 3.40–3.58 (m, 4H, 2NCH_2_), 6.54–6.90 (m, 8H, H_Ar_, H_Het_.), 7.73–8.05 (m, 4H, H_Het_, 2CH), 8.50–8.71 (m, 1H, OH), and 11.20, 11.31, and 11.33 (3s, 2H, 2NH). ^13^C NMR (101 MHz, DMSO-*d_6_*) δ: 30.46, 30.67, 32.43, 32.62, 46.97, 47.70, 47.86, 112.05, 112.10, 112.99, 113.09, 113.18, 113.23, 114.43, 115.03, 115.90, 116.28, 133.04, 135.93, 140.37, 144.73, 144.98, 148.78, 149.09, 149.27, 149.40, 167.33, 167.39, and 172.90. IR (KBr): ν_max_ (cm^−1^) = 3363 (OH), 3181 (NH), and 1659 (C=O). Anal. Calcd. for C_22_H_23_N_5_O_5_, %: C, 60.40; H, 5.30; N, 16.01. Found: C, 60.19; H, 5.15; N, 15.82.

*3,3′-((4-Hydroxyphenyl)azanediyl)bis(N′-(thien-2-ylmethylene)propanehydrazide) (* **22** *)*

Light lilac powder, yield 0.29 g (65% from 1,4-dioxane), m.p. 108–109 °C. ^1^H NMR (400 MHz, DMSO-*d_6_*) δ: 2.32–2.45 (m, 2H, CH_2_), 2.69–2.82 (m, 2H, CH_2_), 3.40–3.58 (m, 4H, 2NCH_2_), 6.68 (br. s. 4H, H_Ar_), 7.05–7.14 (m, 2H, H_Het_.), 7.34–7.42 (m, 2H, H_Het_.), 7.53–7.66 (m, 2H, H_Het_.), 8.17 (d, *J* = 5.4 Hz, 1H, CH), 8.35 (s, 1H, CH), 8.60, 8.63, and 8.69 (3s, 1H, OH), and 11.29, 11.32, and 11.33 (3s, 2H, 2NH). ^13^C NMR (101 MHz, DMSO-*d_6_*) δ: 30.34, 30.54, 32.41, 32.58, 47.12, 47.22, 47.63, 47.81, 114.53, 115.14, 115.86, 115.95, 116.00, 116.19, 127.77, 127.86, 127.89, 128.08, 128.11, 128.66, 130.09, 130.16, 130.66, 138.10, 138.16, 138.93, 138.97, 139.08, 139.10, 140.41, 140.43, 140.51, 141.22, 148.87, 149.16, 149.65, 167.19, 167.26, 172.68, and 172.71. IR (KBr): ν_max_ (cm^−1^) = 3349 (OH), 3218 (NH), and 1661 (C=O). Anal. Calcd. for C_22_H_23_N_5_O_3_S_2_, %: C, 56.27; H, 4.94; N, 14.91. Found: C, 56.03; H, 4.71; N, 14,52.

*3,3′-((4-Hydroxyphenyl)azanediyl)bis(N′-(-(5-nitrothien-2yl)methylene)propanehydrazide) (* **23** *)*

Brownish powder, yield 0.34 g (63%), m.p. 205–206 °C. ^1^H NMR (400 MHz, DMSO-*d_6_*) δ: 2.44 and 2.78 (2q, *J* = 8.3, 8.7 Hz, 4H, 2× CH_2_CO), 3.39–3.59 (m, 4H, 2× NCH_2_), 6.68 (s, 4H, H_Ar_), 7.32–7.57 (m, 2H, H_Ar_), 7.95–8.17 (m, 3H, H_Ar_), 8.32–8.47 (m, 1H, H_Ar_), 8.55–8.78 (m, 1H, OH), and 11.66–11.81 (m, 2H, 2× NH). ^13^C NMR (101 MHz, DMSO-*d_6_*) δ: 30.45, 47.28, 115.15, 115.41, 115.88, 115.95, 116.47, 128.80, 128.89, 129.32, 139.39, 130.44, 130.50, 136.20, 136.29, 139.59, 140.32, 140.40, 140.45,146.59, 146.63, 146,67, 149.30, 149.40, 150.29, 150.64, 167.99, and 173.34 (CH=N, C=O). IR (KBr): ν_max_ (cm^−1^) = 3322 (OH), 3151 (NH), and 1673 (C=O). Anal. Calcd. for C_22_H_21_N_7_O_7_S_2_, %: C, 47.22; H, 3.78; N, 17.52. Found: C, 47.00; H, 3.49; N, 17.23.

*3,3′-((4-Hydroxyphenyl)azanediyl)bis(N′-((5-nitrofuran-2-yl)methylene)propanehydrazide) (* **24** *)*

Brown powder, yield 0.35 g (70%, from 1,4-dioxane), m.p. 209–210 °C. ^1^H NMR (400 MHz, DMSO-*d_6_*) δ: 2.38–2.52 (m, 2H, CH_2_), 2.72–2.87 (m, 2H, CH_2_), 3.39–3.61 (m, 4H, 2NCH_2_), 6.67 (br. s. 4H, H_Ar_), 7.03–7.23 (m, 2H, H_Het_.), 7.65–7.78 (m, 2H, H_Het_.), 7.87 and 7.90 (2s, 1H, CH), 8.09 (s, 1H, CH), 8.60, 8.64, and 8.70 (3s, 1H, OH), and 11.69, 11.71, and 11.76 (3s, 2H, 2NH). ^13^C NMR (101 MHz, DMSO-*d_6_*) δ: 30.24, 30.43, 32.52, 32.72, 47.04, 47.64, 47.73, 114.23, 114.36, 114.62, 114.98, 115.07, 115.48, 115.94, 116.46, 130.87, 131.03, 133.93, 140.33, 140.44, 149.15, 149.36, 151.59, 151.69, 151.81, 151.84, 168.05, and 173.57. IR (KBr): ν_max_ (cm^−1^) = 3368 (OH), 3123 (NH), and 1685 (C=O). Anal. Calcd. for C_22_H_21_N_7_O_9_, %: C, 50.10; H, 4.01; N, 18.59. Found: C, 49.90; H, 3.84; N, 18.27.

*3,3′-((4-Hydroxyphenyl)azanediyl)bis(N′-(thien-3-ylmethylene)propanehydrazide) (* **25** *)*

Light lilac powder, yield 0.33 g (73% from 1,4-dioxane), m.p. 191–192 °C. ^1^H NMR (400 MHz, DMSO-*d_6_*) δ: 2.35–2.46 (m, 2H, CH_2_), 2.74–2.87 (m, 2H, CH_2_), 3.41–3.59 (m, 4H, 2NCH_2_), 6.62–6.73 (m, 4H, H_Ar_), 7.34–7.44 (m, 2H, H_Het_.), 7.51–7.62 (m, 2H, H_Het_.), 7.77–7.88 (m, 2H, H_Het_.), 8.02 and 8.17 (2s, 1H, 2CH), 8.63, 8.64, and 8.68 (3s, 1H, OH), and 11.21, 11.24, and 11.26 (3s, 2H, 2NH). ^13^C NMR (101 MHz, DMSO-*d_6_*) δ: 30.27, 30.46, 32.43, 32.62, 47.18, 47.34, 47.58, 47.84, 114.66, 115.19, 115.86, 115.89, 124.42, 124.46, 124.66, 127.32, 127.35, 127.43, 127.47, 127.53, 127.87, 137.36, 137.49, 138.73, 138.76, 140.50, 141.74, 141.77, 148.97, 149.20, 149.64, 167.26, 167.31, 172.92, and 172.94. IR (KBr): ν_max_ (cm^−1^) = 3392 (OH), 3168 (NH), and 1652 (C=O). Anal. Calcd. for C_22_H_23_N_5_O_3_S_2_, %: C, 56.27; H, 4.94; N, 14.91. Found: C, 59.98; H, 4.65; N, 14.64.

*General procedure for the preparation of hydrazones* **26**–**28**.

A mixture of hydrazide **4** (1.1 mmol, 0.3 g) and the corresponding ketone (acetone, 2-butanone, or acetophenone) (15 mL) was heated under reflux for 5 h. After completion of the reaction, the mixture was cooled down, diluted with water (35 mL), and left in the refrigerator for 24 h. Then, the formed precipitate was filtered off, washed with acetone and diethyl ether, and recrystallized from acetone to give the title compound **26** and **28**. Product **27** was separated from the reaction mixture by evaporating the volatile fractions under reduced pressure, diluting the residue with diethyl ether, filtering the obtained solid, and recrystallizing from 2-butanone to give the title compound **27**.

*3,3′-((4-Hydroxyphenyl)azanediyl)bis(N′-(propan-2-ylidene)propanehydrazide) (* **26** *)*

Orange powder, yield 0.34 g, 86%, m.p. 172–173 °C. ^1^H NMR (400 MHz, DMSO-*d_6_*) δ: 1.83 and 1.90 (2s, 12H, 4× CH_3_), 2.40 and 2.67 (2q, *J* = 7.3, 7.6 Hz, 4H, 2× CH_2_CO), 3.35–3.51 (m, 4H, 2× NCH_2_), 6.56–6.71 (m, 4H, H_Ar_), 8.55, 8.59, and 8.65 (3s, 1H, OH), 9.96 and 9.97 (2s, 1H, NH), and 10.02 (s, 1H, NH). ^13^C NMR (101 MHz, DMSO-*d_6_*) δ: 17.01, 17.48, 24.95, 25.11, 30.70, 30.97, 32.01, 32.25, 46.98, 47.90 (2× NCH_2_), 114.35, 115.02, 115.79, 116.04, 140.53, 140.70, 148.66, 148.99, 149.49, 150.14, 150.19, 154.64, 154.66, 167.19, 167.28, 173.09, and 173.12. IR (KBr): ν_max_ (cm^−1^) = 3225 (OH), 3147 (NH), and 1675 (C=O). Anal. Calcd. for C_18_H_27_N_5_O_3_, %: C, 59.81; H, 7.53; N, 19.38. Found, %: C, 59.60; H, 7.31; N, 19.04.

*3,3′-((4-Hydroxyphenyl)azanediyl)bis(N′-(butan-2-ylidene)propanehydrazide) (* **27** *)*

White solid, yield 0.043 g, 10%, m.p. 76–77 °C. ^1^H NMR (400 MHz, DMSO-*d_6_*) δ: 1.00 (t, *J* = 7.1 Hz, 6H, CH_2_C*H_3_*), 1.81 and 1.88 (2s, 6H, 2× CH_3_), 2.20 (q, *J* = 7.5 Hz, 4H, C*H_2_*CH_3_), 2.41 and 2.69 (2q, *J* = 6.8, 6.9 Hz, 4H, 2× CH_2_CO), 3.35–3.49 (m, 4H, 2× NCH_2_), 6.57–6.73 (m, 4H, H_Ar_), 8.55, 8.59, and 8.65 (3s, 1H, OH), and 9.97, 10.02, and 10.13 (3s, 2H, NH). ^13^C NMR (101 MHz, DMSO-*d_6_*) δ: 9.74, 9.77, 10.59, 10.60, 10.85, 15.69, 15.88, 30.63, 30.70, 30.88, 30.95, 31.47, 31.52, 32.01, 32.04, 32.19, 32.23, 47.07, 47.86, 114.36, 114.99, 115.08, 115.77, 116.01, 140.53, 142.23, 148.69, 148.95, 149.03, 149.49, 153.67, 153.74, 158.15, 158.22,158.39, 167.19, 167.29, 167.37, and 173.23. IR (KBr): ν_max_ (cm^−1^) = 3392 (OH), 3244 (NH), and 1662 (C=O). Anal. Calcd. for C_20_H_31_N_5_O_3_, %: C, 61.67; H, 8.02; N, 17.98. Found, %: C, 61.40; H, 7.81; N, 17.69.

*3,3′-((4-Hydroxyphenyl)azanediyl)bis(N′-(1-phenylethylidene)propanehydrazide) (* **28** *)*

White powder, yield 0.40 g, 76%, m.p. 203–204 °C. ^1^H NMR (400 MHz, DMSO-*d_6_*) δ: 2.13–2.30 (m, 6H, 2× CH_3_), 2.57 and 2.88 (2t, *J* = 7.3, 7.4 Hz, 4H, 2× CH_2_CO), 3.40–3.66 (m, 4H, 2× NCH_2_), 6.52–6.79 (m, 4H, H_Ar_), 7.26–7.48 (m, 6H, H_Ar_), 7.60–7.81 (m, 4H, H_Ar_), 8.62, 8.66, and 8.70 (3s, 1H, OH), and 10.38 and 10.49 (2s, 2H, NH). ^13^C NMR (101 MHz, DMSO-*d_6_*) δ: 13.66, 13.69, 14.08, 30.68, 30.90, 32.23, 32.40, 47.45, 47.88, 114.88, 115.52, 115.86, 125.96, 125.99, 126.17, 128.25, 128.32, 128.35, 128.88, 129.10, 138.21, 138.28, 140.52, 147.46, 147.53, 149.00, 149.29, 150.92, 151.01, 167.90, 168.01, 173.93, and 174.00. IR (KBr): ν_max_ (cm^−1^) = 3418 (OH), 3193 (NH), and 1667 (C=O). Anal. Calcd. for C_28_H_31_N_5_O_3_, %: C, 69.26; H, 6.44; N, 14.42. Found, %: C, 69.02; H, 6.20; N, 14.19.

*General procedure for the preparation of dihydrazones* **29**–**35**

To a solution of dihydrazide **6** (0.89 mmol) in 2-propanol (50 mL), the corresponding aromatic aldehyde was added (2.22 mmol), and the mixture was heated under reflux for 2 h, then cooled down, and the formed precipitate was filtered off, washed with 2-propanol and diethyl ether, and recrystallized from 2-propanol or 1,4-dioxane.

*3,3′-((4-Hydroxyphenyl)azanediyl)bis(N′-(benzylidene)propanehydrazide) (* **29** *)*

White powder, yield 0.30 g, 73%, m.p. 203–204 °C (from 2-propanol). ^1^H NMR (400 MHz, DMSO-*d_6_*) δ: 2.43 and 2.84 (2q, *J* = 7.3, 7.4 Hz, 4H, 2× CH_2_CO), 3.41–3.63 (m, 4H, 2× NCH_2_), 6.61–6.76 (m, 4H, H_Ar_), 7.32–7.45 (m, 6H, H_Ar_), 7.54–7.69 (m, 4H, H_Ar_), 7.95–8.01 and 8.11–8.17 (2m, 2H, N=CH), 8.65, 8.66, and 8.70 (3s, 1H, OH), and 11.32, 11.38, and 11.39 (3s, 2H, NH). ^13^C NMR (101 MHz, DMSO-*d_6_*) δ: 30.31, 30.52, 32.43, 32.64, 47.30, 47.41, 47.65, 47.90, 114.79, 115.27, 115.88, 116.32, 126.65, 126.68, 126,98, 134.20, 134.30 140.46, 140.53, 142.96, 140.53, 142.96, 142.99, 145.98, 146.04, 149.03, 149.24, 167.34, 167.42, 173.08, and 173.12. IR (KBr): ν_max_ (cm^−1^) = 3373 (OH), 3181 (NH), and 1686 (C=O). Anal. Calcd. for C_26_H_27_N_5_O_3_, %: C, 68.25; H, 5.95; N, 15.31. Found, %: C, 68.03; H, 5.71; N, 15.08.

*3,3′-((4-Hydroxyphenyl)azanediyl)bis(N′-(4-nitrobenzylidene)propanehydrazide) (* **30** *)*

Reddish brown powder, yield 0.41g (86% from 1,4-dioxane), m.p. 164 °C (decomp.). ^1^H NMR (400 MHz, DMSO-*d_6_*) δ: 2.40–2.51 (m, 2H, CH_2_), 2.81–2.92 (m, 2H, CH_2_), 3.46–3.58 (m, 4H, 2NCH_2_), 6.63–6.74 (m, 4H, H_Ar_), 7.71–8.27 (m, 10H, H_Ar_, 2CH), 8.70 (d, *J* = 7.5 Hz 1H, OH), and 11.61 and 11.68 (2s, 2H, 2NH). ^13^C NMR (101 MHz, DMSO-*d_6_*) δ: 30.15, 30.37, 32.47, 32.72, 47.51, 115.18, 115.52, 115.87, 116.42, 123.91, 123.94, 127.47, 127.52, 127.85, 140.46, 140.49, 140.67, 143.50, 147.50, 147.70, 149.28, 149.41, 167.86, and 173.62. IR (KBr): ν_max_ (cm^−1^) = 3348 (OH), 3182 (NH), and 1655 (C=O). Anal. Calcd. for C_26_H_25_N_7_O_7_, %: C, 57.04, H, 4.60, N, 17.91. Found: C, 56.90, H, 4.29, N, 17.70.

*3,3′-((4-Hydroxyphenyl)azanediyl)bis(N′-(benzylidene)propanehydrazide) (* **31** *)*

Light violet powder, yield 0.27 g, 66%, m.p. 213–214 °C (from 2-propanol). ^1^H NMR (400 MHz, DMSO-*d_6_*) δ: 2.42 and 2.82 (2q, *J* = 7.1, 7.3 Hz, 4H, 2× CH_2_CO), 3.39–3.61 (m, 4H, 2× NCH_2_), 6.57–6.75 (m, 4H, H_Ar_), 7.38–7.50 (m, 4H, H_Ar_), 7.51–7.61 (m, 3H, H_Ar_), 7.63–7.72 (m, 1H, H_Ar_), 7.86–7.99 and 8.06–8.15 (2m, 2H, N=CH), 8.65–8.72 (m, 1H, OH), and 11.38 and 11.45 (2s, 2H, NH). ^13^C NMR (101 MHz, DMSO-*d_6_*) δ: 30.20, 30.41, 32.41, 32.64, 47.39, 47.46, 47.64, 47.84, 114.94, 115.36, 115.86, 116.30, 128.24, 128.28, 128.59, 128.80, 128.83, 129.37, 131.18, 133.12, 133.25, 134.06, 134.31, 140.45, 140.50, 141.62, 144.66, 149.10, 149.28, 167.44, 167.49, 173.20, and 173.22. IR (KBr): ν_max_ (cm^−1^) = 3367 (OH), 3196 (NH), and 1670 (C=O). Anal. Calcd. for C_26_H_25_Cl_2_N_5_O_3_, %: C, 59.32; H, 4.79; N, 13.30. Found, %: C, 59.10; H, 4.52; N, 13.07.

*3,3′-((4-Hydroxyphenyl)azanediyl)bis(N′-(4-(dimethylamino)benzylidene)propanehydrazide) (* **32** *)*

Brownish powder, yield 0.15 g, 37%, m.p. 84 °C (decomp.). ^1^H NMR (400 MHz, DMSO-*d_6_*) δ: 2.29–2.43 and 2.73–2.85 (2m, 4H, 2× CH_2_CO), 2.86–2.99 (m, 12H, 4× CH_3_), 3.39–3.61 (m, 4H, 2× NCH_2_), 6.58–6.75 (m, 7H, H_Ar_), 7.33–7.51 (m, 4H, H_Ar_), 7.58–7.75 (m, 1H, H_Ar_), 7.86 and 7.99 (2s, 2H, N=CH), 8.59–8.72 (m, 1H, OH), and 10.96–11.13 (m, 2H, NH). ^13^C NMR (101 MHz, DMSO-*d_6_*) δ: 39.65, 30.38, 30.58, 32.42, 32.58, 47.22, 47.31, 47.71, 48.02, 111.06, 111.75, 111.81, 114.60, 115.14, 115.87, 116.29, 121.60, 121.65, 124.52, 127.91, 127.94, 128.28, 128.31, 131.54, 140.59, 143.80, 143.85, 146.79, 146.87, 148.87, 149.12, 151.22, 151.38, 154.20, 166.68, 166.78, 172.46, and 172.48. IR (KBr): ν_max_ (cm^−1^) = 3341 (OH), 3204 (NH), and 1656 (C=O). Anal. Calcd. for C_30_H_37_N_7_O_3_, %: C, 66.28; H, 6.86; N, 18.03. Found, %: C, 66.00; H, 6.63; N, 17.80.

*3,3′-((4-Hydroxyphenyl)azanediyl)bis(N′-(4-hydroxybenzylidene)propanehydrazide) (* **33** *)*

Lilac powder, yield 0.23g (54% from 2-propnaol), m.p. 165 °C (decomp.). ^1^H NMR (400 MHz, DMSO-*d_6_*) δ: 2.31–2.44 (m, 2H, CH_2_), 2.73–2.86 (m, 2H, CH_2_), 3.40–3.59 (m, 4H, 2NCH_2_), 6.58–6.88 (m, 8H, H_Ar_), 7.37–7.53 (m, 4H, H_Ar_), 7.89 and 8.02 (2s, 2H, 2CH), 8.65 (s, 1H, OH), 9.85 (s, 2H, 2OH), and 11.07–11.34 (m, 2H, 2NH). ^13^C NMR (101 MHz, DMSO-*d_6_*) δ: 30.37, 30.58, 32.45, 32.66, 47.27, 47.36, 47.74, 47.99, 114.59, 115.14, 115.65, 115.71, 115.90, 116.30, 125.26, 128.38, 128.41, 128.53, 128.76, 128.84, 140.52, 140.59, 140.62, 143.35, 146.36, 148.93, 149.16, 149.69, 159.08, 159.27, 167.00, 167.10, 172.71, and 172.75. IR (KBr): ν_max_ (cm^−1^) = 3398 (OH), 3176 (NH), and 1681 (C=O). Anal. Calcd. for C_26_H_27_N_5_O_5_, %: C, 63.79; H, 5.56; N, 14.31. Found: C, 63.51; H, 5.32; N, 14.10.

*3,3′-((4-Hydroxyphenyl)azanediyl)bis(N′-(3,4,5-trimethoxybenzylidene)propanehydrazide) (* **34** *)*

White powder, yield 0.31 g, 76%, m.p. 194–195 °C (from 2-propanol). ^1^H NMR (400 MHz, DMSO-*d_6_*) δ: 2.41 and 2.84 (2t, *J* = 7.3 Hz, 4H, 2× CH_2_CO), 3.41–3.59 (m, 4H, 2× NCH_2_), 3.61–3.87 (m, 18H, OCH_3_), 6.55–6.76 (m, 4H, H_Ar_), 6.81–7.01 (m, 4H, H_Ar_), 7.85 and 7.90 (2s, 1H, N=CH), 8.05 (d, *J* = 6.3 Hz, 1H, N=CH), 8.59–8.70 (m, 1H, OH), and 11.33 and 11.35 (2s, 2H, NH). ^13^C NMR (101 MHz, DMSO-*d_6_*) δ: 30.20, 30.36, 32.43, 32.58, 47.46, 47.55, 47.66, 47.80, 55.77, 55.85, 55.91, 103.76, 103.89, 104.17, 115.39, 115.66, 115.80, 116.09, 129.70, 129.81, 138.83, 139.04, 140.51, 142.71, 145.99, 149.24, 149.37, 153.07, 153.13, 167.28, 167.34, 173.08, and 173.13. IR (KBr): ν_max_ (cm^−1^) = 3342 (OH), 3211 (NH), and 1667 (C=O). Anal. Calcd. for C_32_H_39_N_5_O_9_, %: C, 60.27; H, 6.16; N, 10.98. Found, %: C, 60.00; H, 5.92; N, 10.55.

*3,3′-((4-Hydroxyphenyl)azanediyl)bis(N′-(naphthalen-1-ylmethylene)propanehydrazide) (* **35** *)*

Brownish powder, yield 0.18 g, 44%, m.p. 112–113 °C (from 2-propanol). ^1^H NMR (400 MHz, DMSO-*d_6_*) δ: 2.50 (signal of the CH_2_CO (2H) overlaps with the DMSO-*d_6_*) and 2.88–2.99 (m, 2H, CH_2_CO), 3.47–3.68 (m, 4H, 2× NCH_2_), 6.59–6.81 (m, 4H, H_Ar_), 7.45–7.69 (m, 6H, H_Ar_), 7.73–8.08 (m, 6H, H_Ar_), 8.47, 8.49, and 5.52 (3s, 1H, OH), 8.66, 8.68, 8.71, and 8.72 (4s, 2H, N=CH), 8.73–8.86 (m, 2H, H_Ar_), and 11.38 and 11.50 (2s, 2H, NH). ^13^C NMR (101 MHz, DMSO-*d_6_*) δ: 30.40, 30.58, 32.48, 32.65, 47.40, 47.44, 47.80, 47.96, 115.27, 115.59, 115.90, 116.36, 123.38, 124.32, 125.49, 126.14, 126.23, 126.46, 127.12, 127.27, 127.91, 128.74, 128.78, 129.42, 129.53, 130.07, 130.10, 130.38, 133.43, 133.50, 140.49, 140.53, 142.33, 145.96, 146.03, 149.19, 149.36, 167.35, 167.44, 173.05, and 173.10. IR (KBr): ν_max_ (cm^−1^) = 3335 (OH), 3207 (NH), and 1671 (C=O). Anal. Calcd. for C_34_H_31_N_5_O_3_, %: C, 73.23; H, 5.60; N, 12.56. Found, %: C, 72.95; H, 5.35; N, 12.22.

*3,3′-((4-Hydroxyphenyl)azanediyl)bis(1-(3,5-dimethyl-1H-pyrazol-1-yl)propan-1-one) (* **36** *)*

To a solution of dihydrazide **6** (0.5 g, 1.8 mmol) in 2-propanol (28 mL), pentane-2,4-dione (0.90 g, 9.0 mmol) and a catalytic amount of hydrochloric acid (0.05 mL) were added, and the mixture was heated under reflux for 5 h, then cooled down. The solvent was removed under reduced pressure, the residue was poured with water (30 mL), and the formed precipitate was filtered off, washed with water and diethyl ether, and recrystallized from a mixture of 2-propanol and water.

Violet powder, yield 0.50 g (84%), m. p. 77–78 °C (decomp.). ^1^H NMR (400 MHz, DMSO-*d_6_*) δ: 2.15 and 2.44 (2s, 12H, 4× CH_3_C), 3.20 (t, *J* = 7.1 Hz, 4H, 2× CH_2_CO), 3.59 (t, *J* = 7.1 Hz, 4H, 2× NCH_2_), 6.15 (s, 2H, 2× CH_Het_), 6.71 (d, *J* = 8.9 Hz, 2H, H_Ar_), and 8.65 (s, 1H, OH). ^13^C NMR (101 MHz, DMSO-*d_6_*) δ: 13.43, 14.07, 33.20, 47.06, 111.12, 115.54, 115.84, 140.18, 143.10, 149.36, 151.33, and 172.20. IR (KBr) ν (cm^−1^) = 3370 (OH), 3108 (NH), and 1724 (C=O). Anal. Calcd. for C_22_H_27_N_5_O_3_, %: C, 64.53; H, 6.65; N, 17.10. Found, %: C, 64.27; H, 6.41; N, 16.96.

*3,3′-((4-Hydroxyphenyl)azanediyl)bis(N-(2,5-dimethyl-1H-pyrrol-1-yl)propanamide)* (**37**)

To a solution of dihydrazide **6** (0.3 g, 1.1 mmol) in 2-propanol (17 mL), hexane-2,5-dione (0.50 g, 4.4 mmol) and a catalytic amount of acetic acid (0.05 mL) were added, and the mixture was heated under reflux for 5 h, then cooled down, and diluted with water (20 mL); the formed precipitate was filtered off, washed with water, and recrystallized from a mixture of 2-propanol and water.

Brownish powder, yield 0.30 g (63% from 2-propanol and water mixture), m. p. 178 °C (decomp.). ^1^H NMR (400 MHz, DMSO-*d_6_*) δ: 1.95 (s, 12H, 4× CH_3_), 2.50 (signal of the CH_2_CO (4H) overlaps with the DMSO-d_6_), 3.36–3.65 (m, 4H, 2× NCH_2_), 5.62 and 5.67 (2s, 4H, 4CH_pyr_), 6.48–6.82 (m, 4H, H_Ar_), 8.72 (s, 1H, OH), and 10.60 (s, 2H, 2× NH). ^13^C NMR (101 MHz, DMSO-*d_6_*) δ: 10.94, 31.40, 47.82, 102.87, 115.89, 116.61, 126.68, 140.38, 149.86, and 170.42. IR (KBr) ν (cm^−1^) = 3351 (OH), 3257 (NH), and 1671 (C=O). Anal. Calcd. for C_24_H_31_N_5_O_3_, %: C, 65.88; H, 7.14; N, 16.01. Found, %: C, 65.66; H, 6.97; N, 15.82.

**Microbial strains and culture conditions**

The multidrug-resistant *S. aureus* strain TCH 1516 [USA 300-HOU-MR] was obtained from the American Type Culture Collection (ATCC). Vancomycin-resistant *E. faecalis* and drug-resistant *Candida* species strains were acquired from the Centers for Disease Control and Prevention (CDC) AR isolate bank. Prior to the initiation of this study, all microbial strains were stored in commercial cryopreservation systems at a temperature of −80 °C. The strains were cultivated on Columbia sheep blood agar (Becton Dickinson, Franklin Lakes, NJ, USA), or potato dextrose agar (PDA) for *Candida* (Becton Dickinson, USA).

**Minimal inhibitory concentration determination**

The antimicrobial activity of 3-((4-hydroxyphenyl)amino)propanoic acid derivatives was assessed using the broth microdilution method, following the guidelines outlined by the Clinical Laboratory Standards Institute (CLSI), with modifications [18]. In brief, the compounds were dissolved in dimethylsulfoxide (DMSO) to attain a final concentration of 25–30 mg/mL. Dilution series were prepared in deep 96-well microplates to achieve a two-fold concentration range of 0.25, 0.5, 1, 2, 4, 8, 16, 32, and 64 µg/mL, utilizing cation-adjusted Mueller–Hinton broth (CAMHB) as the growth medium. For *Candida* screening, dilutions were performed in RMPI/MOPS media. The microplates containing the dilution series were then inoculated with fresh cultures of each tested organism to reach a final concentration of 5 × 10^4^ CFU (colony-forming units) of the test organism in media containing 1% DMSO and 1× drug concentration, with a volume of 200 µL per well. Wells that were inoculated with media containing 1% DMSO served as positive controls. Subsequently, the microplates were incubated at 35 ± 1 °C for 18 ± 2 h. Following the incubation period, the plates were examined using a manual microplate viewer (Sensititre Manual Viewbox, United States). The minimal inhibitory concentration (MIC) was defined as the lowest concentration (µg/mL) of the tested drug that completely inhibited the growth of the test organism. All experiments were conducted in duplicate with three technical replicates for each condition.

**In silico ADME prediction**

The structures of selected compounds were converted to SMILE structures and used for in silico ADME prediction using SwissAdme online tool (http://www.swissadme.ch/faq.php, accessed on 1 January 2024). 

## 5. Conclusions

This study demonstrates that novel substituted amino acid derivatives, derived from 3-((4-hydroxyphenyl)amino)propanoic acid, exhibit promising antimicrobial activity against ESKAPE group pathogens and drug-resistant fungal strains, including *C. auris*. The investigation underscores that amino acid derivatives bearing a 4-hydroxyphenyl core, along with heterocyclic substituents, show potent broad-spectrum antibacterial and antifungal activity and drug-like ADME properties. This study provides foundational and synthetic procedures for subsequent compound generation, paving the way for hit-to-lead optimization and early and structure–activity relation data. Further investigations are crucial to enhance our understanding of the in vitro and in vivo safety, biological availability, and tolerability of 3-((4-hydroxyphenyl)amino)propanoic acid derivatives, as well as additional compounds based on this pharmacophore. 

## Figures and Tables

**Table 1:** The in vitro antimicrobial activity of novel 3-((4-hydroxyphenyl)amino)propanoic acid derivatives **2**–**37** against multidrug-resistant bacterial pathogens.

	Minimal Inhibitory Concentration (µg/mL)					
Compound	*S. aureus* TCH 1516 ^1^	*E. faecalis* AR-0671 ^2^	*E. coli* AR-0001 ^3^	*K. pneumoniae* AR-0003 ^4^	*P. aeruginosa* AR-1114 ^5^	*A. baumannii* AR-0273 ^6^
**2**	64	64	>64	>64	>64	>64
**3**	>64	>64	>64	>64	>64	>64
**4**	>64	>64	>64	>64	>64	>64
**5**	>64	>64	>64	>64	>64	>64
**6**	>64	>64	64	64	32	32
**7**	32	>64	64	64	>64	>64
**8**	16	8	16	>64	>64	>64
**9**	8	8	64	>64	>64	32
**10**	>64	>64	>64	>64	>64	>64
**11**	>64	>64	>64	>64	>64	>64
**12**	>64	>64	>64	>64	>64	>64
**13**	16	16	32	64	>64	64
**14**	8	8	64	64	64	64
**15**	1	<0.5	8	32	64	16
**16**	4	2	16	>64	>64	64
**17**	>64	>64	>64	>64	>64	16
**18**	>64	>64	>64	>64	>64	>64
**19**	>64	>64	>64	>64	>64	>64
**20**	32	32	>64	>64	>64	>64
**21**	16	16	32	64	>64	64
**22**	32	16	32	32	64	64
**23**	>64	>64	>64	>64	>64	>64
**24**	32	32	64	64	64	32
**25**	64	32	>64	>64	>64	>64
**26**	>64	>64	>64	>64	>64	>64
**27**	>64	>64	>64	>64	>64	>64
**28**	>64	>64	32	64	>64	64
**29**	16	>64	64	64	>64	>64
**30**	16	16	32	64	>64	>64
**31**	16	16	32	64	64	32
**32**	>64	>64	>64	>64	>64	16
**33**	8	16	32	64	16	16
**34**	>64	>64	>64	>64	>64	>64
**35**	>64	>64	>64	>64	>64	>64
**36**	32	64	>64	>64	>64	>64
**37**	16	32	16	>64	>64	>64
**Vancomycin**	2	4	N/A	N/A	N/A	N/A
**Cefazolin**	8	8	>64	>64	>64	>64

^1.^*S. aureus* TCH 1516, *mecA* MRSA isolate, USA300 lineage. ^2.^ *E. faecalis* AR-0671, *ant(6)-la*, *aph(3′)-III*, *erm(B)*, *sat-4A*, *tet(M)*. ^3.^ *E. coli* AR-0001, *aac(6′)-Ib-cr*, *aadA5*, *ACRF*, *catB4*, *dfrA17*, *KPC-3*, *MDF(A)*, *mph(A)*, *OXA-1*, *sul1*, *tet(A)*, *tet(R)*. ^4.^ *K. pneumoniae AR-0003*, *aac(6′)-Ib*, *aadA1*, *aph(3′)-Ib*, *aph(6)-Id*, *dfrA14*, *EMRD*, *KDEA*, *KPC-3*, *Omp35*, *OmpK35*, *oqxA*, *oqxB*, *OXA-9*, *SHV-12*, *sul2*, *TEM-1*. ^5.^ *P. aeruginosa* AR-1114, *aac(6′)-Il*, *aacA4*, *aadA1*, *aadA6*, *bcr1*, *catB7*, *IMP-15*, *mexE*, *mexX*, *OXA-2*, *OXA-494*, *PDC-5*, *sul1*. ^6.^ *A. baumannii* AR-0273, *aac(3)-IIe*, *ABAF*, *ADC-25*, *ADEC*, *aph(3′)-Ia*, *aph(6)-Id*, *aphA6*, *OXA-23*, *OXA-66*, *strA*, *sul2*, *tet(B)*. N/A—not applicable.

**Table 2:** The in vitro antifungal activity of novel 3-((4-hydroxyphenyl)amino)propanoic acid derivatives **2**–**37** against drug-resistant fungal pathogens.

	Minimal inhibitory Concentration (µg/mL)					
Compound	*C. albicans* AR-0761 ^1^	*C. parapsilosis * AR-0339 ^2^	*C. auris * AR-0381 ^3^	*C. auris * AR-0382 ^4^	*C. auris * AR-0383 ^5^	*C. auris * AR-0384 ^6^
**2**	>64	>64	>64	>64	>64	>64
**3**	>64	>64	>64	>64	>64	>64
**4**	>64	>64	>64	>64	>64	>64
**5**	>64	>64	>64	>64	>64	>64
**6**	>64	>64	>64	>64	>64	>64
**7**	>64	>64	>64	>64	>64	>64
**8**	>64	>64	>64	>64	>64	>64
**9**	>64	>64	>64	>64	>64	>64
**10**	>64	>64	>64	>64	>64	>64
**11**	>64	>64	>64	>64	>64	>64
**12**	>64	>64	>64	>64	>64	>64
**13**	>64	>64	>64	>64	>64	>64
**14**	16	32	16	16	8	16
**15**	32	>64	>64	>64	>64	>64
**16**	8	16	32	32	32	32
**17**	8	8	16	16	16	16
**18**	>64	>64	>64	>64	>64	>64
**19**	>64	>64	>64	>64	>64	>64
**20**	>64	>64	>64	>64	>64	>64
**21**	>64	>64	>64	>64	>64	>64
**22**	>64	>64	>64	>64	>64	>64
**23**	>64	>64	>64	>64	>64	>64
**24**	>64	>64	>64	>64	>64	>64
**25**	>64	>64	>64	>64	>64	>64
**26**	>64	>64	>64	>64	>64	>64
**27**	>64	>64	>64	>64	>64	>64
**28**	>64	>64	>64	>64	>64	>64
**29**	>64	>64	>64	>64	>64	>64
**30**	>64	>64	>64	>64	>64	>64
**31**	>64	>64	>64	>64	>64	>64
**32**	>64	>64	>64	>64	>64	>64
**33**	>64	>64	>64	>64	>64	>64
**34**	>64	>64	>64	>64	>64	>64
**35**	>64	>64	>64	>64	>64	>64
**36**	16	>64	>64	>64	>64	>64
**37**	>64	>64	>64	>64	>64	>64
**Fluconazole**	8	16	32	32	>64	64
**Amphotericin B**	>0.5	>0.5	>0.5	>0.5	>0.5	0.5

^1^. *C. albicans* AR-761, fluconazole-resistant isolate, USA origin. ^2^. *C. parapsilosis* AR-0339, *MDR1* overexpression. ^3^. *C. auris* AR-0381, East Asia clade. ^4^. *C. auris* AR-0382, South Asia clade. ^5^*. C. auris* AR-0383, African clade. ^6^. *C. auris* AR-0384, African clade.

**Table 3:** The in silico predicted physicochemical properties of selected antibacterial 3-((4-hydroxyphenyl)amino)propanoic acid derivatives.

Molecule	Molecular Weight	No. Heavy Atoms	No. Aromatic Heavy Atoms	Fraction Csp^3^	No. Rotatable Bonds	No. H Bond Acceptors	No. H Bond Donors
**9**	317.77	22	12	0.12	7	3	3
**13**	333.38	25	16	0.1	7	3	3
**14**	289.35	20	11	0.14	7	3	3
**15**	334.35	23	11	0.14	8	5	3
**16**	318.28	23	11	0.14	8	6	3
**20**	365.43	24	16	0.29	7	5	3
**21**	437.45	32	16	0.18	13	7	3
**22**	469.58	32	16	0.18	13	5	3
**24**	527.44	38	16	0.18	15	11	3
**29**	457.52	34	18	0.15	13	5	3
**30**	547.52	40	18	0.15	15	9	3
**31**	526.41	36	18	0.15	13	5	3
**33**	489.52	36	18	0.15	13	7	5
**36**	409.48	30	16	0.36	9	5	1
**37**	437.53	32	16	0.33	11	3	3
**Ceftazidime**	454.51	29	10	0.43	8	9	2

**Table 4:** The in silico predicted excretion and metabolism parameters of selected antibacterial 3-((4-hydroxyphenyl)amino)propanoic acid derivatives.

Molecule	Lipophilicity	GI Absorption	BBB Permeant	P-gp Substrate	CYP1A2 Inhibitor	CYP2C19 Inhibitor	CYP2C9 Inhibitor	CYP2D6 Inhibitor	CYP3A4 Inhibitor
**9**	2.85	High	Yes	No	Yes	Yes	Yes	Yes	No
**13**	3.26	High	Yes	No	Yes	Yes	Yes	Yes	No
**14**	2.27	High	No	No	Yes	No	No	No	No
**15**	1.55	Low	No	No	Yes	No	No	No	No
**16**	1.07	High	No	No	No	No	No	No	No
**20**	2.36	Low	No	No	No	No	No	No	Yes
**21**	1.96	High	No	Yes	No	No	Yes	Yes	Yes
**22**	3.27	Low	No	No	No	Yes	Yes	Yes	Yes
**24**	0.95	Low	No	Yes	No	Yes	Yes	No	Yes
**29**	3.24	High	No	No	No	Yes	Yes	Yes	No
**30**	1.86	Low	No	No	No	Yes	Yes	No	No
**31**	4.23	High	No	No	No	Yes	Yes	Yes	No
**33**	2.42	Low	No	No	No	No	Yes	No	No
**36**	2.74	High	No	No	No	Yes	Yes	No	Yes
**37**	2.82	High	No	Yes	No	Yes	No	Yes	Yes
**Ceftazidime**	−0.15	Low	No	Yes	No	No	No	No	No

**Table 5:** The in silico predicted excretion and metabolism parameters of selected antifungal 3-((4-hydroxyphenyl)amino)propanoic acid derivatives.

Molecule	Lipophilicity	GI Absorption	BBB Permeant	P-Gp Substrate	CYP1A2 Inhibitor	CYP2C19 Inhibitor	CYP2C9 Inhibitor	CYP2D6 Inhibitor	CYP3A4 Inhibitor
**14**	2.27	High	No	No	Yes	No	No	No	No
**16**	1.07	High	No	No	No	No	No	No	No
**17**	2.03	High	Yes	No	Yes	No	No	Yes	No
**FLU**	0.88	High	No	Yes	No	Yes	No	No	No

**Table 6:** The in silico predicted physicochemical properties of selected antifungal 3-((4-hydroxyphenyl)amino)propanoic acid derivatives.

Molecule	MW	No. Heavy Atoms	No. Aromatic Heavy Atoms	Fraction Csp3	No. Rotatable Bonds	No. H Bond Acceptors	No. H Bond Donors
**14**	289.35	20	11	0.14	7	3	3
**16**	318.28	23	11	0.14	8	6	3
**17**	273.33	20	11	0.27	6	2	3
**FLU**	306.27	22	16	0.23	5	7	1

## Data Availability

All data generated during this study are provided in the manuscript or Supplemental Materials.

## Supplementary Materials

The following supporting information can be downloaded at: https://www.mdpi.com/article/10.3390/antibiotics13020193/s1, Figure S1. 1H NMR spectrum of compound 56, Figure S2. 13C NMR spectrum of compound 56, Figure S3. 1H NMR spectrum of compound 67, Figure S4. 13C NMR spectrum of compound 67, Figure S5. 1H NMR spectrum of compound 78, Figure S6. 13C NMR spectrum of compound 78, Figure S7. 1H NMR spectrum of compound 89, Figure S8. 13C NMR spectrum of compound 89, Figure S9. 1H NMR spectrum of compound 910, Figure S10. 13C NMR spectrum of compound 910, Figure S11. 1H NMR spectrum of compound 101, Figure S12. 13C NMR spectrum of compound 101, Figure S13. 1H NMR spectrum of compound 112, Figure S14. 13C NMR spectrum of compound 112, Figure S15. 1H NMR spectrum of compound 123, Figure S16. 13C NMR spectrum of compound 123, Figure S17. 1H NMR spectrum of compound 134, Figure S18. 13C NMR spectrum of compound 134, Figure S19. 1H NMR spectrum of compound 145, Figure S20. 13C NMR spectrum of compound 145, Figure S21. 1H NMR spectrum of compound 156, Figure S22. 13C NMR spectrum of compound 156, Figure S23. 1H NMR spectrum of compound 167, Figure S24. 13C NMR spectrum of compound 167, Figure S25. 1H NMR spectrum of compound 178, Figure S26. 13C NMR spectrum of compound 178, Figure S27. 1H NMR spectrum of compound 189, Figure S28. 13C NMR spectrum of compound 189, Figure S29. 1H NMR spectrum of compound 1920, Figure S30. 13C NMR spectrum of compound 1920, Figure S31. 1H NMR spectrum of compound 201, Figure S32. 13C NMR spectrum of compound 201, Figure S33. 1H NMR spectrum of compound 212, Figure S34. 13C NMR spectrum of compound 212, Figure S35. 1H NMR spectrum of compound 223, Figure S36. 13C NMR spectrum of compound 223, Figure S37. 1H NMR spectrum of compound 234, Figure S38. 13C NMR spectrum of compound 234, Figure S39. 1H NMR spectrum of compound 245, Figure S40. 13C NMR spectrum of compound 245, Figure S41. 1H NMR spectrum of compound 256, Figure S42. 13C NMR spectrum of compound 256, Figure S43. 1H NMR spectrum of compound 267, Figure S44. 13C NMR spectrum of compound 267, Figure S45. 1H NMR spectrum of compound 278, Figure S46. 13C NMR spectrum of compound 278, Figure S47. 1H NMR spectrum of compound 289, Figure S48. 13C NMR spectrum of compound 289, Figure S49. 1H NMR spectrum of compound 2930, Figure S50. 13C NMR spectrum of compound 2930, Figure S51. 1H NMR spectrum of compound 301, Figure S52. 13C NMR spectrum of compound 301, Figure S53. 1H NMR spectrum of compound 312, Figure S54. 13C NMR spectrum of compound 312, Figure S55. 1H NMR spectrum of compound 323, Figure S56. 13C NMR spectrum of compound 323, Figure S57. 1H NMR spectrum of compound 334, Figure S58. 13C NMR spectrum of compound 334, Figure S59. 1H NMR spectrum of compound 345, Figure S60. 13C NMR spectrum of compound 345, Figure S61. 1H NMR spectrum of compound 356, Figure S62. 13C NMR spectrum of compound 356, Figure S63. 1H NMR spectrum of compound 367, Figure S64. 13C NMR spectrum of compound 367, Figure S65. 1H NMR spectrum of compound 37, Figure S66. 13C NMR spectrum of compound 37, and Figure S67. Mass spectra of compound 15.

## References

[1] Septimus E.J. (2018). Antimicrobial Resistance: An Antimicrobial/Diagnostic Stewardship and Infection Prevention Approach. Med. Clin. N. Am..

[2] Rice L.B. (2018). Antimicrobial Stewardship and Antimicrobial Resistance. Med. Clin. N. Am..

[3] De Oliveira D.M.P., Forde B.M., Kidd T.J., Harris P.N.A., Schembri M.A., Beatson S.A., Paterson D.L., Walker M.J. (2020). Antimicrobial Resistance in ESKAPE Pathogens. Clin. Microbiol. Rev..

[4] Mulani M.S., Kamble E.E., Kumkar S.N., Tawre M.S., Pardesi K.R. (2019). Emerging Strategies to Combat ESKAPE Pathogens in the Era of Antimicrobial Resistance: A Review. Front. Microbiol..

[5] Roch M., Sierra R., Andrey D.O. (2023). Antibiotic heteroresistance in ESKAPE pathogens, from bench to bedside. Clin. Microbiol. Infect..

[6] Du H., Bing J., Hu T., Ennis C.L., Nobile C.J., Huang G. (2020). Candida auris: Epidemiology, biology, antifungal resistance, and virulence. PLoS Pathog..

[7] Pristov K.E., Ghannoum M.A. (2019). Resistance of Candida to azoles and echinocandins worldwide. Clin. Microbiol. Infect..

[8] Nowak M.G., Skwarecki A.S., Milewska M.J. (2021). Amino Acid Based Antimicrobial Agents—Synthesis and Properties. ChemMedChem.

[9] Li X., Meng X., Duan H., Wang L., Wang S., Zhang Y., Qin D. (2010). Original and efficient synthesis of D-cycloserine. Arch. Pharm..

[10] Kotnik M., Humljan J., Contreras-Martel C., Oblak M., Kristan K., Hervé M., Blanot D., Urleb U., Gobec S., Dessen A. (2007). Structural and functional characterization of enantiomeric glutamic acid derivatives as potential transition state analogue inhibitors of MurD ligase. J. Mol. Biol..

[11] Humljan J., Kotnik M., Contreras-Martel C., Blanot D., Urleb U., Dessen A., Solmajer T., Gobec S. (2008). Novel naphthalene-N-sulfonyl-D-glutamic acid derivatives as inhibitors of MurD, a key peptidoglycan biosynthesis enzyme. J. Med. Chem..

[12] Ellsworth B.A., Tom N.J., Bartlett P.A. (1996). Synthesis and evaluation of inhibitors of bacterial D-alanine:D-alanine ligases. Chem. Biol..

[13] Caselli E., Powers R.A., Blasczcak L.C., Wu C.Y., Prati F., Shoichet B.K. (2001). Energetic, structural, and antimicrobial analyses of beta-lactam side chain recognition by beta-lactamases. Chem. Biol..

[14] Wamp S., Rothe P., Stern D., Holland G., Döhling J., Halbedel S. (2022). MurA escape mutations uncouple peptidoglycan biosynthesis from PrkA signaling. PLoS Pathog..

[15] Hummels K.R., Berry S.P., Li Z., Taguchi A., Min J.K., Walker S., Marks D.S., Bernhardt T.G. (2023). Coordination of bacterial cell wall and outer membrane biosynthesis. Nature.

[16] Weng C.J., Yen G.C. (2012). Chemopreventive effects of dietary phytochemicals against cancer invasion and metastasis: Phenolic acids, monophenol, polyphenol, and their derivatives. Cancer Treat. Rev..

[17] Zhang B., Zhang Y., Liu X., Zhao C., Yin J., Li X., Zhang X., Wang J., Wang S. (2023). Distinctive anti-inflammatory effects of resveratrol, dihydroresveratrol, and 3-(4-hydroxyphenyl)-propionic acid on DSS-induced colitis in pseudo-germ-free mice. Food Chem..

[18] Bertašiūtė M., Kavaliauskas P., Vaickelionienė R., Grybaitė B., Petraitis V., Petraitienė R., Naing E., Garcia A., Šiugždaitė J., Lelešius R. (2023). Synthesis of 1-(2-Hydroxyphenyl)- and (3,5-Dichloro-2-hydroxyphenyl)-5-oxopyrrolidine-3-carboxylic Acid Derivatives as Promising Scaffolds for the Development of Novel Antimicrobial and Anticancer Agents. Int. J. Mol. Sci..

[19] (1989). Phenol. IARC Monogr. Eval. Carcinog. Risks Hum..

[20] Scott K.A., Cox P.B., Njardarson J.T. (2022). Phenols in Pharmaceuticals: Analysis of a Recurring Motif. J. Med. Chem..

[21] Pinheiro P.F., Menini L.A.P., Bernardes P.C., Saraiva S.H., Carneiro J.W.M., Costa A.V., Arruda T.R., Lage M.R., Gonçalves P.M., Bernardes C.O. (2018). Semisynthetic Phenol Derivatives Obtained from Natural Phenols: Antimicrobial Activity and Molecular Properties. J. Agric. Food Chem..

[22] Baltrusis R.S., Beresnevičius Z.J., Mickevičius V. (1982). Synthesis and transformations of N-(4-hydroxyphenyl)dihydrouracils. Chem. Heterocycl. Compd..

[23] Beattie J.K., McErlean C.S.P., Phippen C.B.W. (2010). On-water conjugate additions of anilines. Chem. Commun..

[24] Tumosienė I., Jakienė E., Kantminienė K., Rutkauskas K., Beresnevičius Z.J. (2010). Synthesis and plant growth regulating activity of halo derivatives of 3,3′-(arylimino)dipropanoic acids. Chemija.

[25] Anusevičius K., Mickevičius V., Mikulskienė G. (2010). Synthesis and structure of *N*-(4-bromophenyl)-N-carboxyethyl-*β*-alanine derivatives. Chemija.

[26] Tumosienė I., Mikulskienė G., Kantminienė K., Beresnevičius Z.J. (2011). Synthesis and structure of 3,3′-[(4-alkoxyphenyl)imino]bis(N’-phthaloyl- or N’-benzylidenepropanohydrazide) derivatives. Chemija.

[27] Paulusma C.C., Lamers W.H., Broer S., van de Graaf S.F.J. (2022). Amino acid metabolism, transport and signalling in the liver revisited. Biochem. Pharmacol..

[28] Ngo D.H., Vo T.S. (2019). An Updated Review on Pharmaceutical Properties of Gamma-Aminobutyric Acid. Molecules.

[29] Koksharova O.A., Safronova N.A. (2022). Non-Proteinogenic Amino Acid β-N-Methylamino-L-Alanine (BMAA): Bioactivity and Ecological Significance. Toxins.

[30] López-López L.I., Rivera-Ávalos E., Villarreal-Reyes C., Martínez-Gutiérrez F., de Loera D. (2022). Synthesis and Antimicrobial Evaluation of Amino Acid Naphthoquinone Derivatives as Potential Antibacterial Agents. Chemotherapy.

[31] Sajjad F., Sun N.N., Chen T., Yan Y.J., Margetić D., Chen Z.L. (2021). Evaluation of antimicrobial photodynamic activities of 5-aminolevulinic acid derivatives. Photodermatol. Photoimmunol. Photomed..

[32] Skwarecki A.S., Nowak M.G., Milewska M.J. (2021). Amino Acid and Peptide-Based Antiviral Agents. ChemMedChem.

[33] Cui J., Ji X., Mi Y., Miao Q., Dong F., Tan W., Guo Z. (2022). Antimicrobial and Antioxidant Activities of N-2-Hydroxypropyltrimethyl Ammonium Chitosan Derivatives Bearing Amino Acid Schiff Bases. Mar. Drugs.

[34] Chang R.Y.K., Nang S.C., Chan H.K., Li J. (2022). Novel antimicrobial agents for combating antibiotic-resistant bacteria. Adv. Drug Deliv. Rev..

[35] Loyola-Cruz M.Á., Gonzalez-Avila L.U., Martínez-Trejo A., Saldaña-Padilla A., Hernández-Cortez C., Bello-López J.M., Castro-Escarpulli G. (2023). ESKAPE and Beyond: The Burden of Coinfections in the COVID-19 Pandemic. Pathogens.

