# Supplementary material for: Synthesis of 3-((4-Hydroxyphenyl)amino)propanoic Acid Derivatives as Promising Scaffolds for the Development of Antimicrobial Candidates Targeting Multidrug-Resistant Bacterial and Fungal Pathogens
Source: Antibiotics (Basel). 2024 Feb 17;13(2):193. doi: 10.3390/antibiotics13020193 (PMC10886201; doi:10.3390/antibiotics13020193)
Supplement: Supplementary file 1 [file antibiotics-13-00193-s001.zip › antibiotics-2836647-supplementary.pdf]

Synthesis of 3-((4-hydroxyphenyl)amino)propanoic Acid Derivatives as Promising Scaffolds for the Development of Antimicrobial Candidates Targeting Multidrug-Resistant Bacterial and Fungal Pathogens

Povilas Kavaliauskas [1,2,3,4,5,*], Birutė Grybaitė [1], Birutė Sapijanskaitė-Banevič [1], Rita Vaickelionienė [1], Vidmantas Petraitis [2,3,5], Rūta Petraitienė [3,5], Ethan Naing [3], Andrew Garcia [3], Ramunė Grigalevičiūtė [2,6] and Vytautas Mickevičius [1]

Supplementary Information

Content: Supplemental Figures S1–S66 representing ^{1}H and ^{13}C NMR spectra (in DMSO-*d*$_6$) of compounds 5−37 and S67 representing mass spectra of compounds 15

((4-Hydroxyphenyl)azanediyl)dimethyl ester ***(5)***

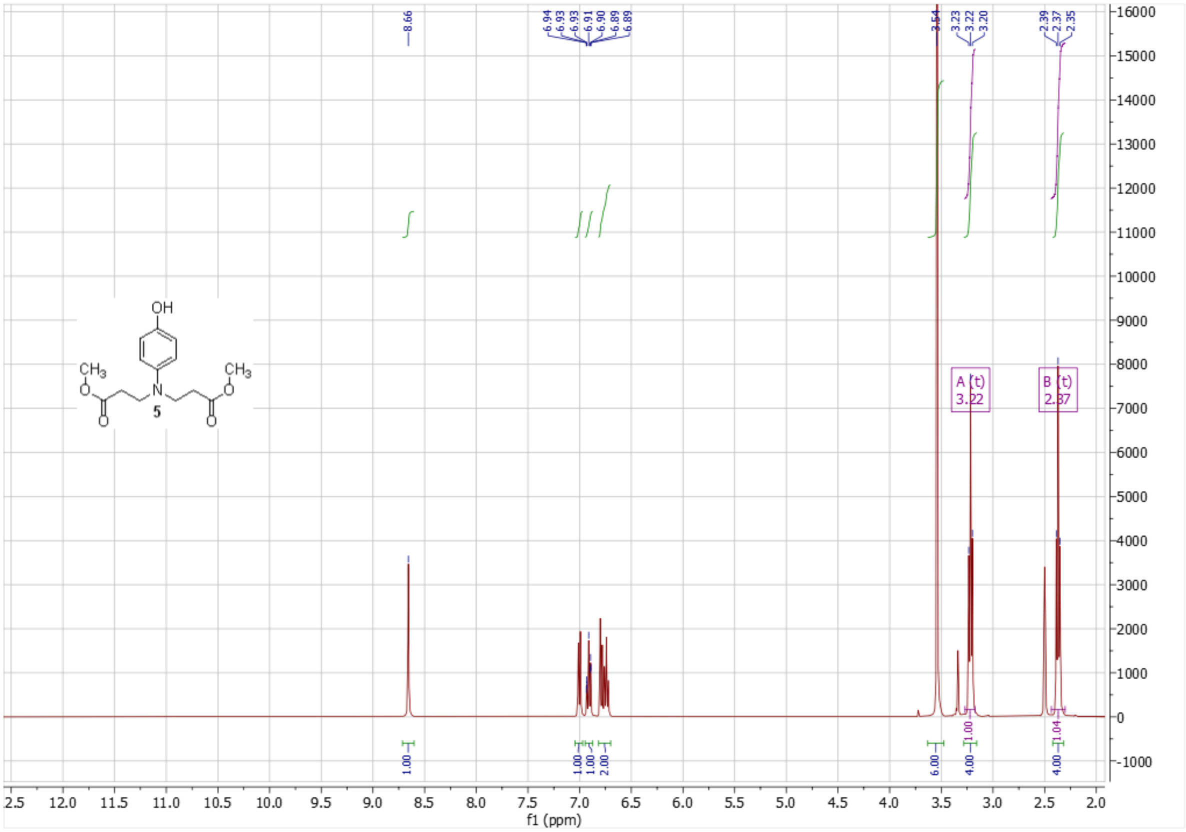

Figure S1. ^{1}H NMR spectrum of compound **5**

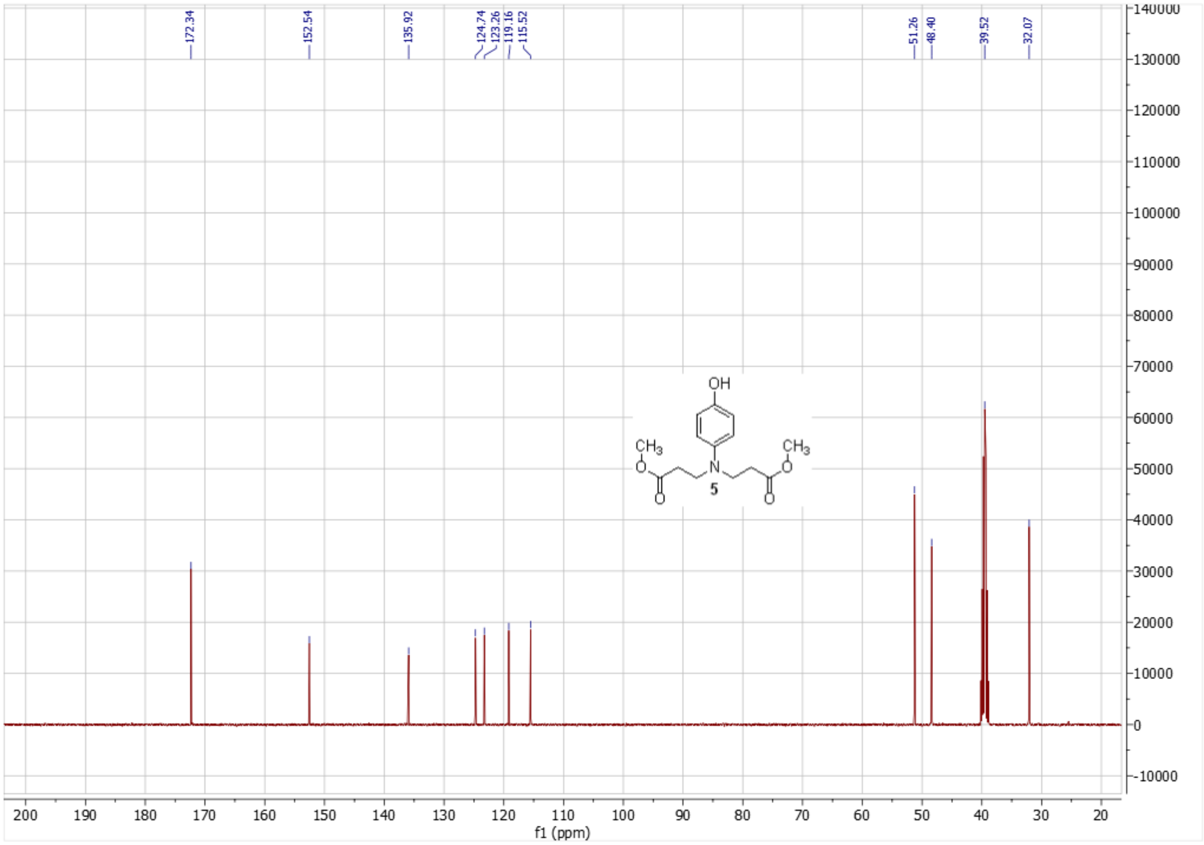

Figure S2. ^{13}C NMR spectrum of compound **5**

3,3'-((4-Hydroxyphenyl)azanediyl)di(propanehydrazide) ***(6)***

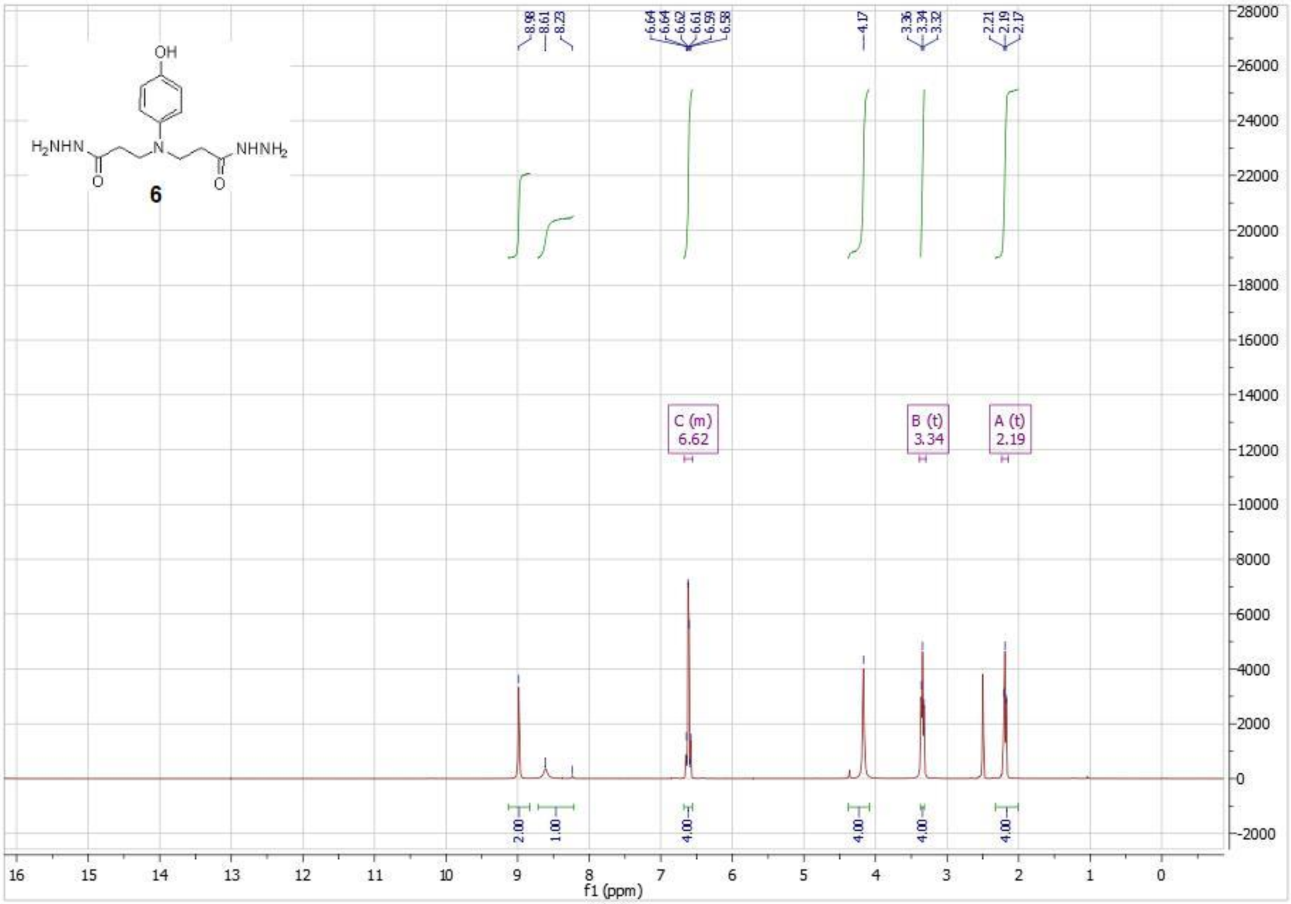

Figure S3. ^{1}H NMR spectrum of compound **6**

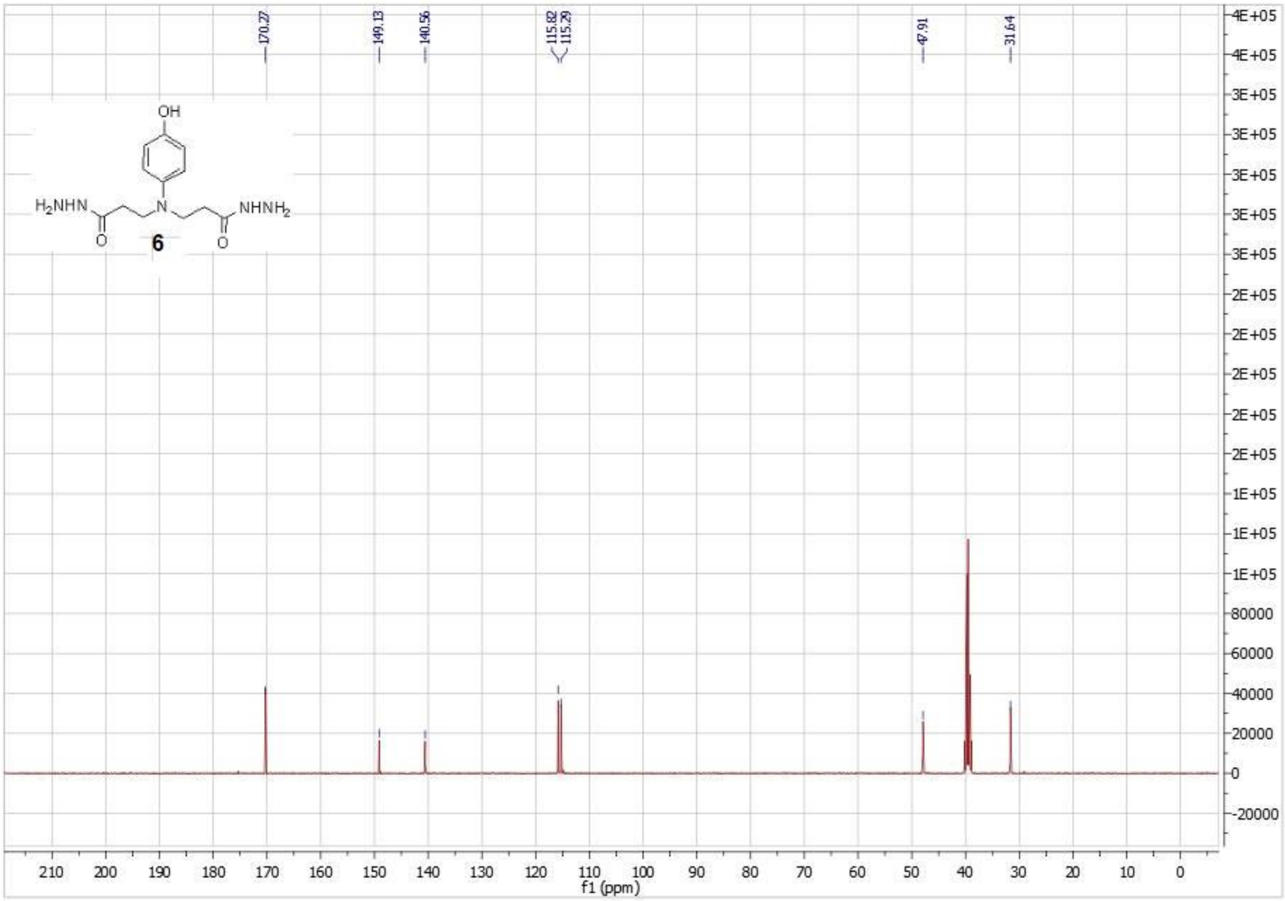

Figure S4. ^{13}C NMR spectrum of compound **6**

N'-benzylidene-3-((4-hydroxyphenyl)amino)propanehydrazide ***(7)***

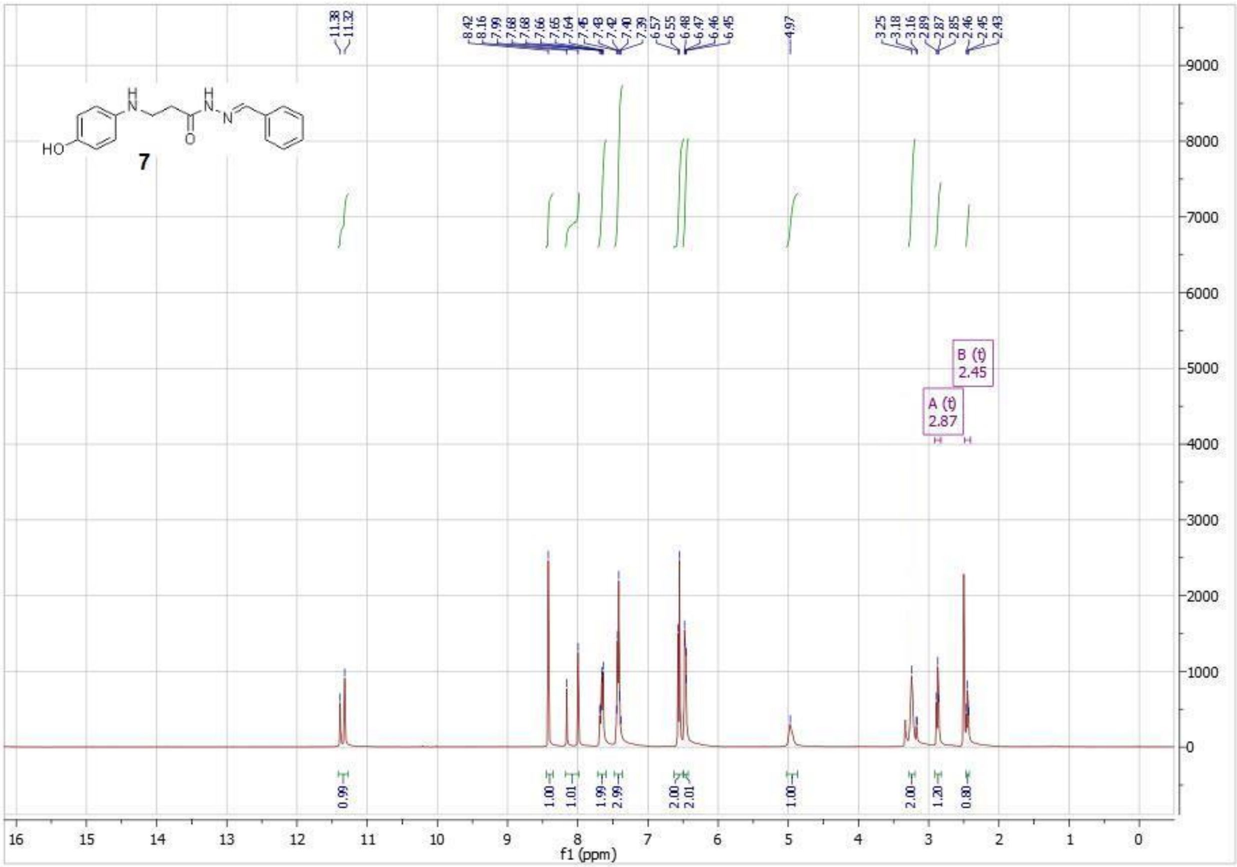

Figure S5. ^{1}H NMR spectrum of compound **7**

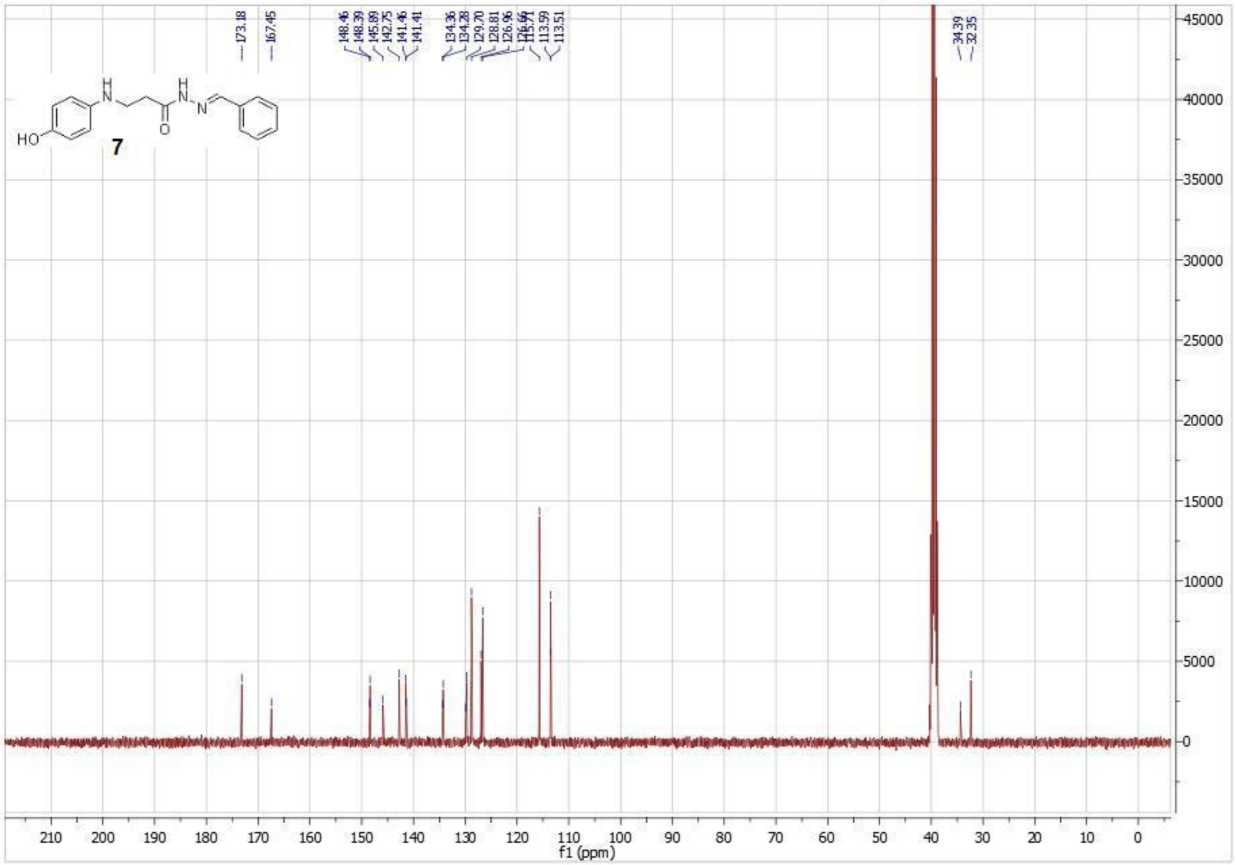

Figure S6. ^{13}C NMR spectrum of compound **7**

3-((4-Hydroxyphenyl)amino)-N'-(4-nitrobenzylidene)propanehydrazide ***(8)***

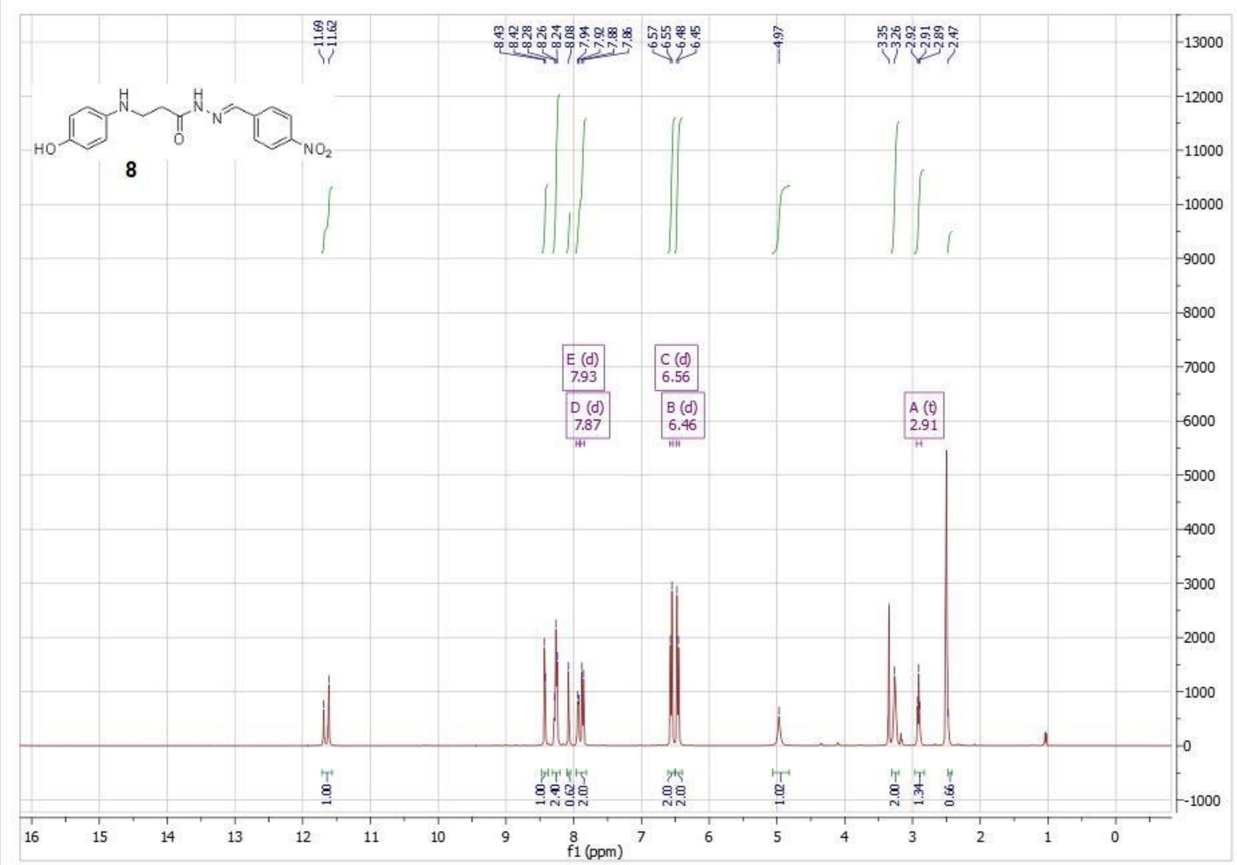

Figure S7. ^{1}H NMR spectrum of compound **8**

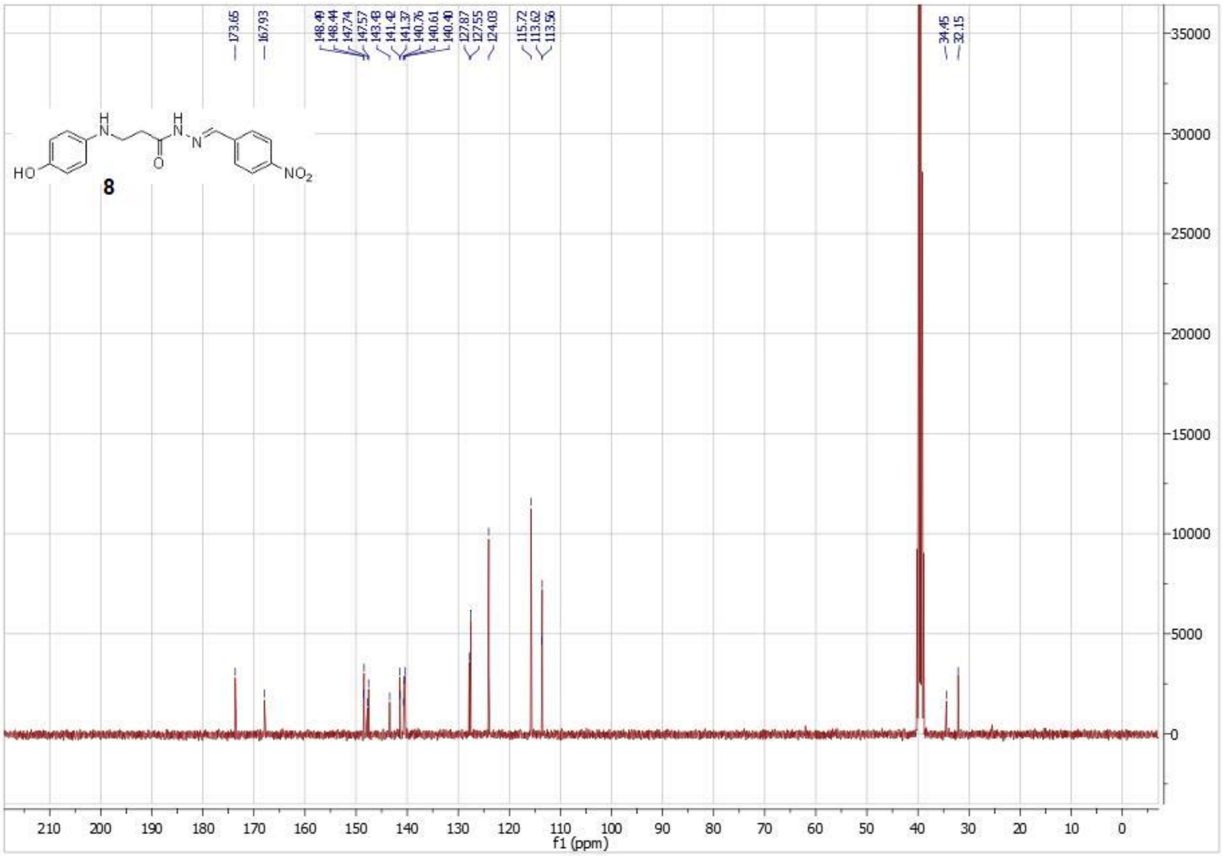

Figure S8. ^{13}C NMR spectrum of compound **8**

N'-(4-chlorobenzylidene)-3-((4-hydroxyphenyl)amino)propanehydrazide ***(9)***

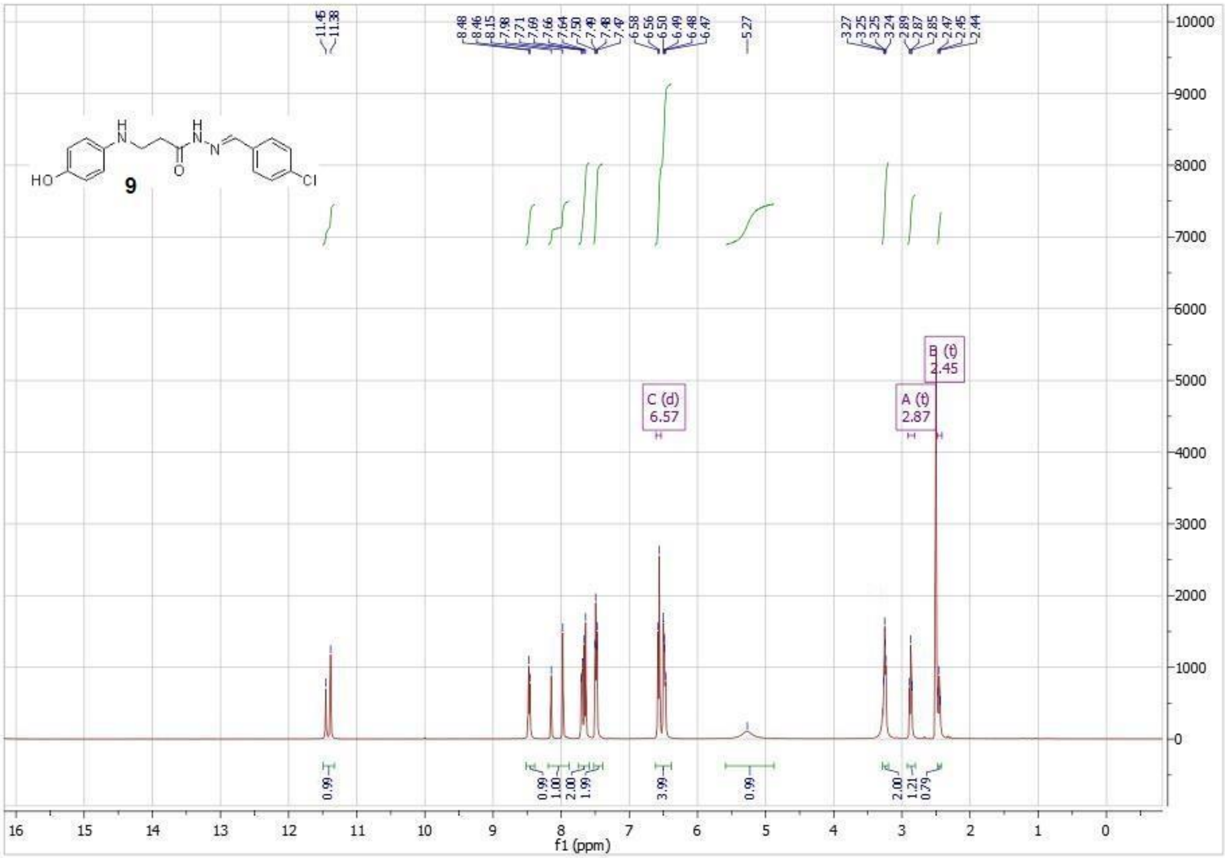

Figure S9. ^{1}H NMR spectrum of compound **9**

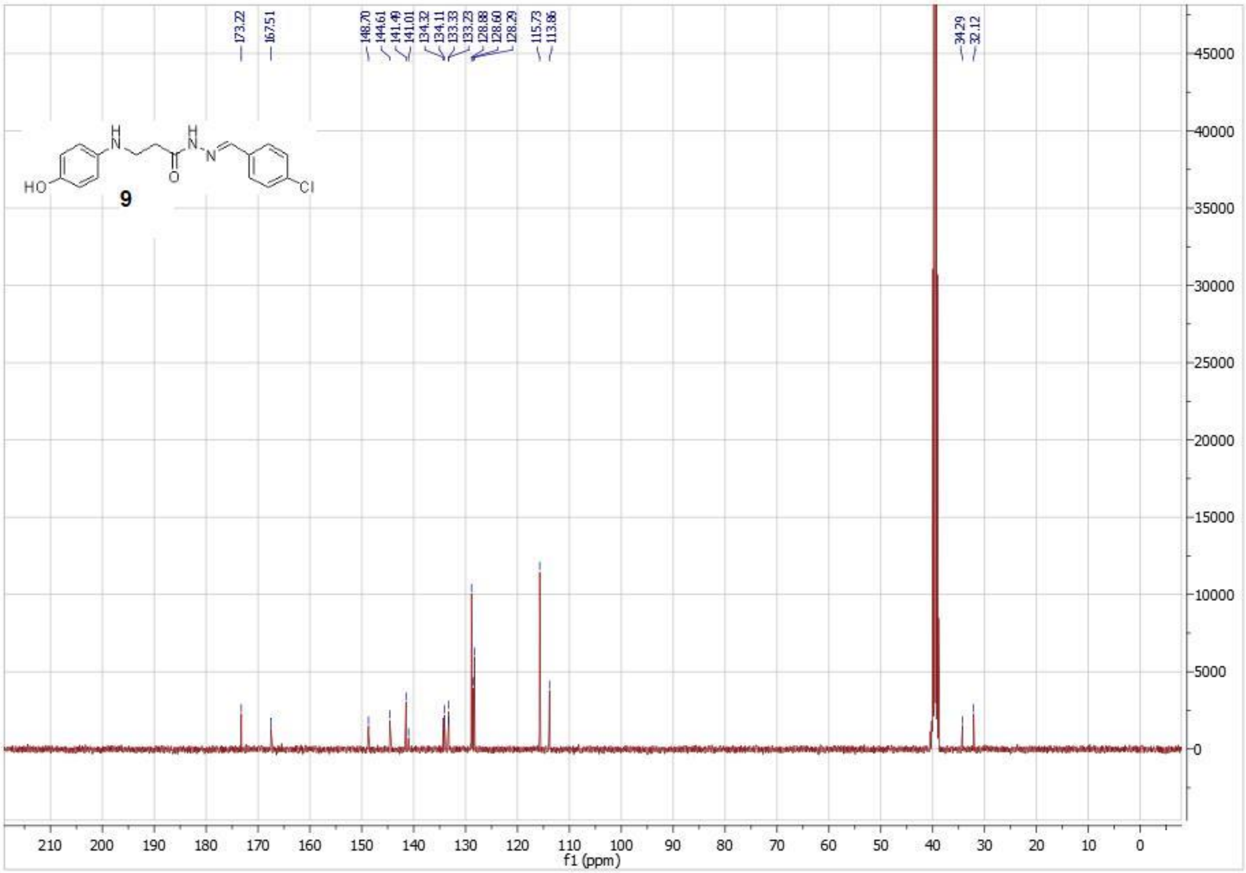

Figure S10. ^{13}C NMR spectrum of compound **9**

N'-(4-(dimethylamino)benzylidene)-3-((4-hydroxyphenyl)amino)propanehydrazide ***(10)***

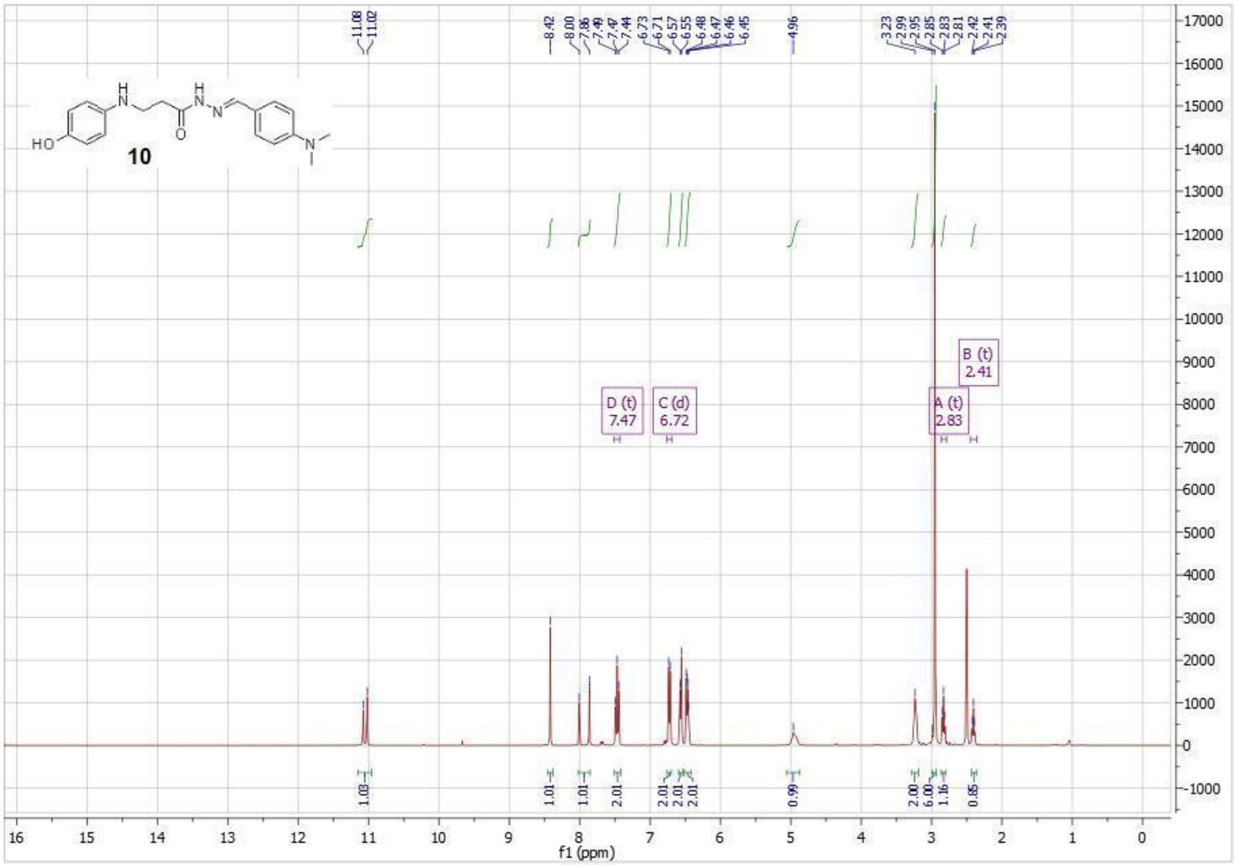

Figure S11. ^{1}H NMR spectrum of compound **10**

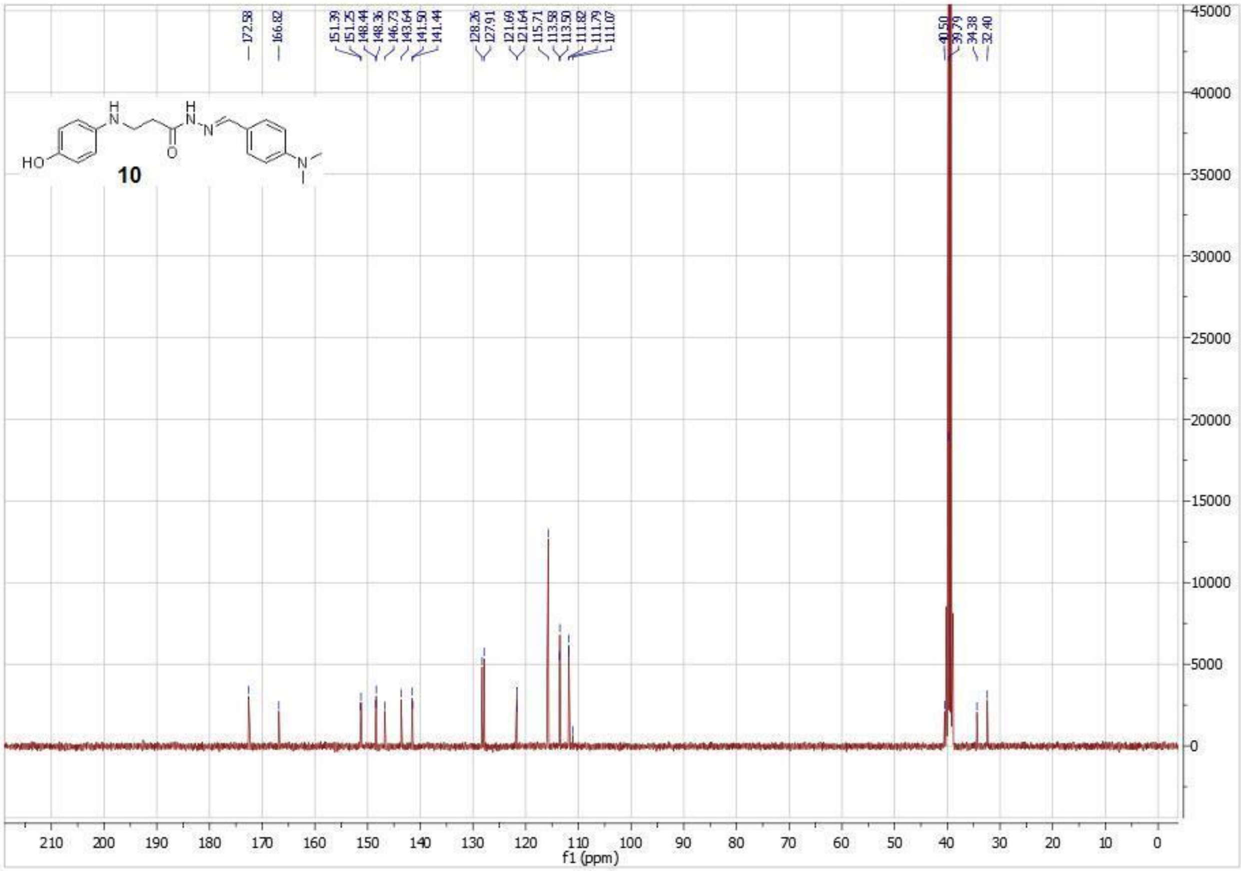

Figure S12. ^{13}C NMR spectrum of compound **10**

N'-(4-hydroxybenzylidene)-3-((4-hydroxyphenyl)amino)propanehydrazide ***(11)***

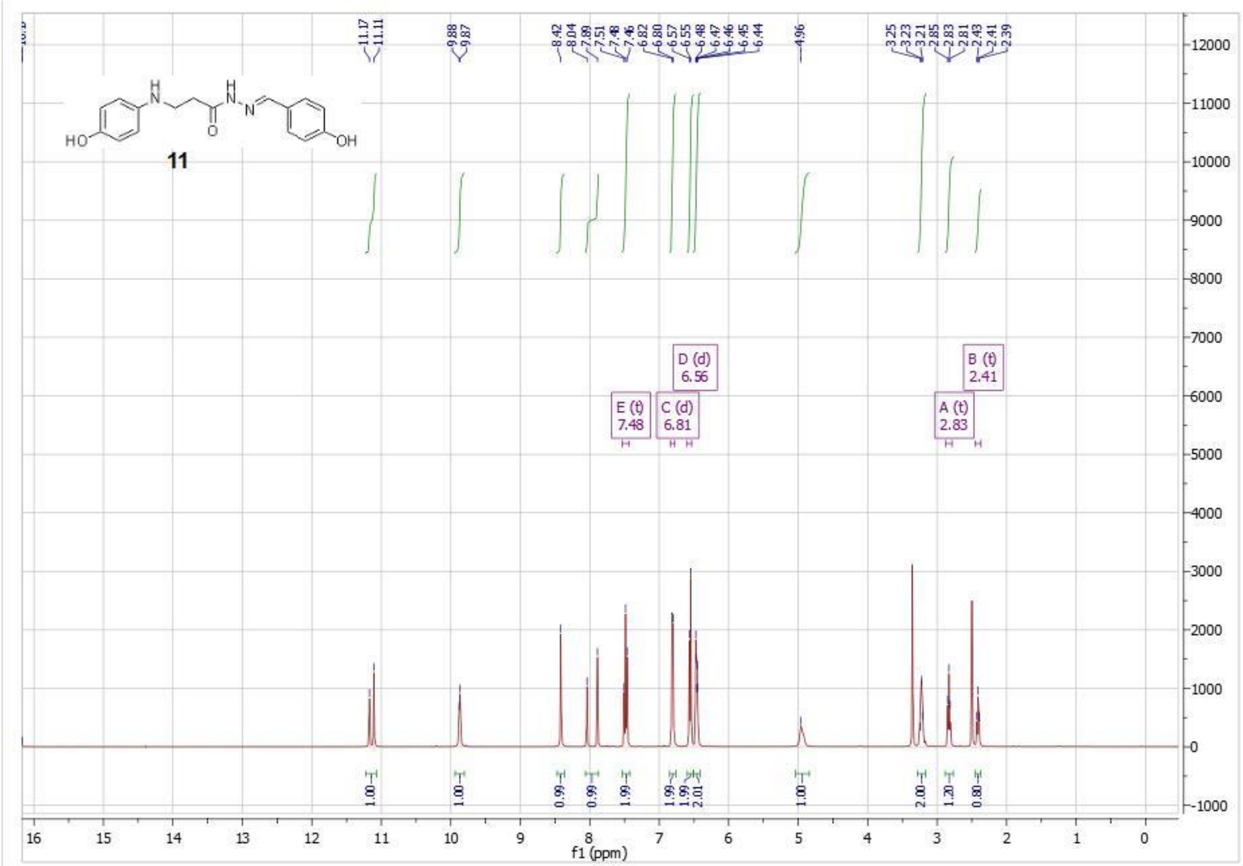

Figure S13. ^{1}H NMR spectrum of compound **11**

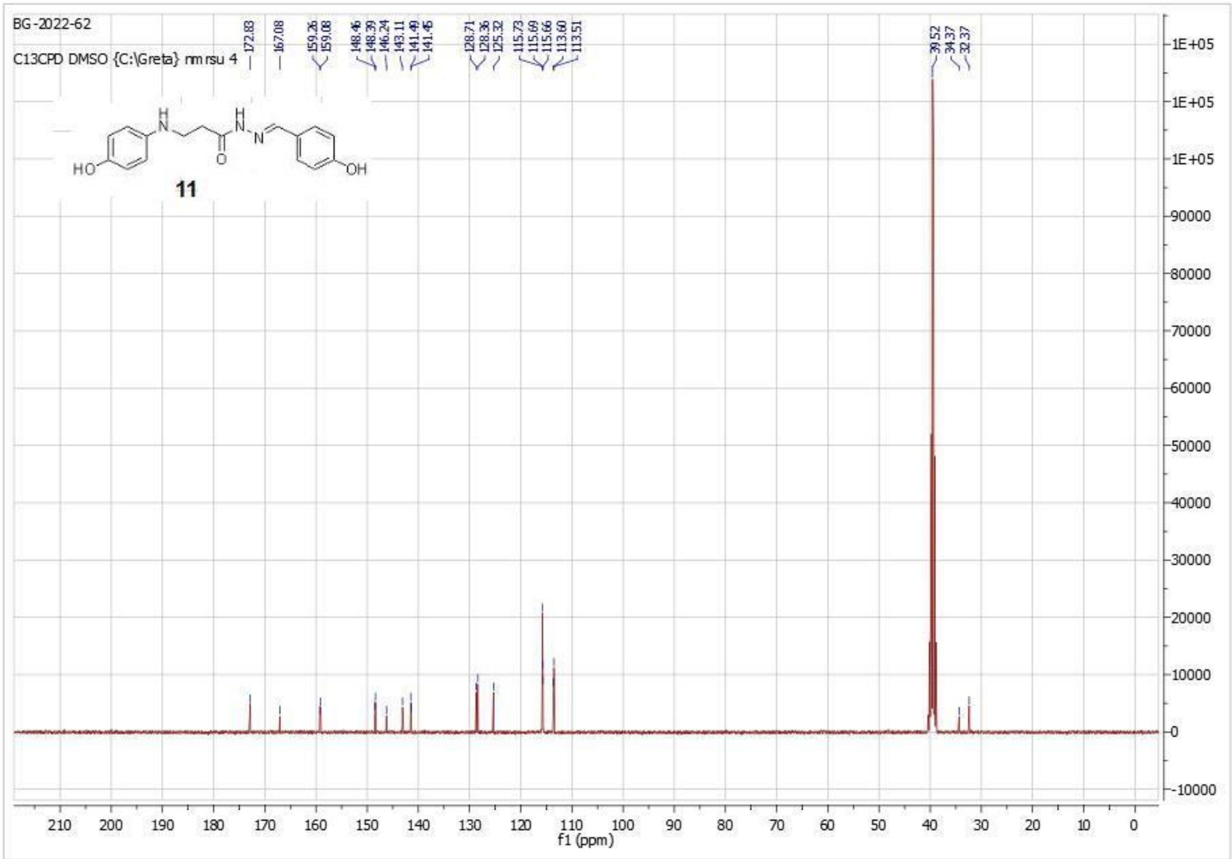

Figure S14. ^{13}C NMR spectrum of compound **11**

3-((4-Hydroxyphenyl)amino)-N'-(3,4,5-trimethoxybenzylidene)propanehydrazide ***(12)***

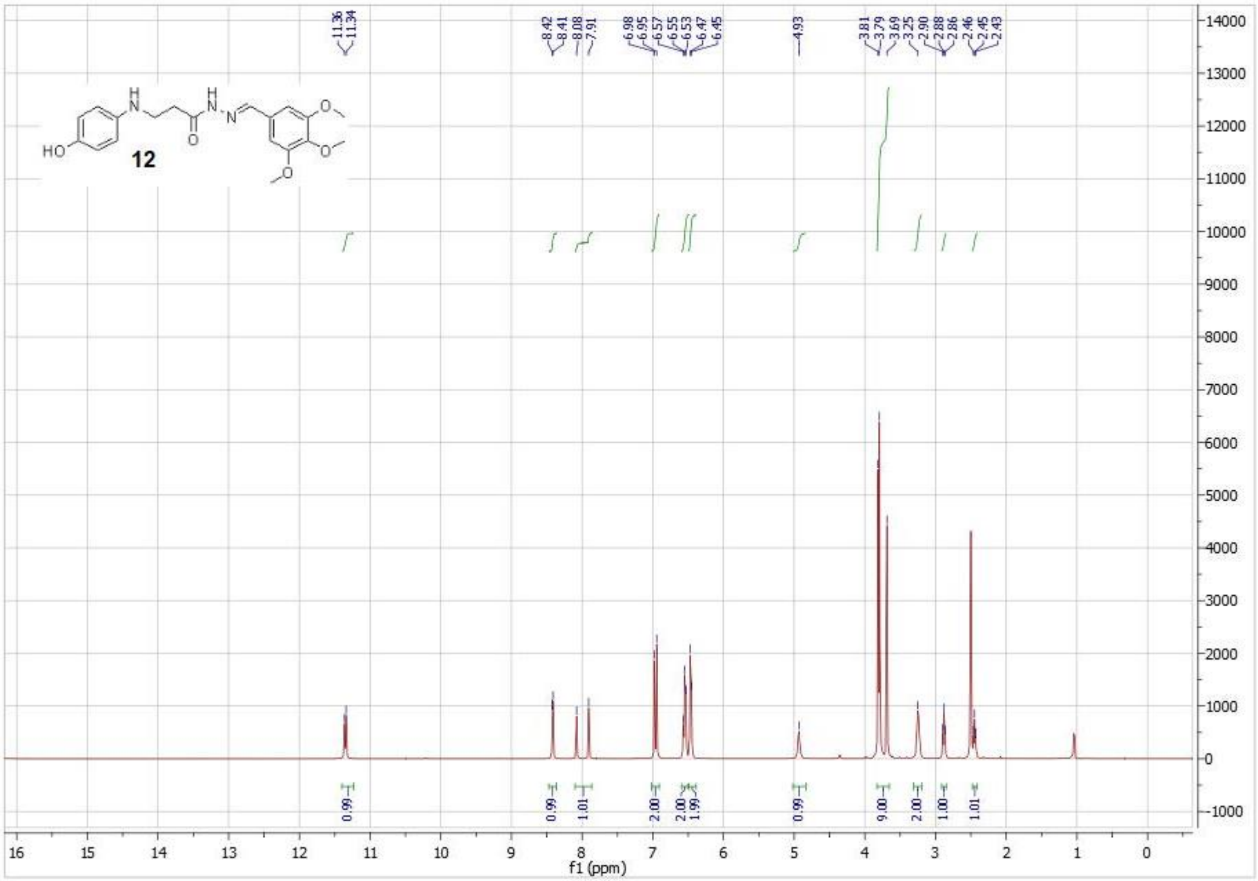

Figure S15. ^{1}H NMR spectrum of compound **12**

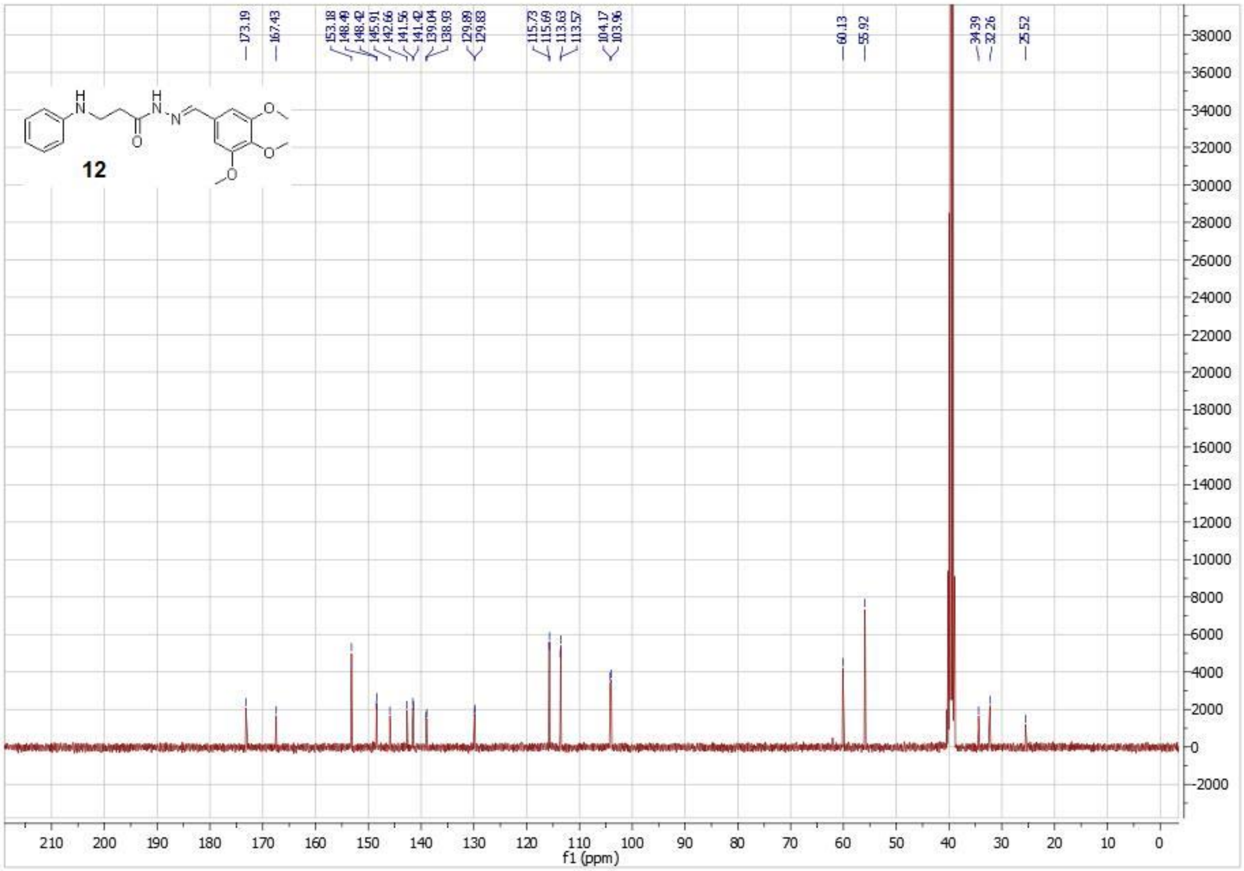

Figure S16. ^{13}C NMR spectrum of compound **12**

3-((4-hydroxyphenyl)amino)-N'-(naphthalen-1-ylmethylene)propanehydrazide ***(13)***

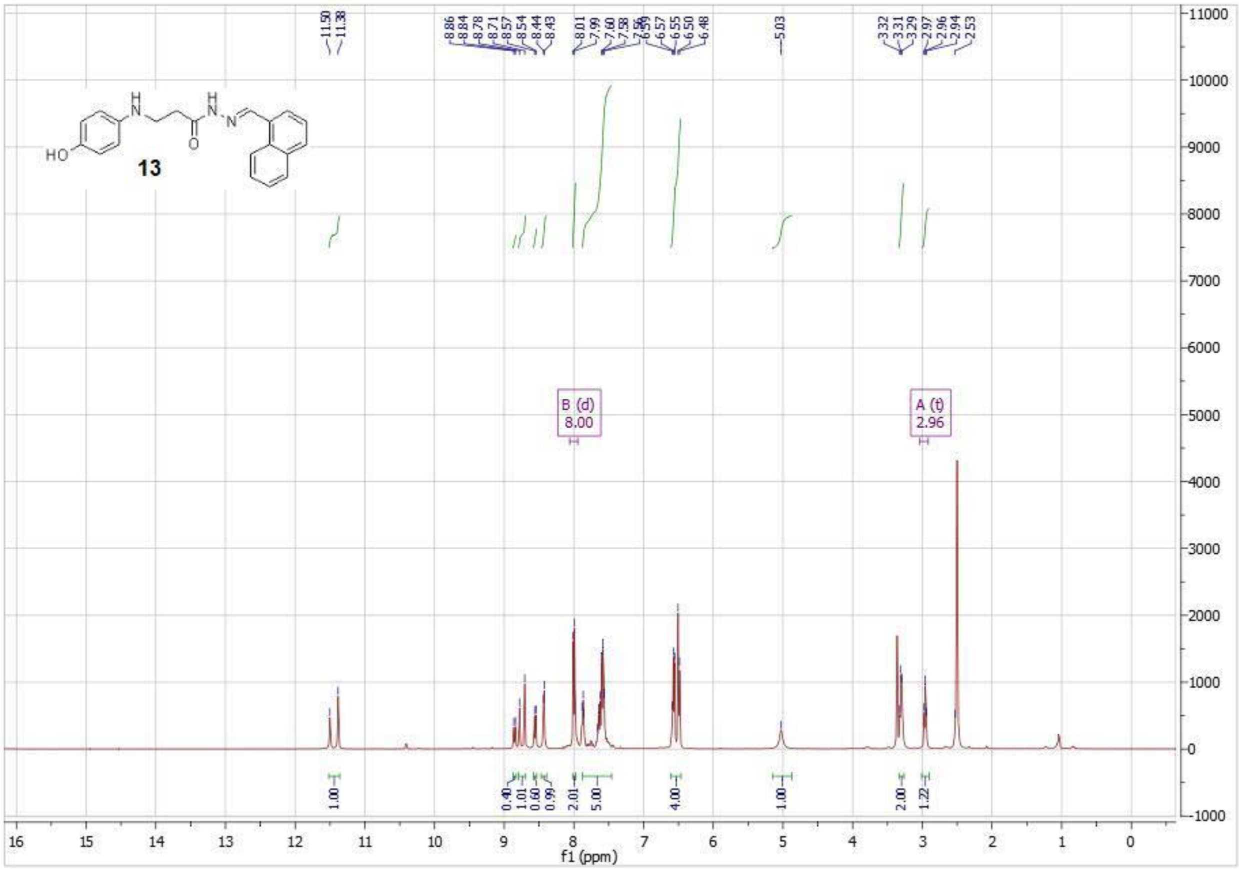

Figure S17. ^{1}H NMR spectrum of compound **13**

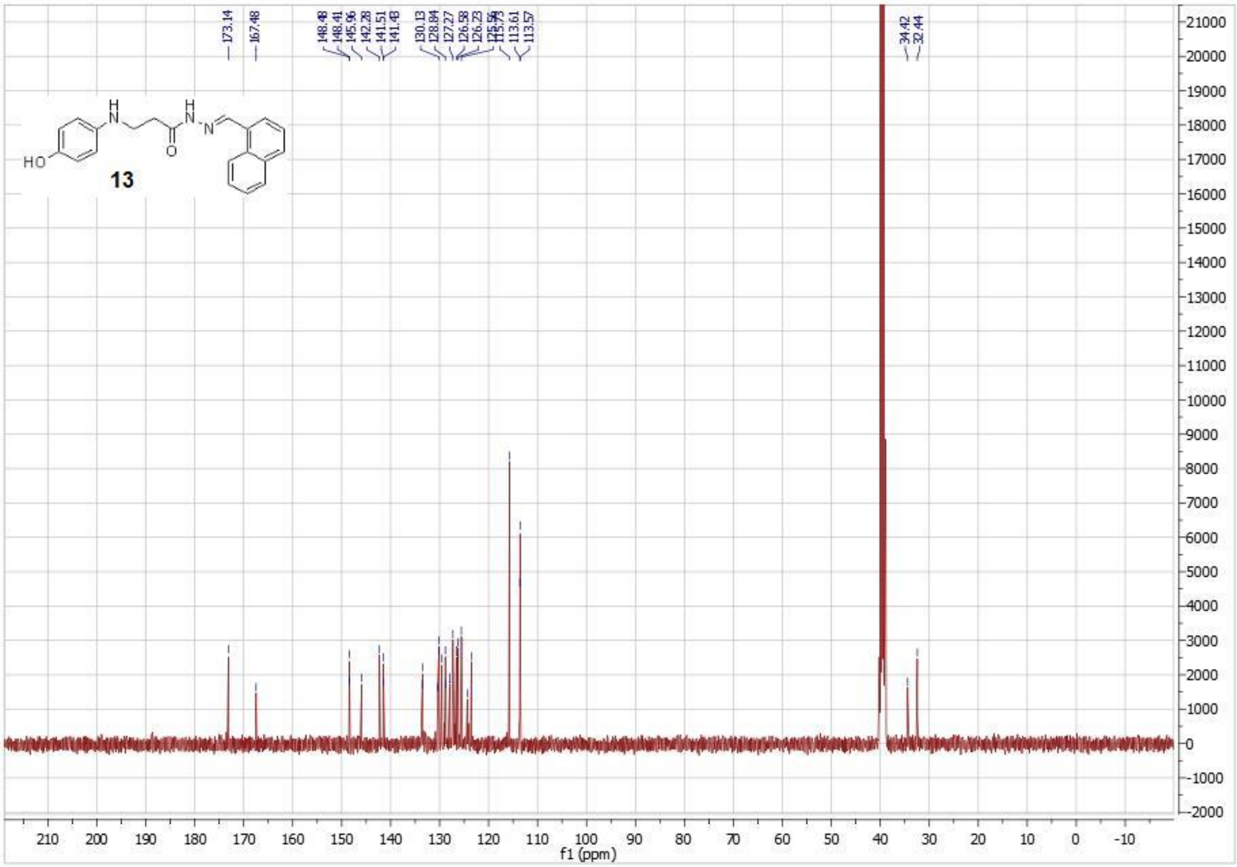

Figure S18. ^{13}C NMR spectrum of compound **13**

3-((4-Hydroxyphenyl)amino)-N'-(thien-2-ylmethylene)propanehydrazide ***(14)***

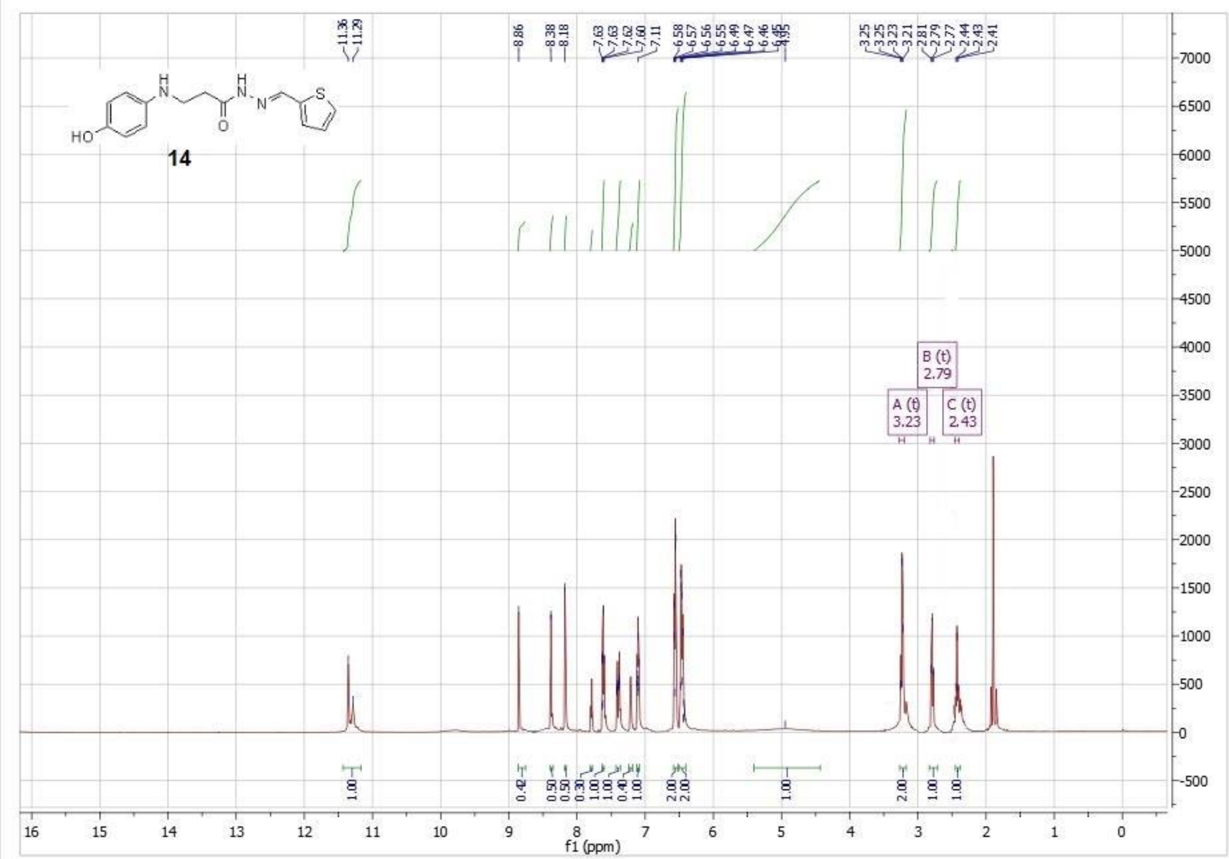

Figure S19. ^{1}H NMR spectrum of compound **14**

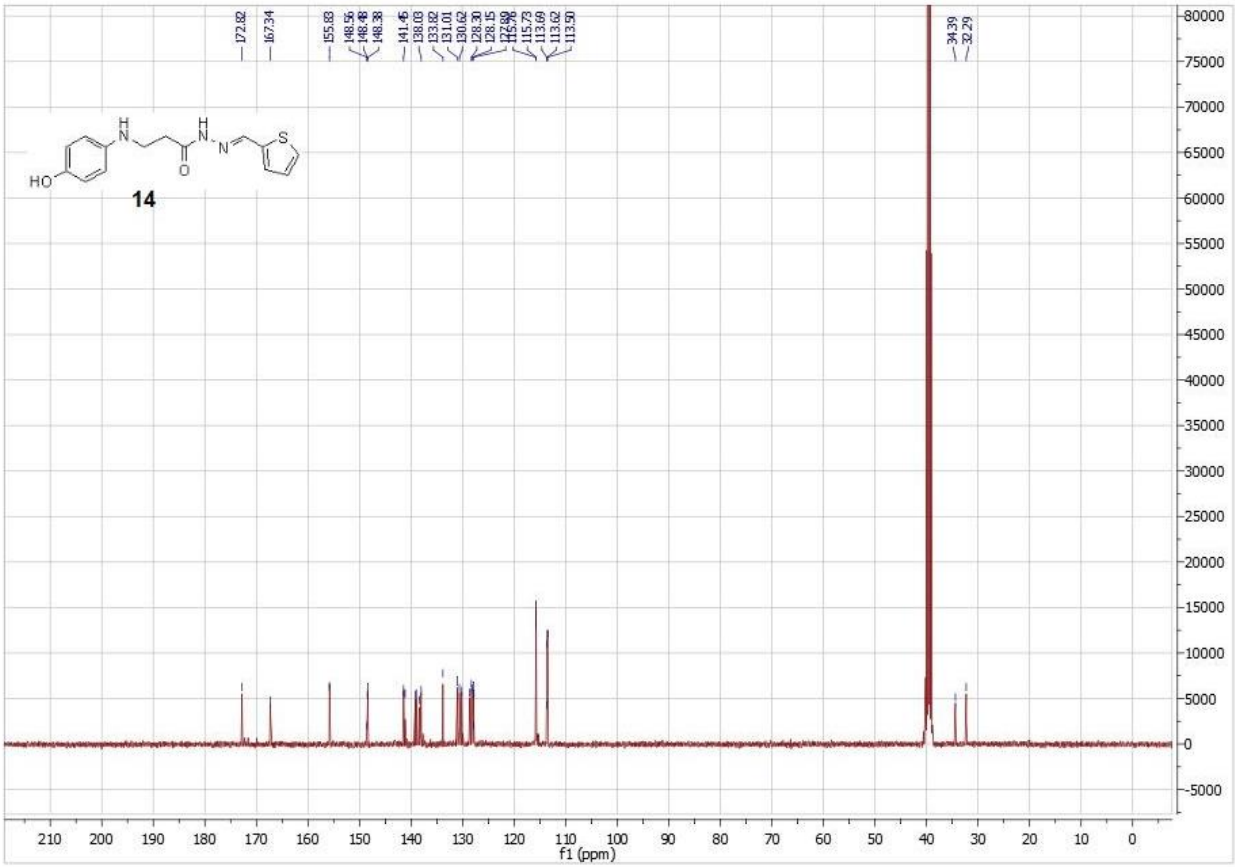

Figure S20. ^{13}C NMR spectrum of compound **14**

3-((4-Hydroxyphenyl)amino)-N'-((5-nitrothien-2-yl)methylene)propanehydrazide **(15)**

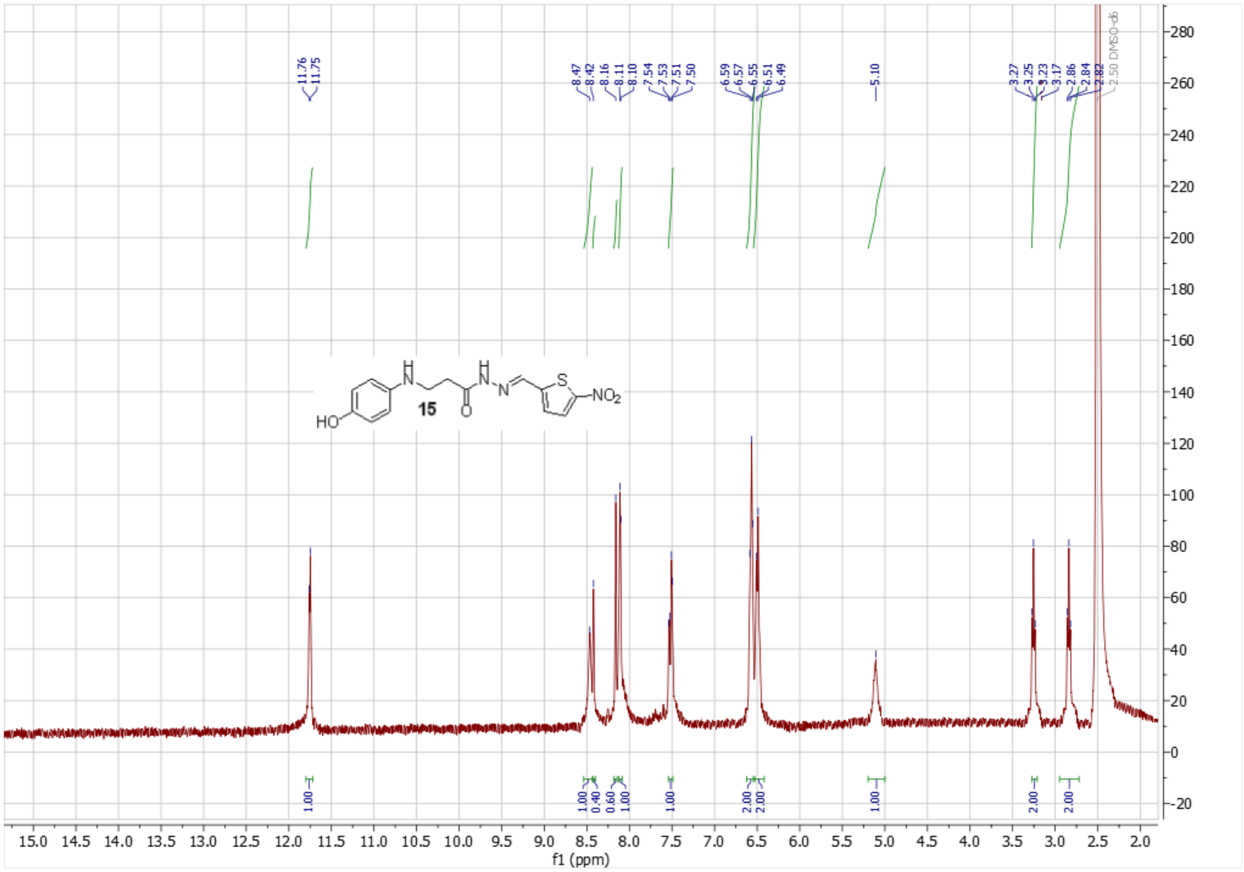

Figure S21. ^{1}H NMR spectrum of compound **15**

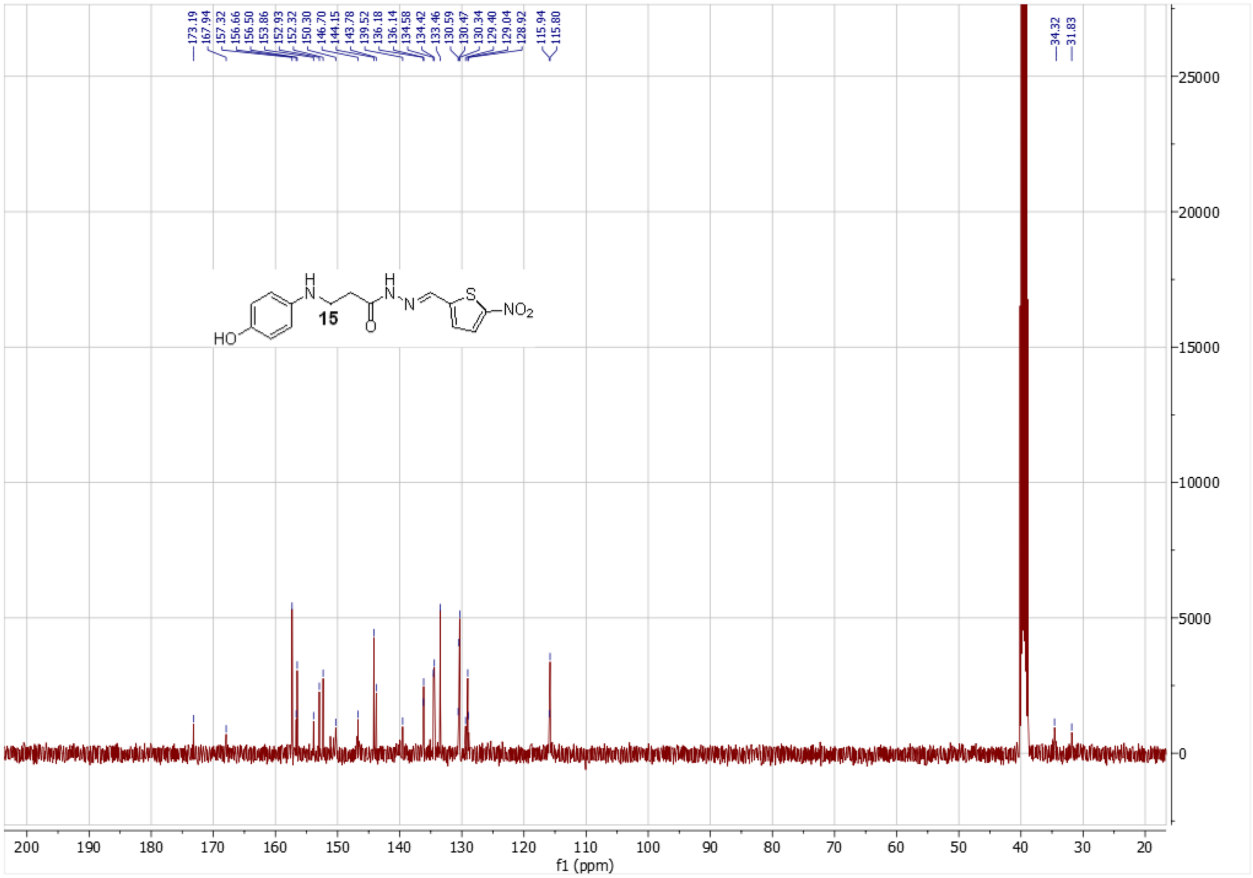

Figure S22. ^{13}C NMR spectrum of compound **15**

3-((4-Hydroxyphenyl)amino)-N'-((5-nitrofuryl-2-yl)methylene)propanehydrazide ***(16)***

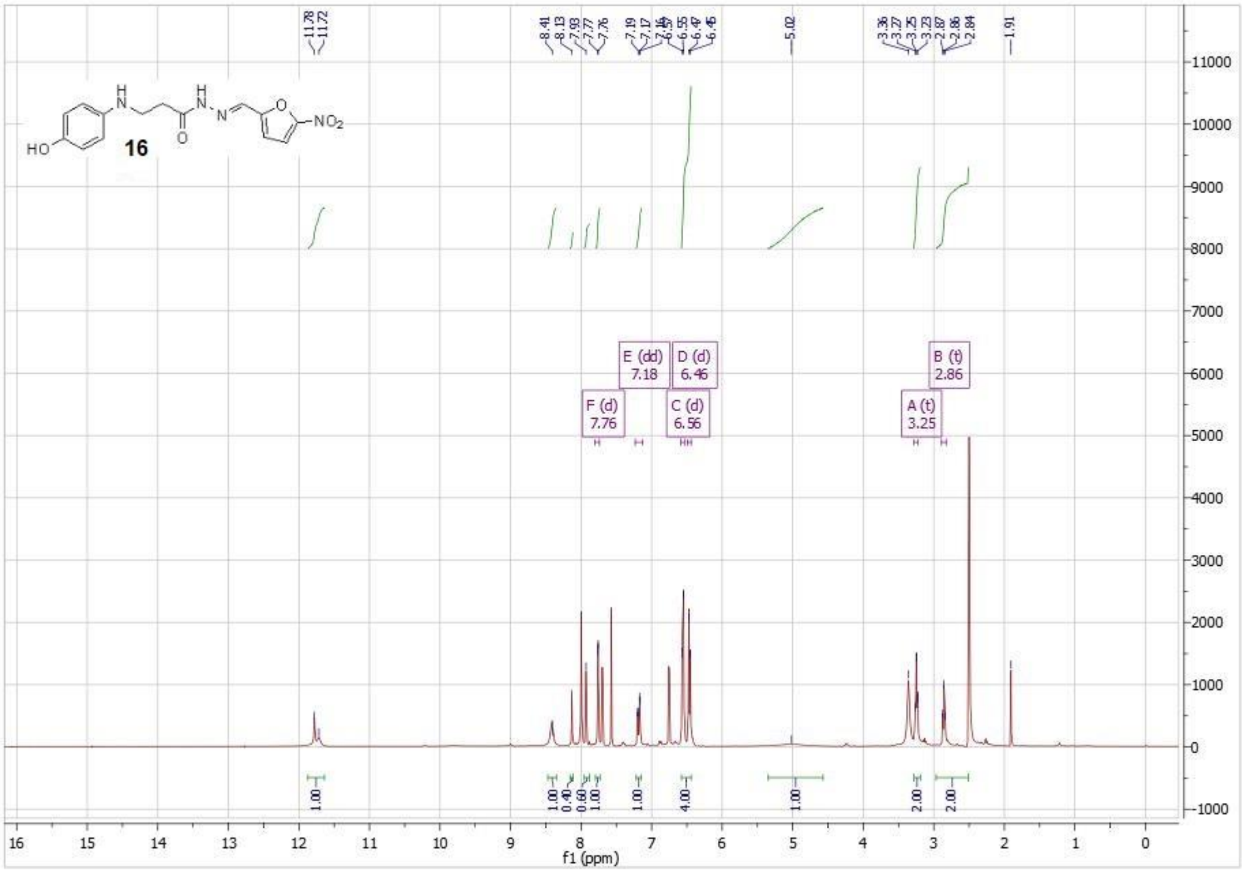

Figure S23. ^{1}H NMR spectrum of compound **16**

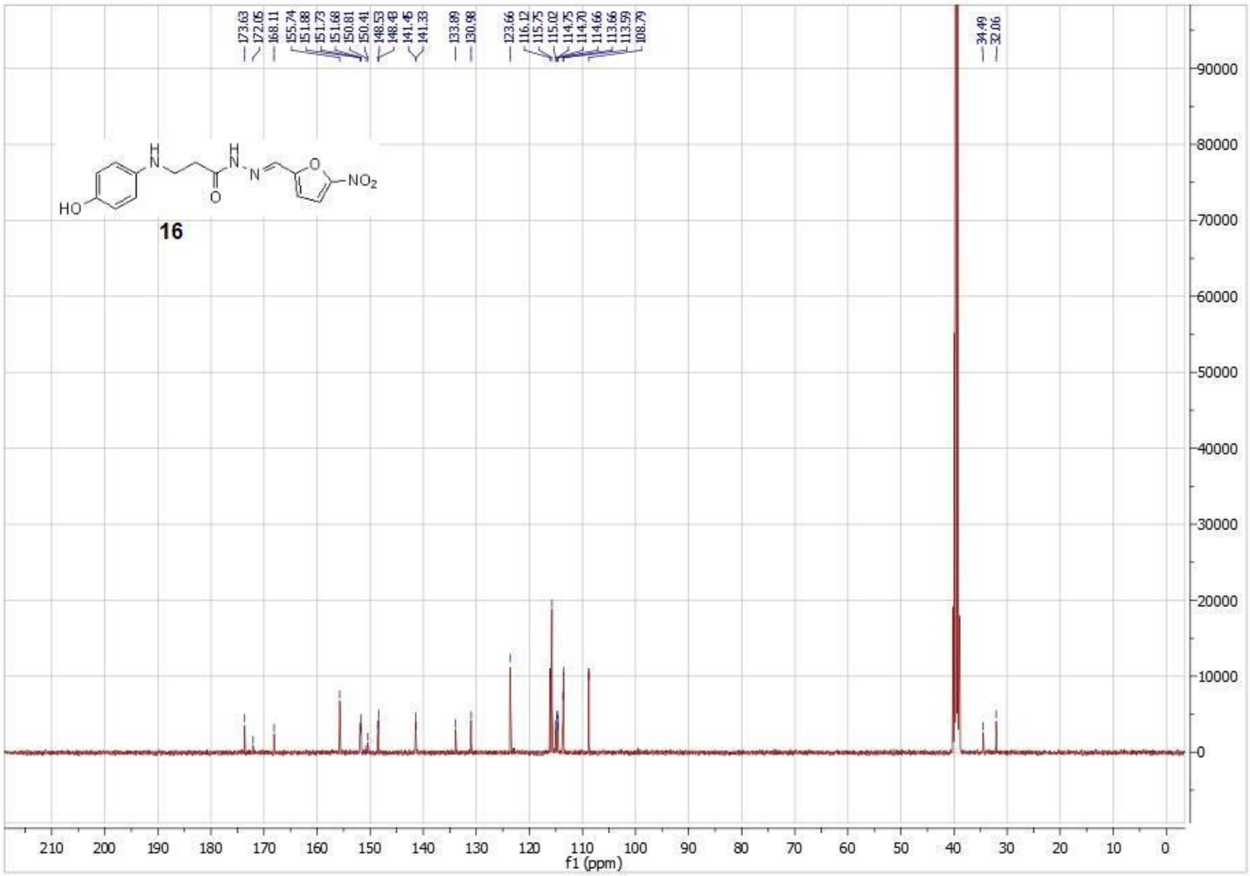

Figure S24. ^{13}C NMR spectrum of compound **16**

N-(2,5-dimethyl-1H-pyrrol-1-yl)-3-((4-hydroxyphenyl)amino)propanamide ***(17)***

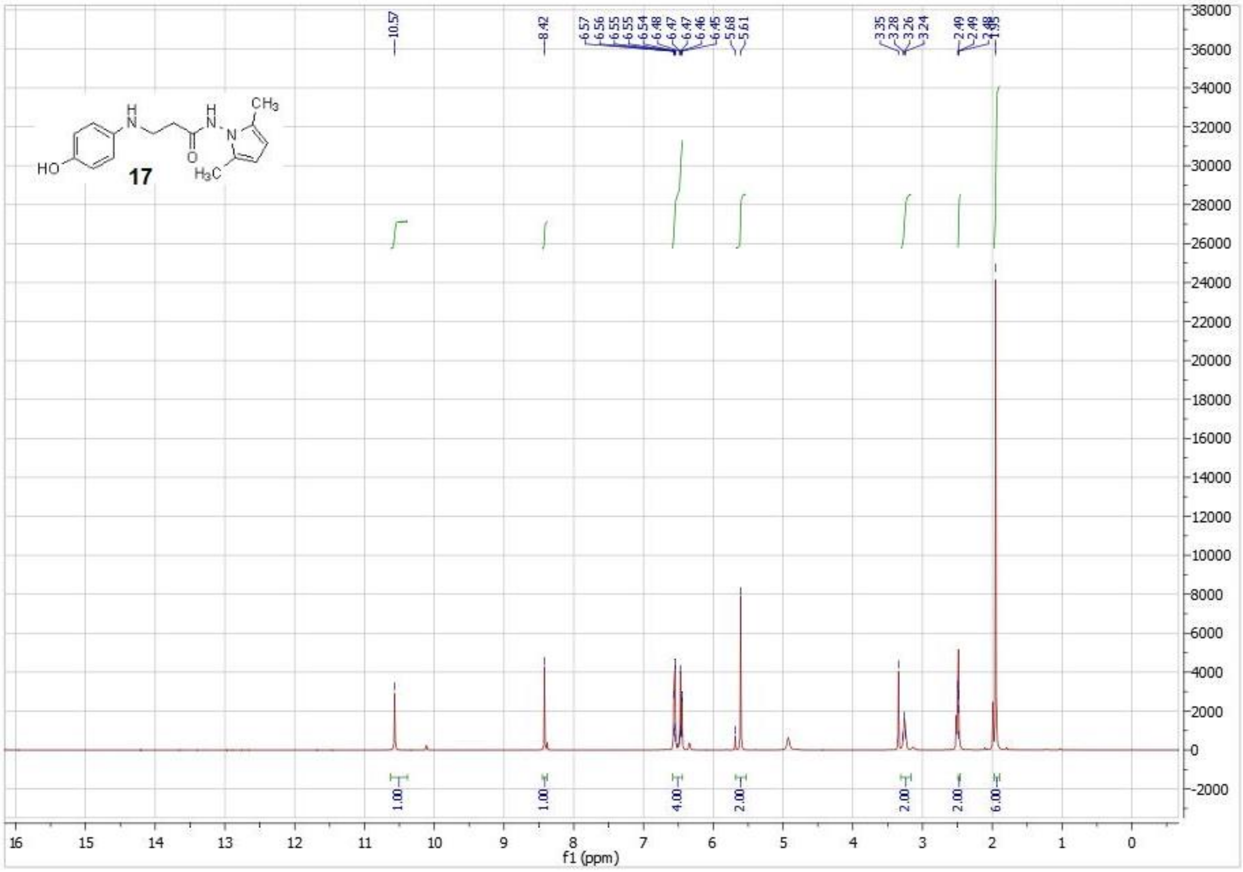

Figure S25. ^{1}H NMR spectrum of compound **17**

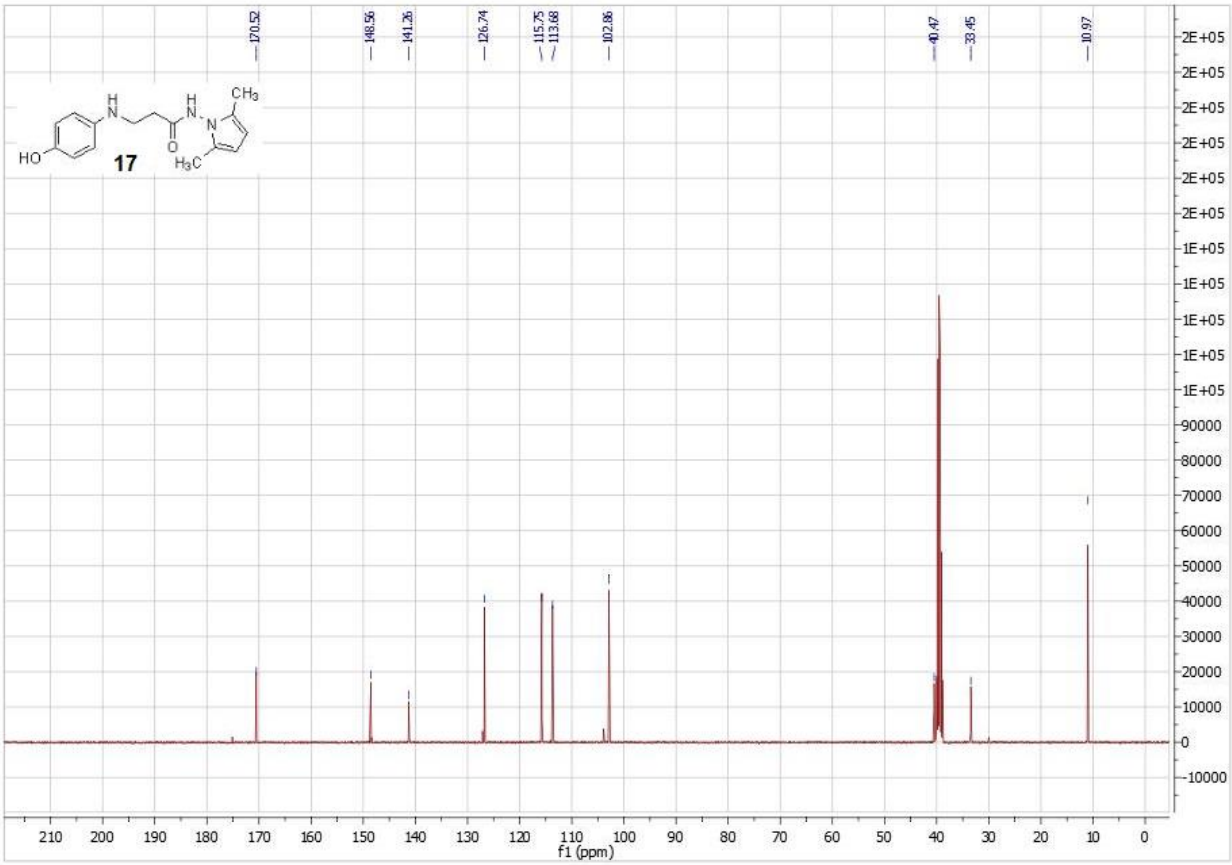

Figure S26. ^{13}C NMR spectrum of compound **17**

3-((4-Hydroxyphenyl)amino)-N'-(2-oxoindolin-3-ylidene)propanehydrazide ***(18)***

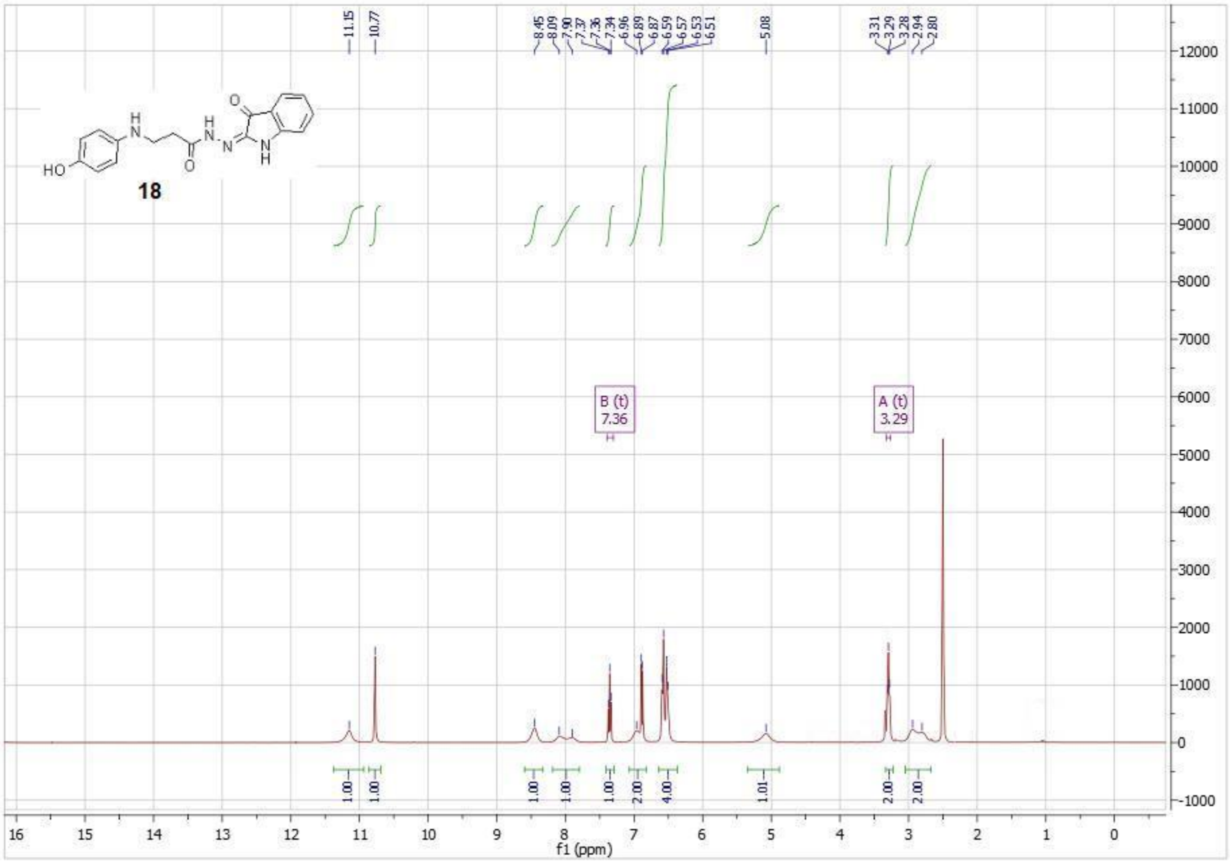

Figure S27. ^{1}H NMR spectrum of compound **18**

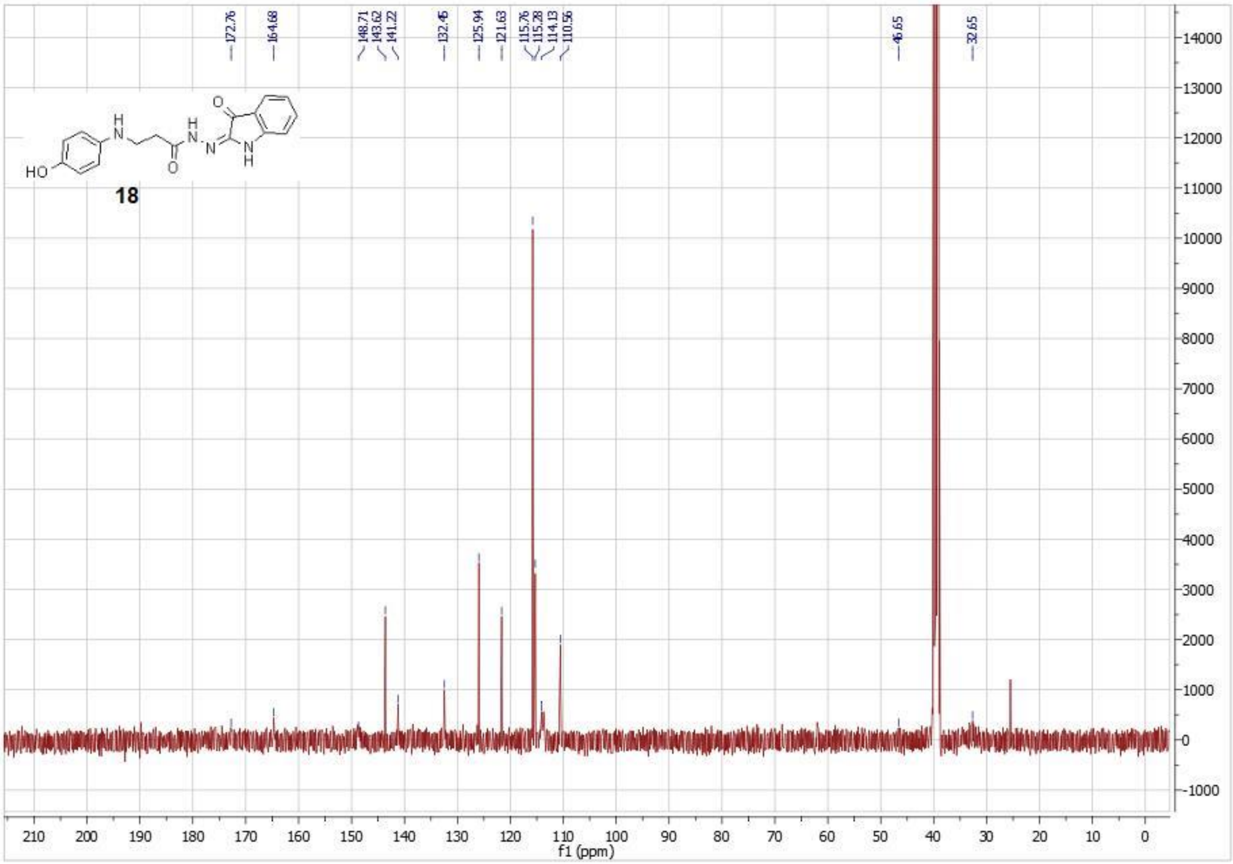

Figure S28. ^{13}C NMR spectrum of compound **18**

3,3'-((4-Hydroxyphenyl)azanediyl)bis(N'-(-(2-oxoindolin-3-yl)methylene)propanehydrazide) ***(19)***

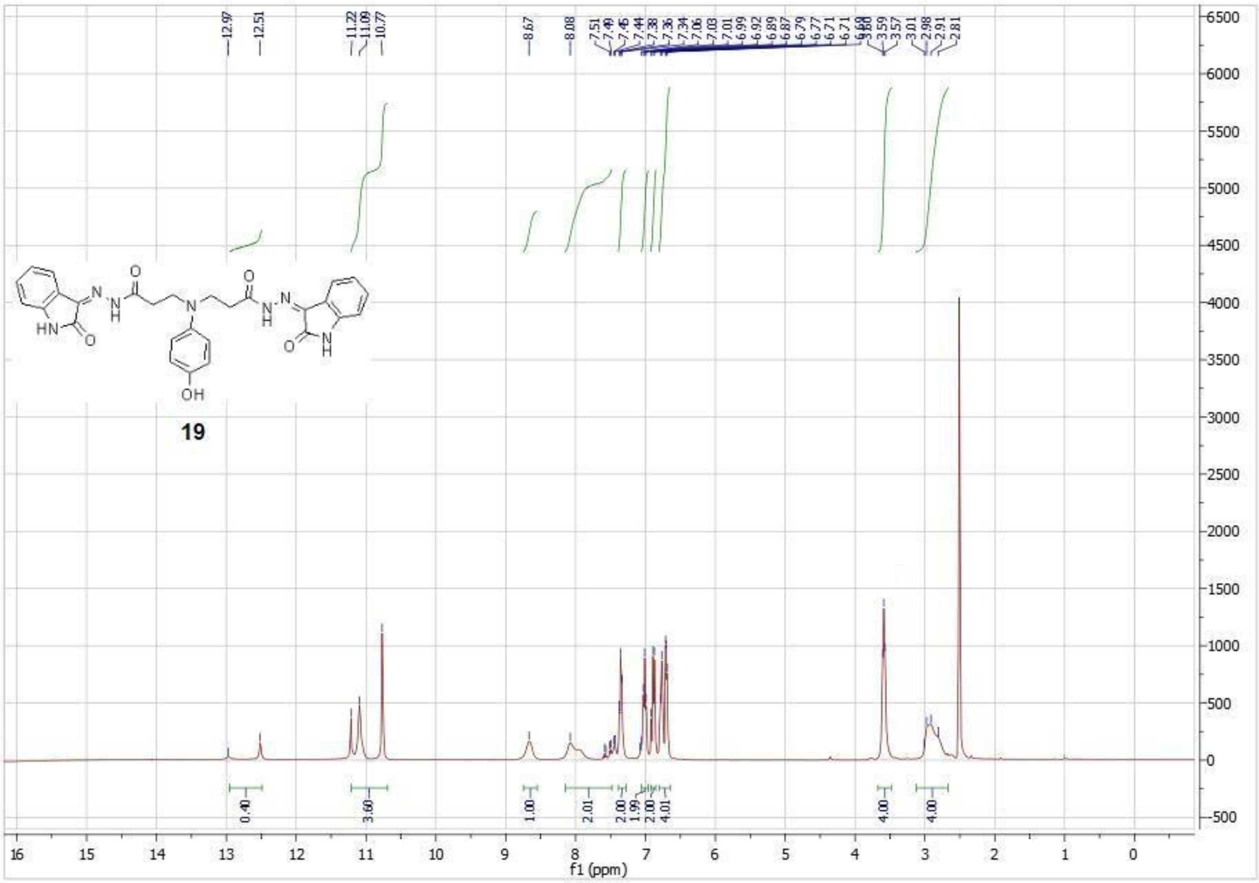

Figure S29. ^{1}H NMR spectrum of compound **19**

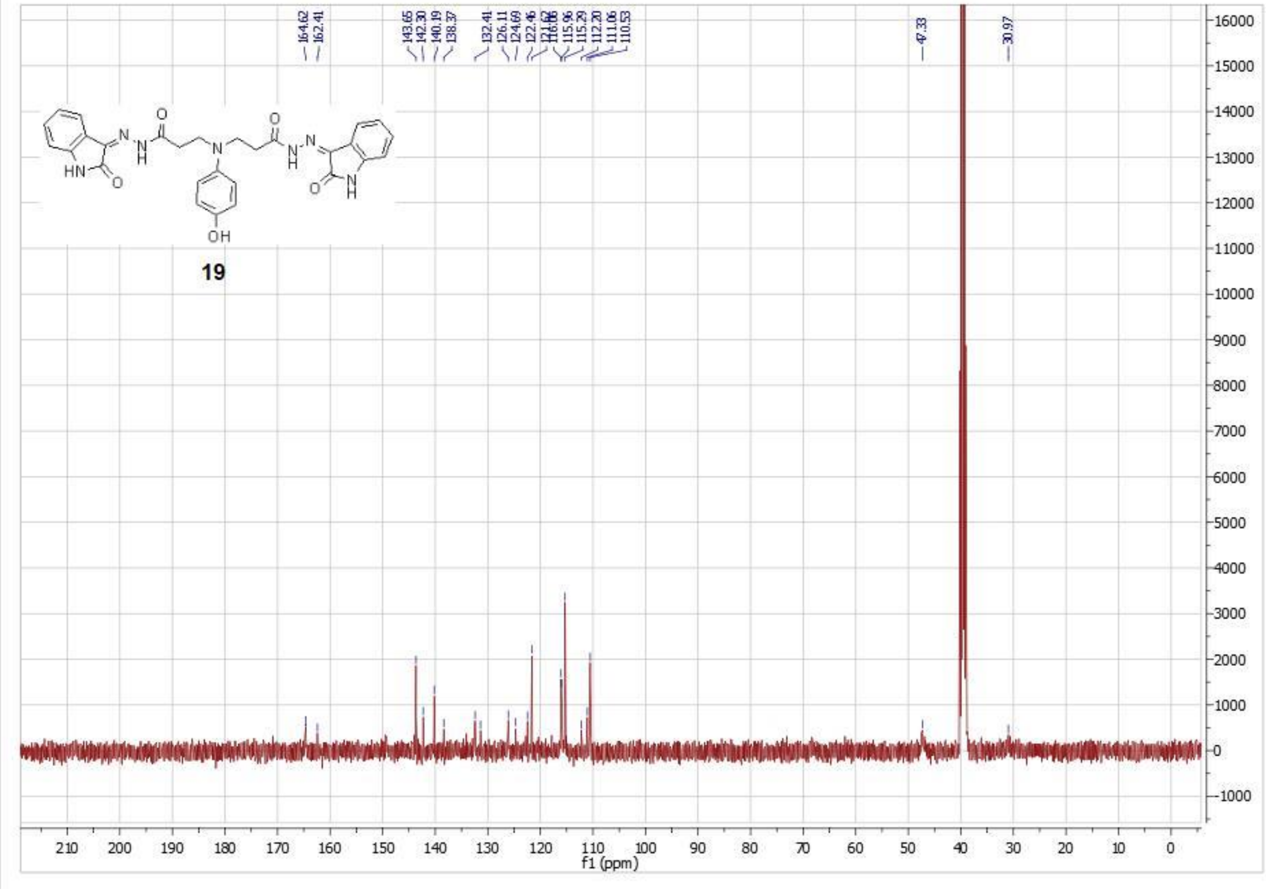

Figure S30. ^{13}C NMR spectrum of compound **19**

5,5'-(((4-Hydroxyphenyl)azanediyl)bis(ethane-2,1-diyl))bis(1,3,4-oxadiazole-2(3H)-thione) ***(20)***

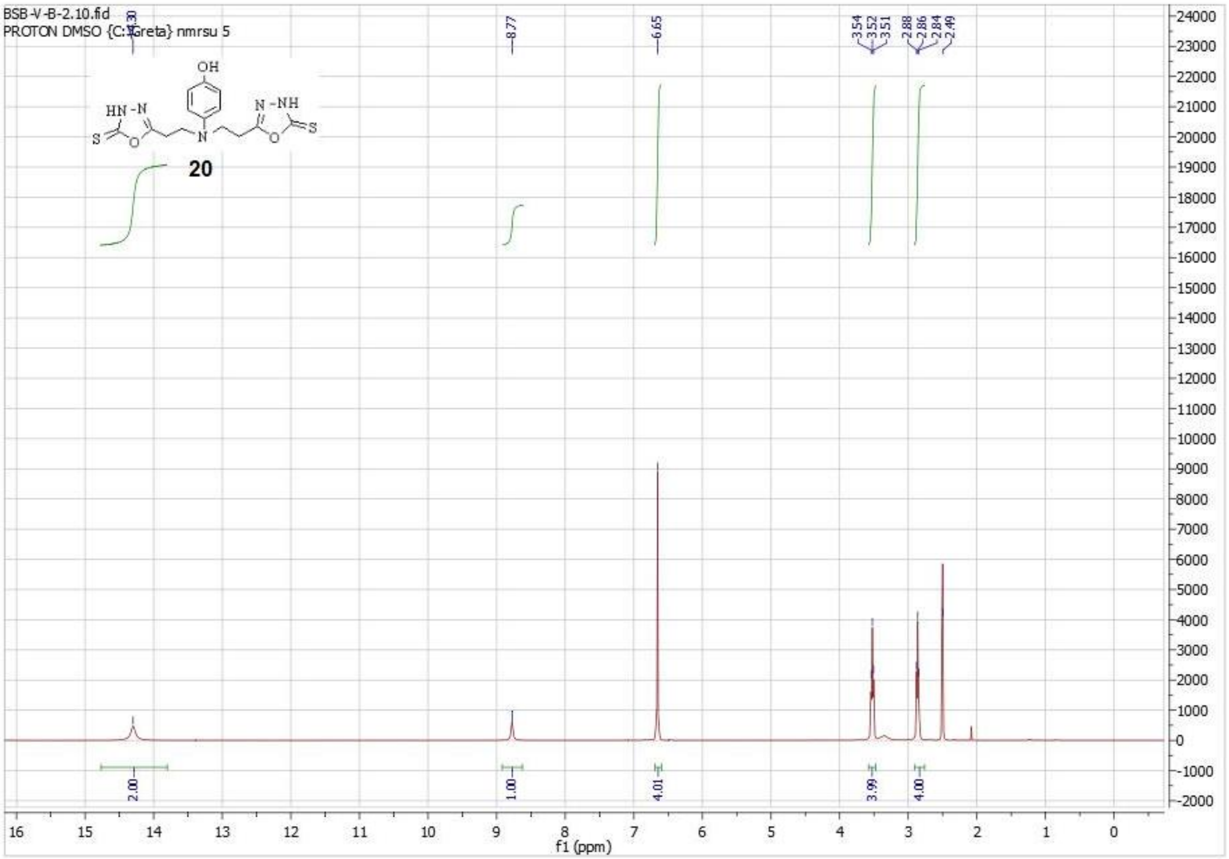

Figure S31. ^{1}H NMR spectrum of compound **20**

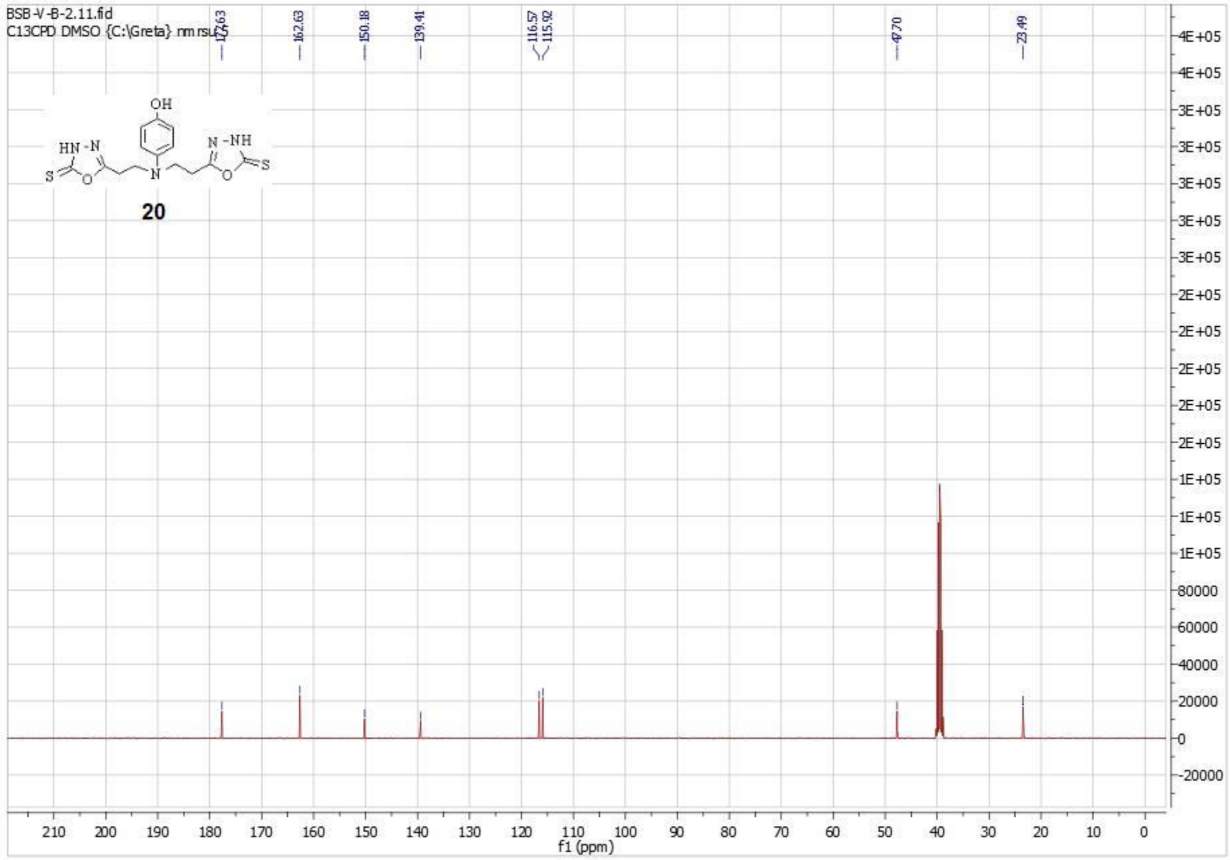

Figure S32. ^{13}C NMR spectrum of compound **20**

3,3'-((4-Hydroxyphenyl)azanediyl)bis(N'-(furan-2-ylmethylene)propanehydrazide) ***(21)***

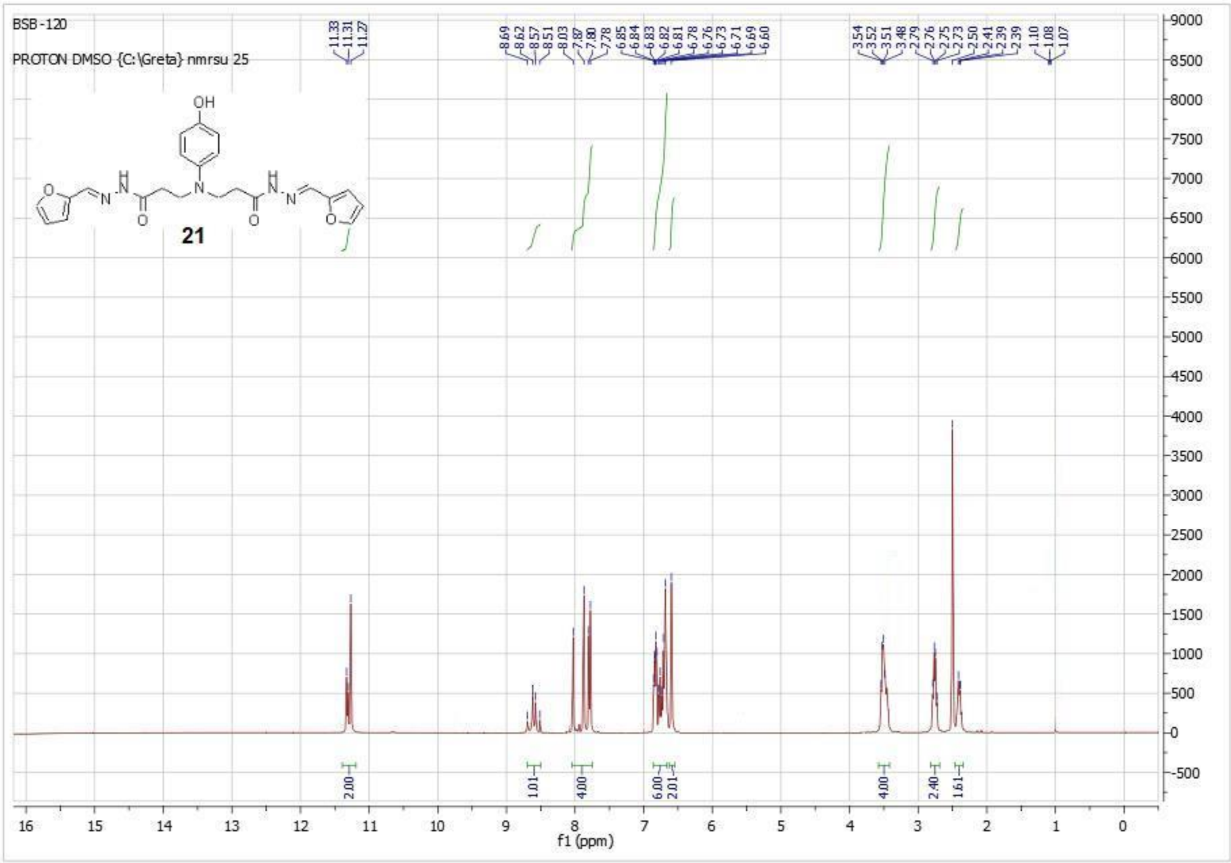

Figure S33. ^{1}H NMR spectrum of compound **21**

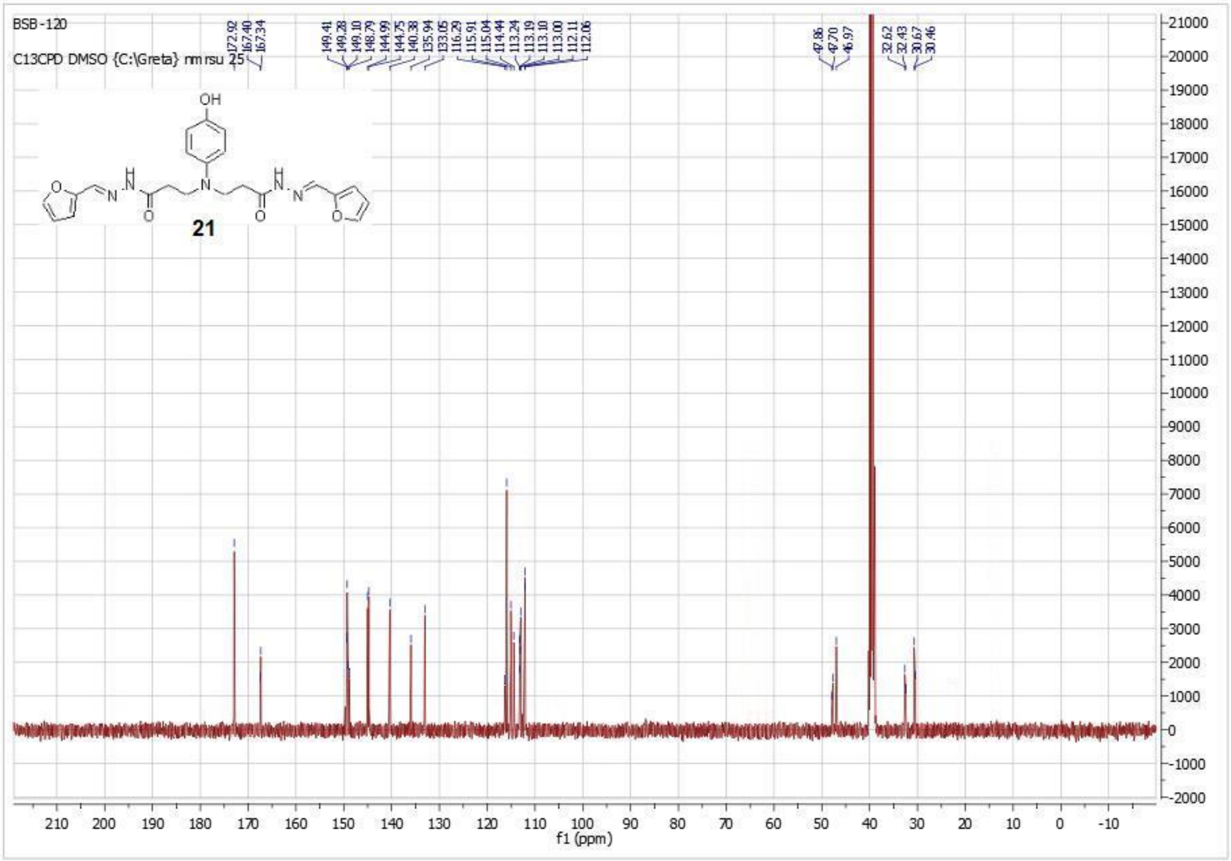

Figure S34. ^{13}C NMR spectrum of compound **21**

3,3'-((4-Hydroxyphenyl)azanediyl)bis(N'-(thien-2-ylmethylene)propanehydrazide) ***(22)***

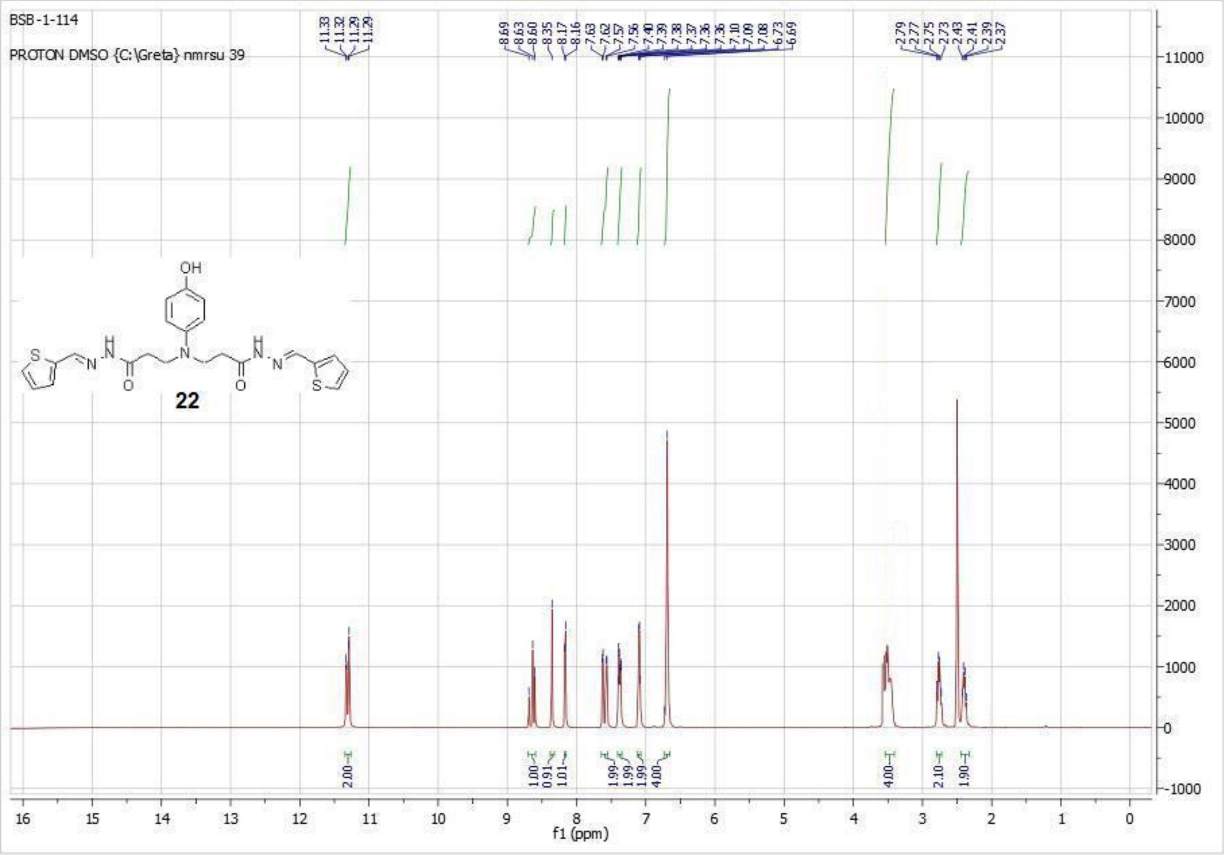

Figure S35. ^{1}H NMR spectrum of compound **22**

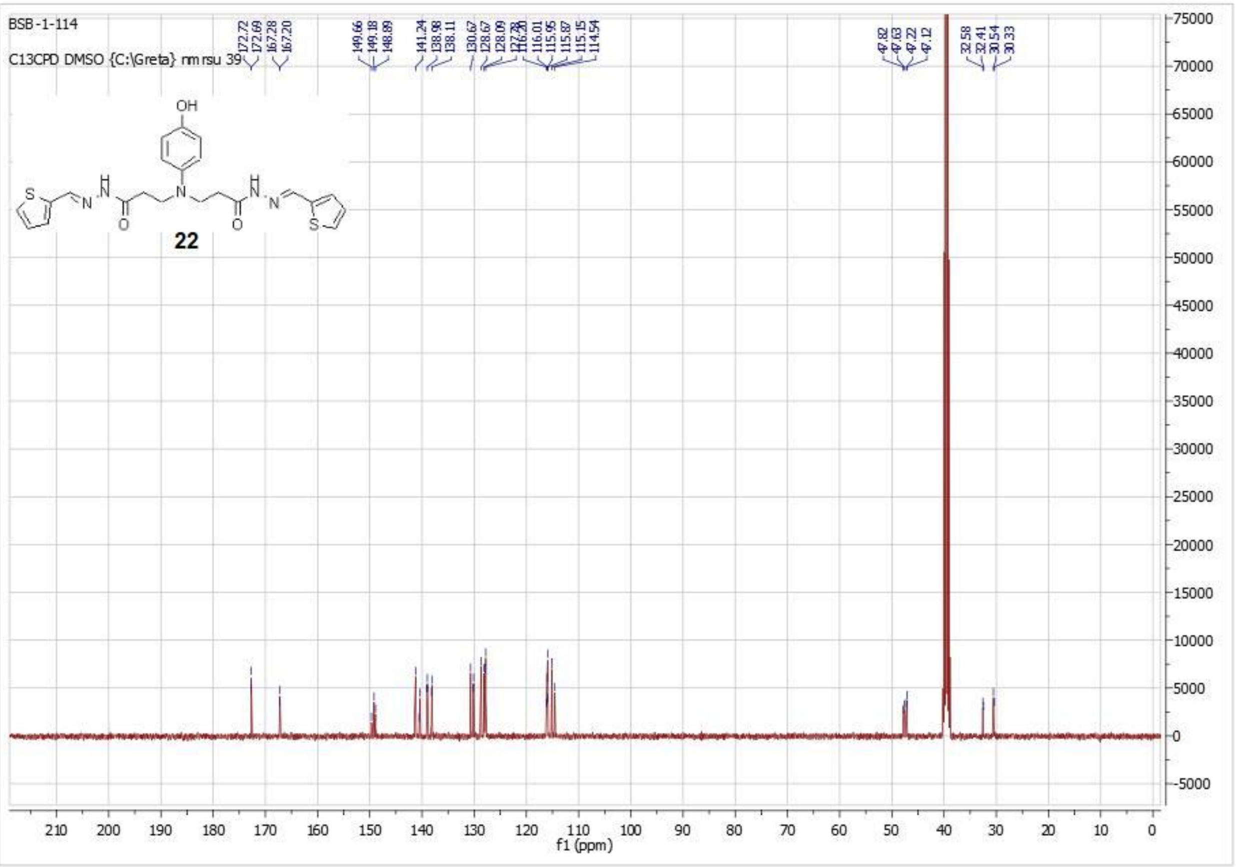

Figure S36. ^{13}C NMR spectrum of compound **22**

3,3'-((4-Hydroxyphenyl)azanediyl)bis(N'-(-(5-nitrothien-2yl)methylene)propanehydrazide) ***(23)***

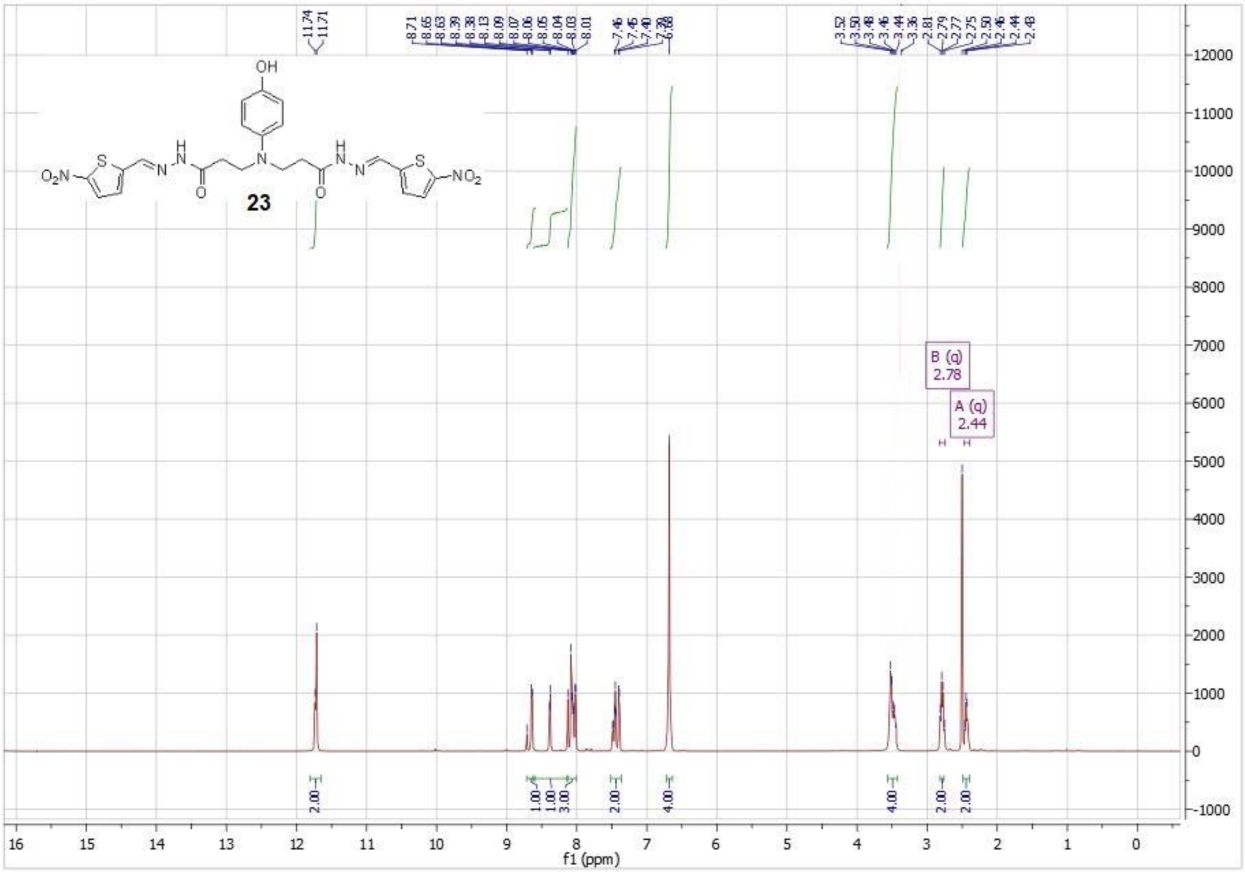

Figure S37. ^{1}H NMR spectrum of compound **23**

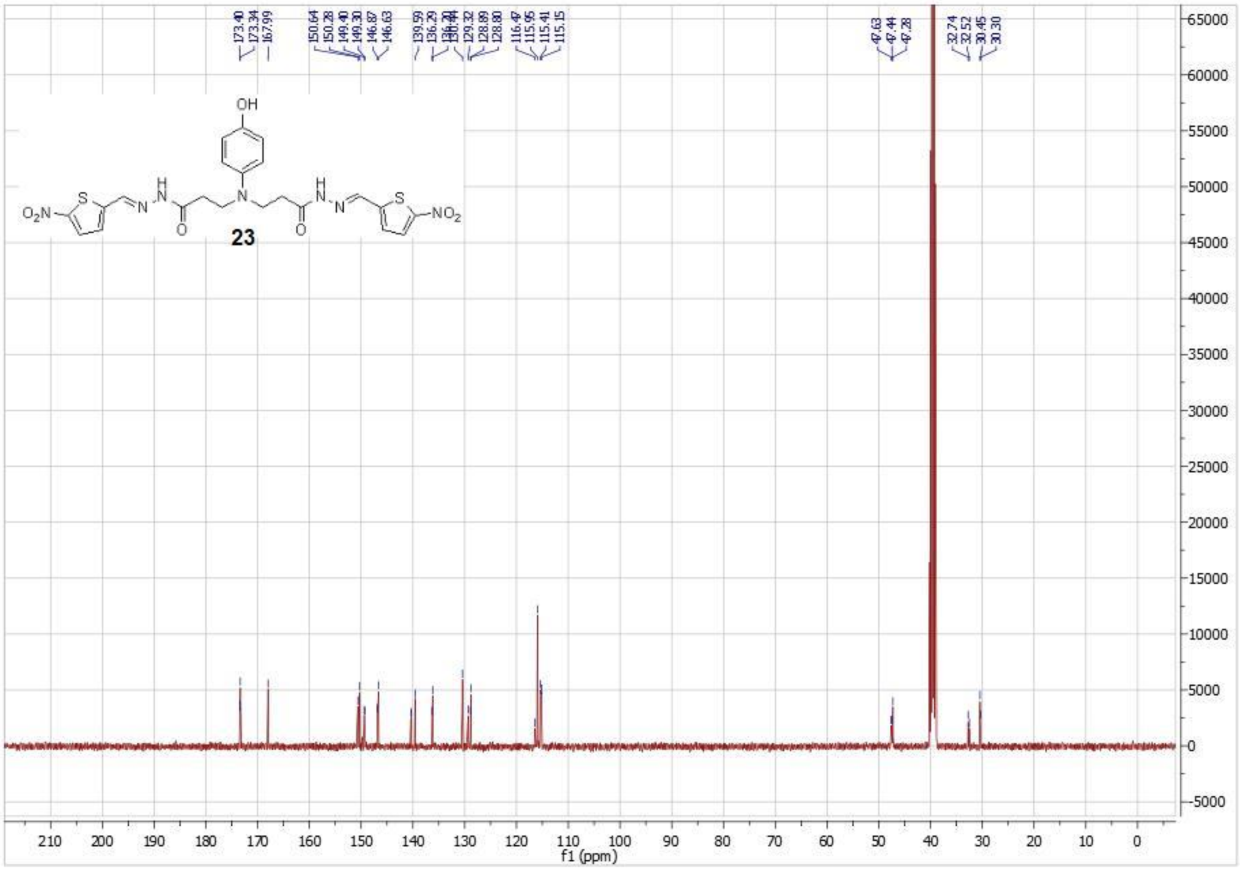

Figure S38. ^{13}C NMR spectrum of compound **23**

3,3'-((4-Hydroxyphenyl)azanediyl)bis(N'-((5-nitrofuran-2-yl)methylene)propanehydrazide) ***(24)***

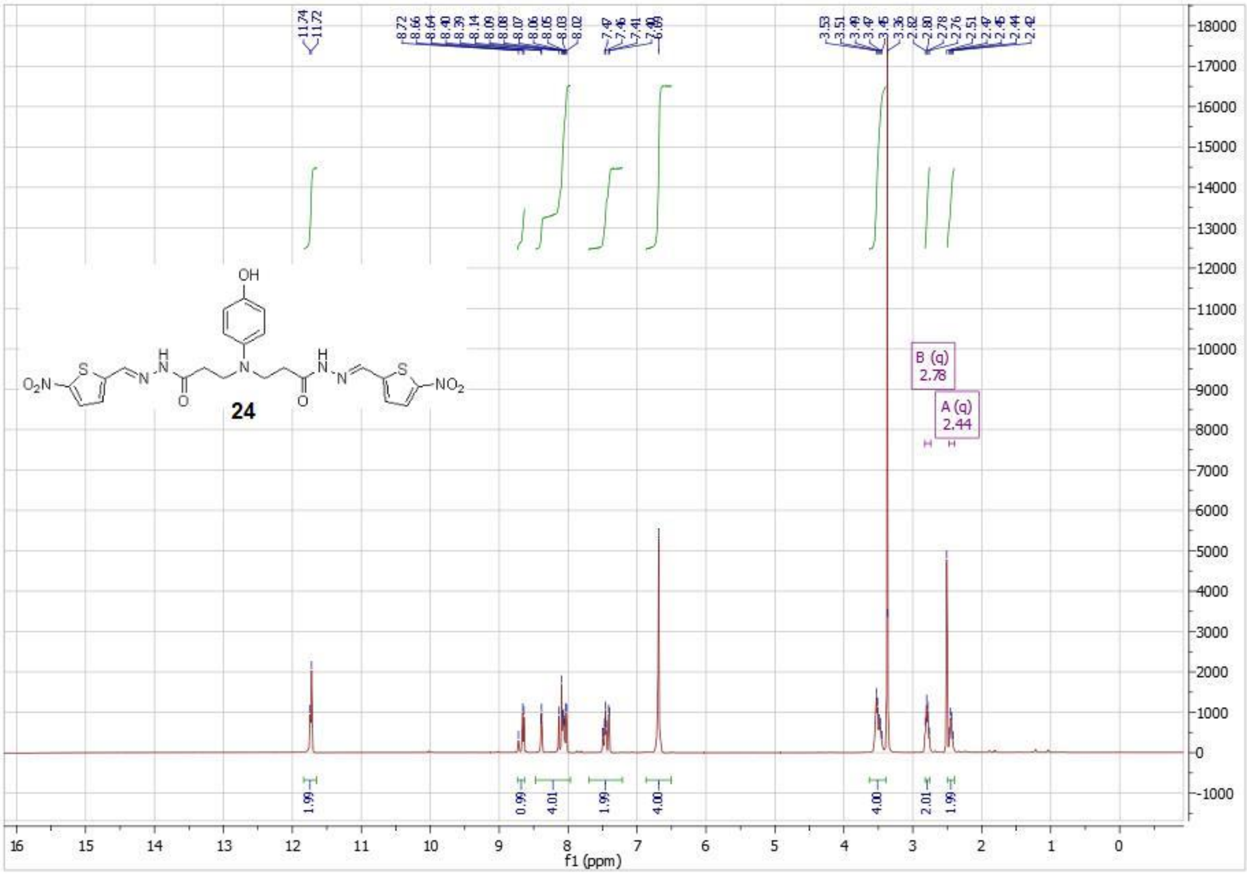

Figure S39. ^{1}H NMR spectrum of compound **24**

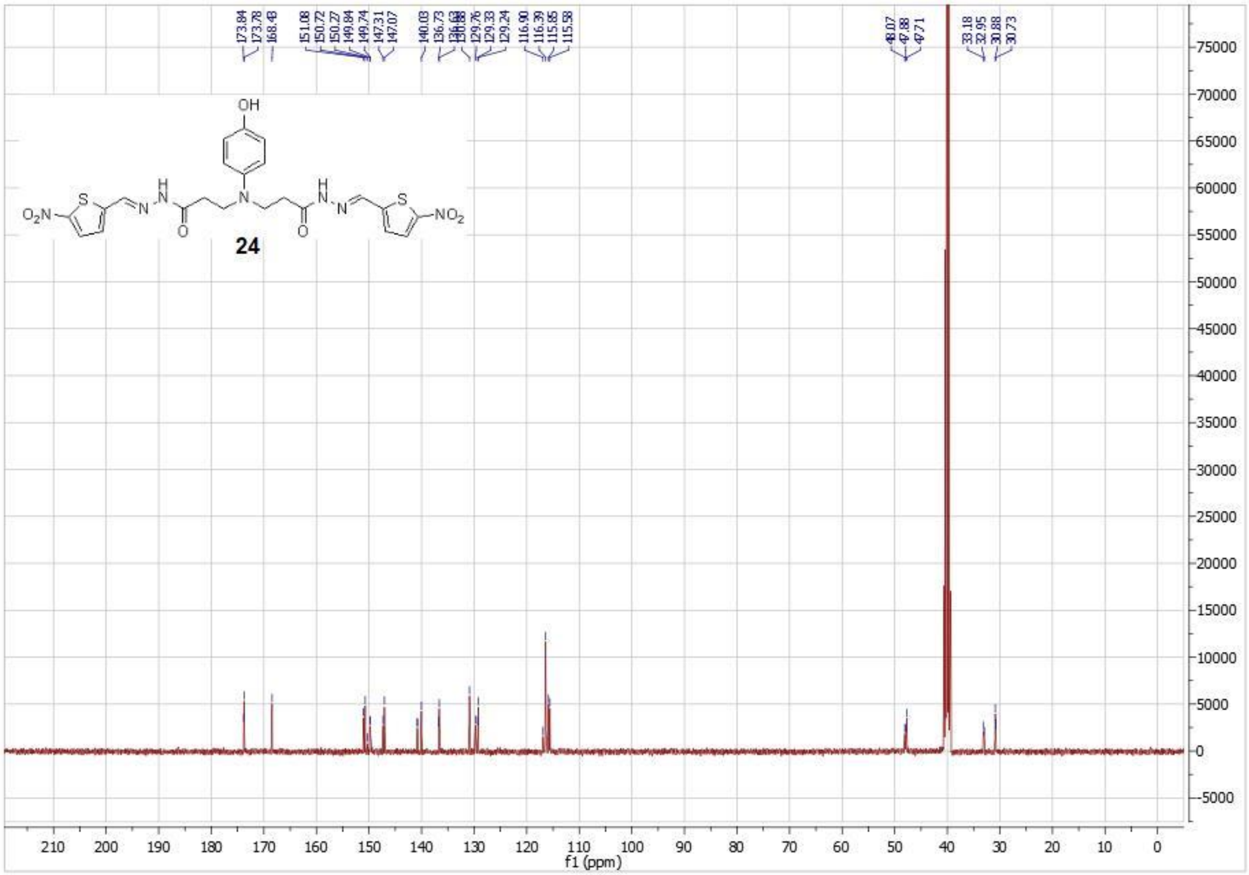

Figure S40. ^{13}C NMR spectrum of compound **24**

3,3'-((4-Hydroxyphenyl)azanediyl)bis(N'-(thien-3-ylmethylene)propanehydrazide) ***(25)***

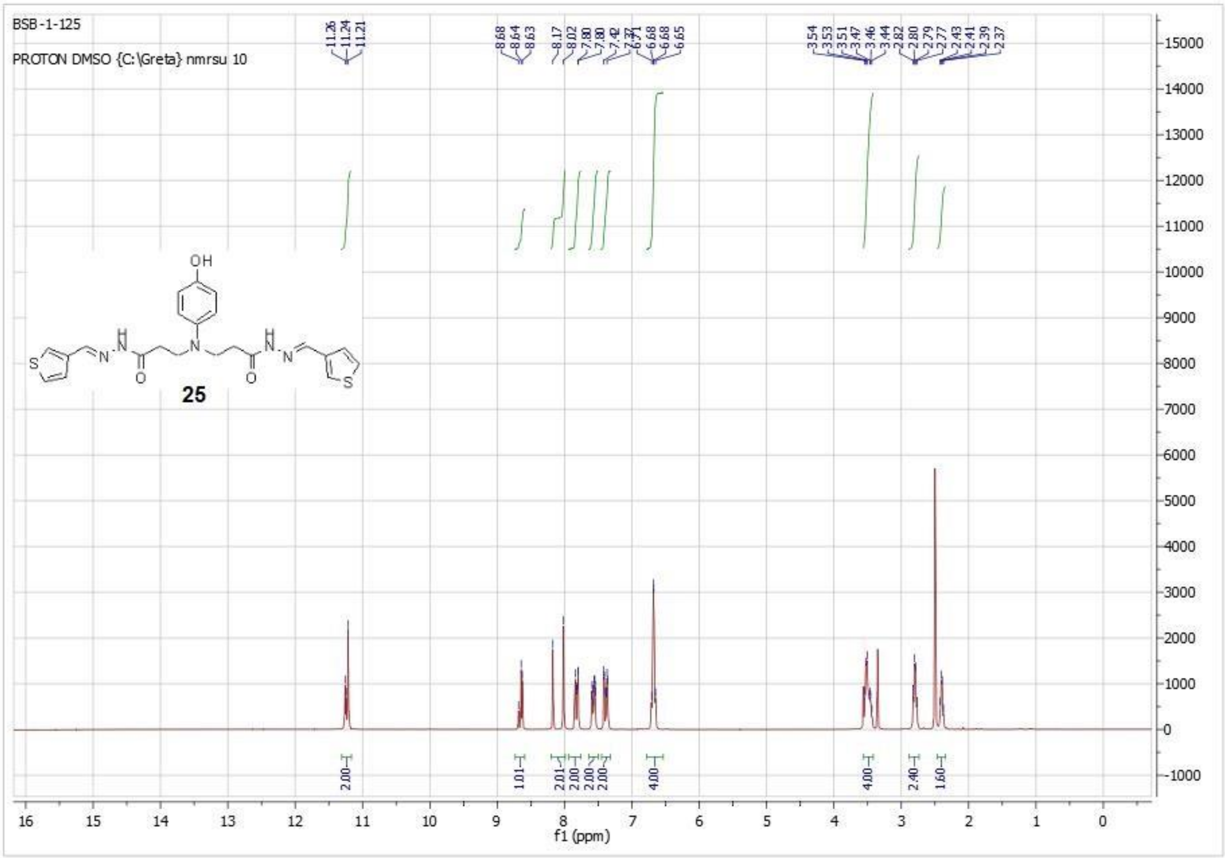

Figure S41. ^{1}H NMR spectrum of compound **25**

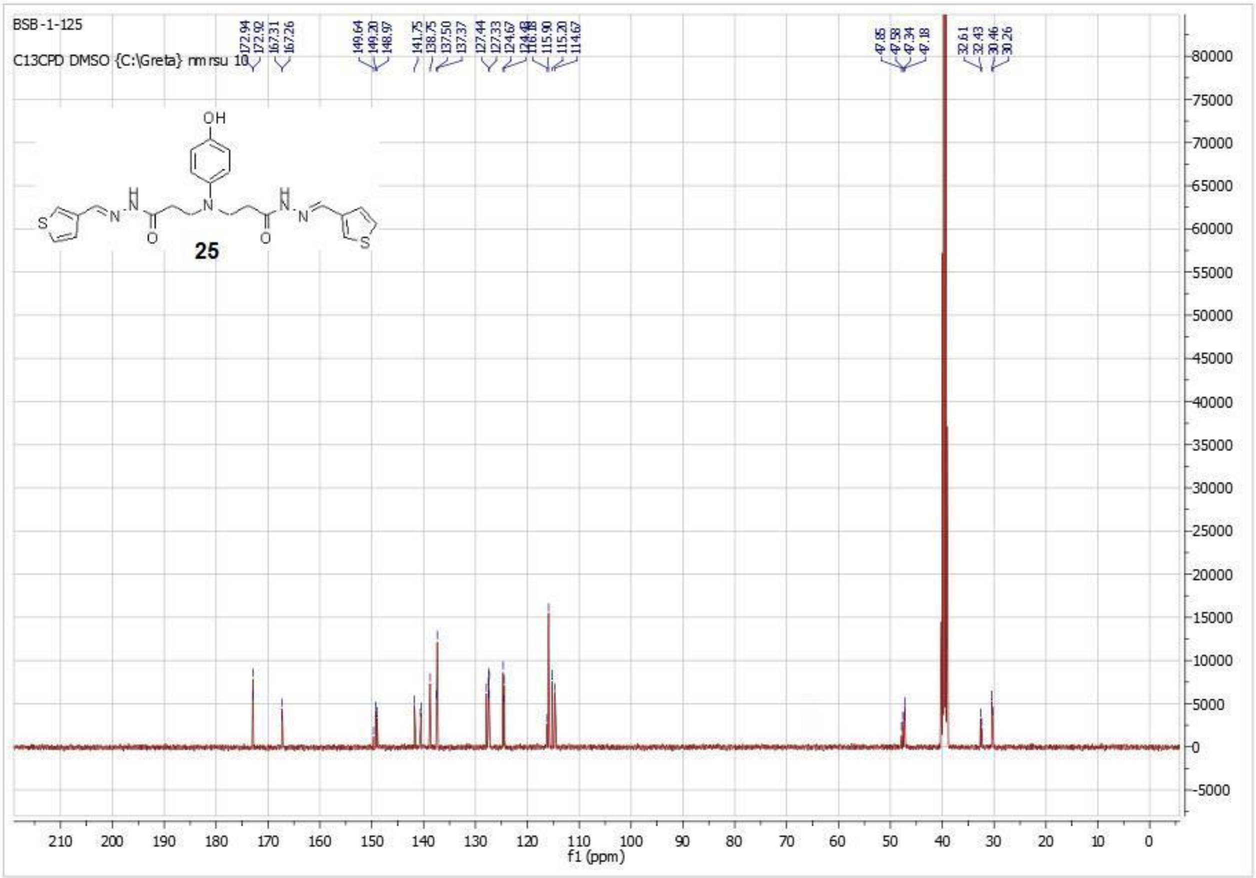

Figure S42. ^{13}C NMR spectrum of compound **25**

3,3'-((4-Hydroxyphenyl)azanediyl)bis(N'-(propan-2-ylidene)propanehydrazide) ***(26)***

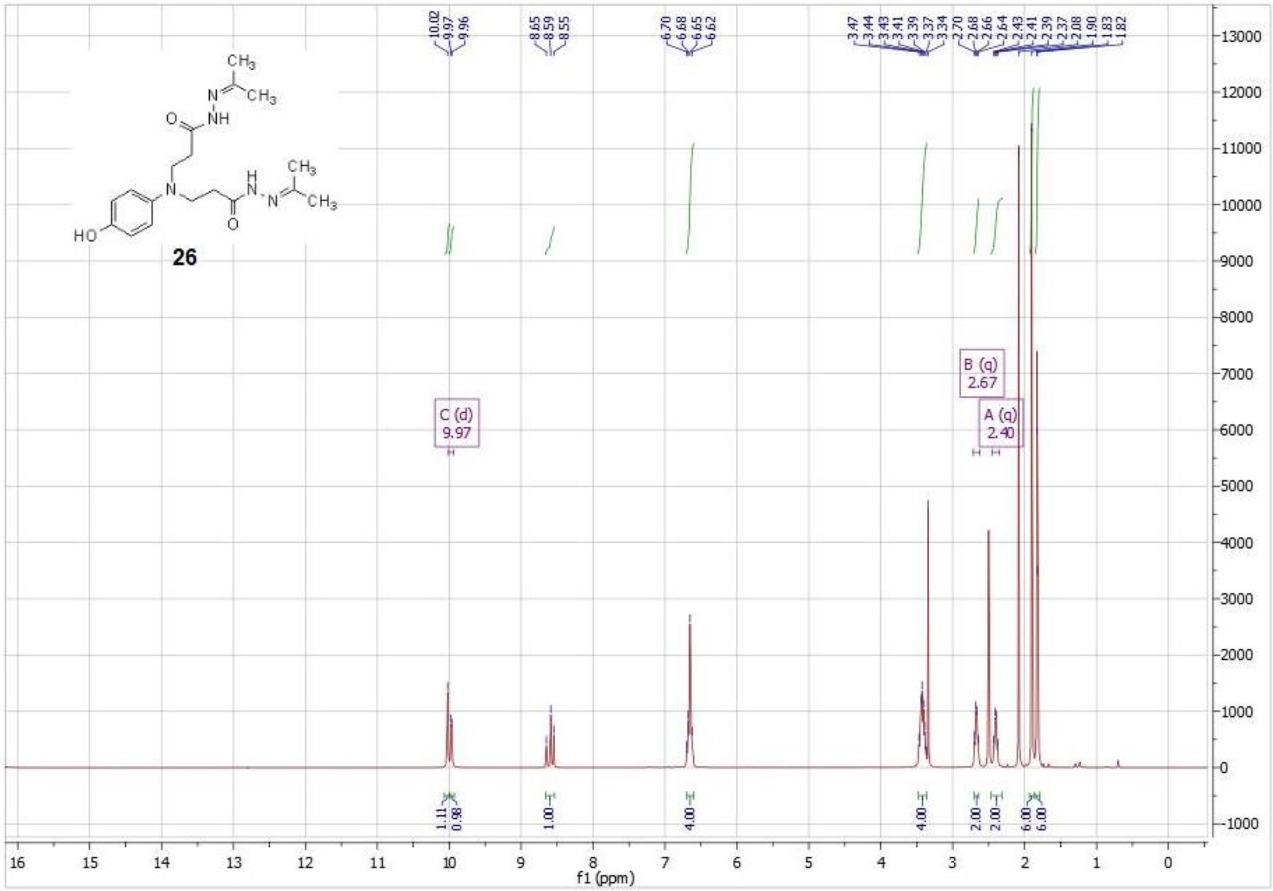

Figure S43. ^{1}H NMR spectrum of compound **26**

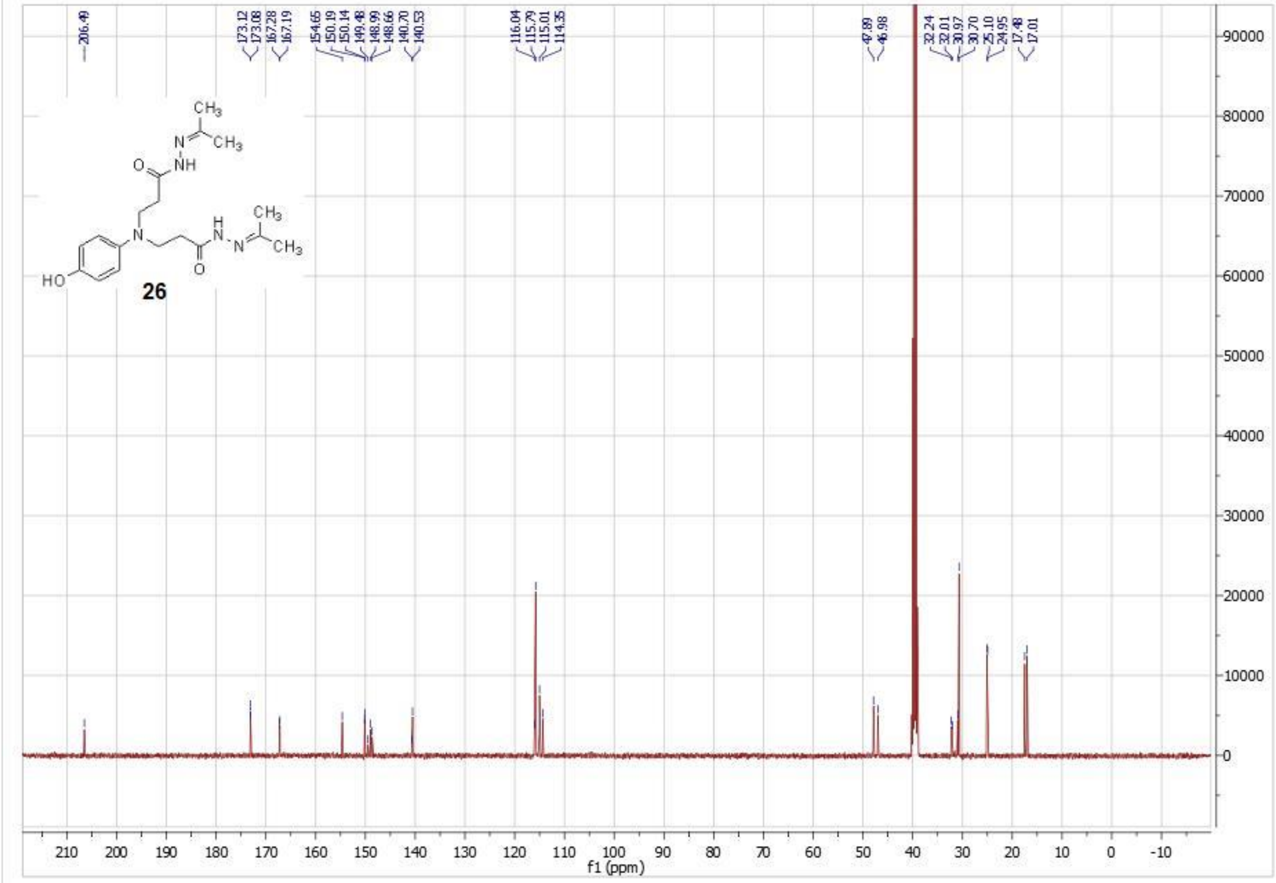

Figure S44. ^{13}C NMR spectrum of compound **26**

3,3'-((4-Hydroxyphenyl)azanediyl)bis(N'-(butan-2-ylidene)propanehydrazide) ***(27)***

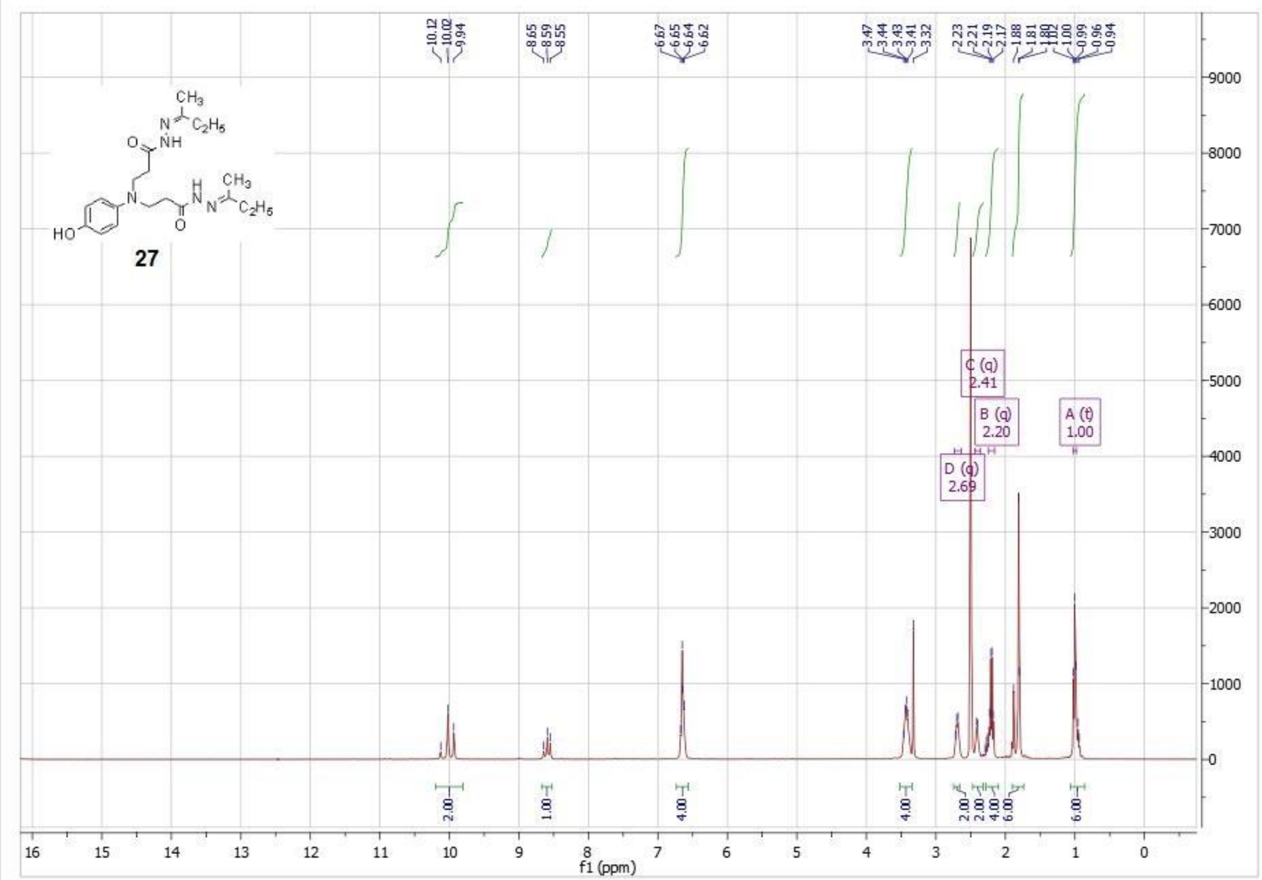

Figure S45. ^{1}H NMR spectrum of compound **27**

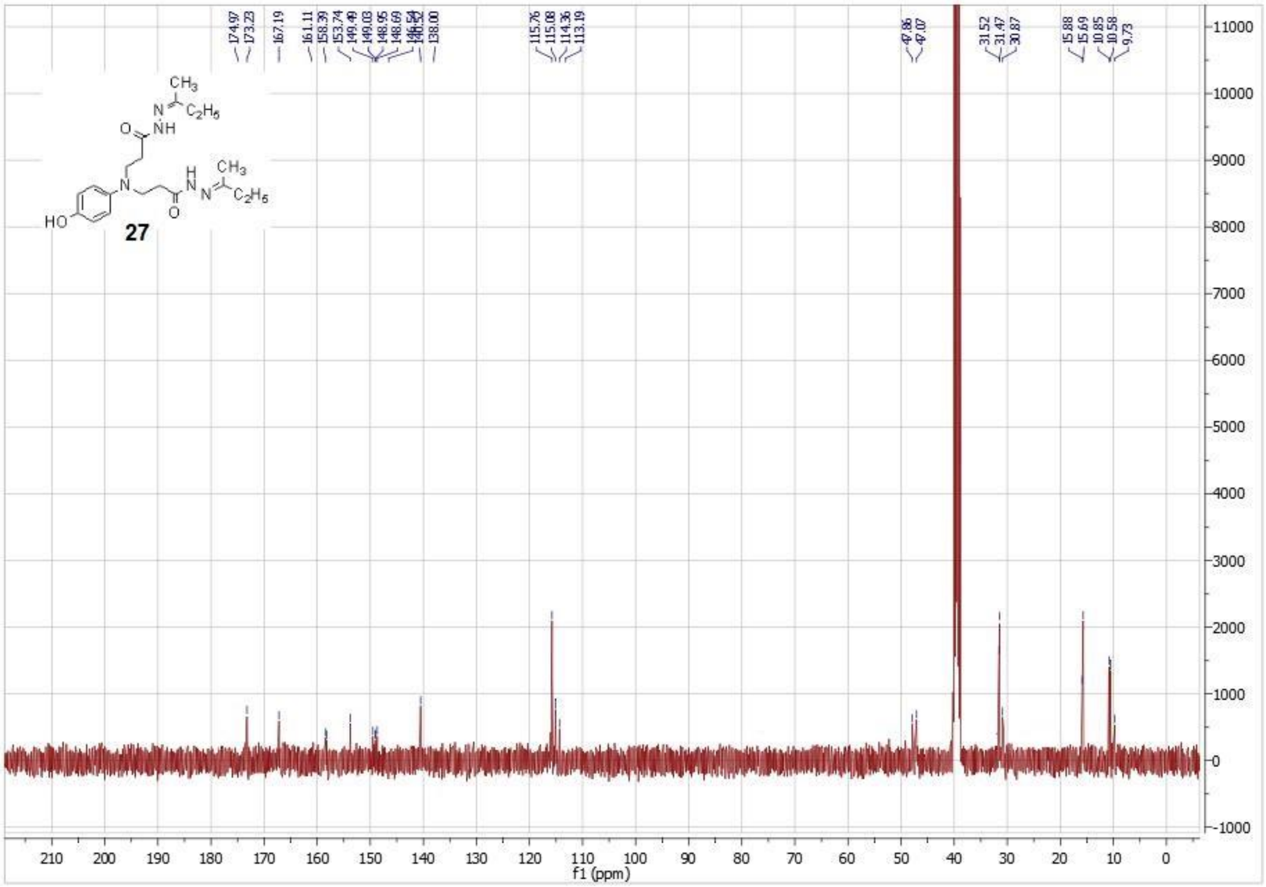

Figure S46. ^{13}C NMR spectrum of compound **27**

3,3'-((4-Hydroxyphenyl)azanediyl)bis(N'-(1-phenylethylidene)propanehydrazide) ***(28)***

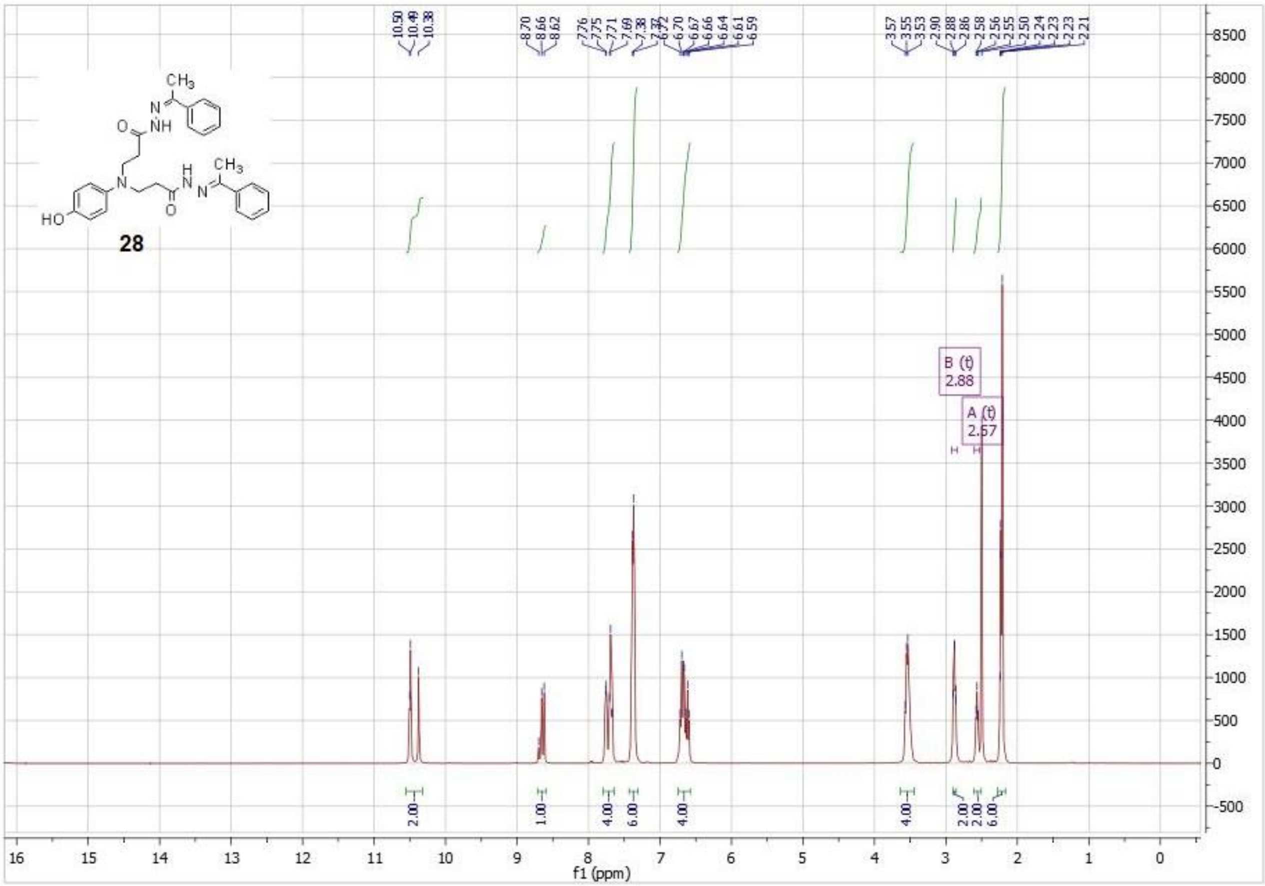

Figure S47. ^{1}H NMR spectrum of compound **28**

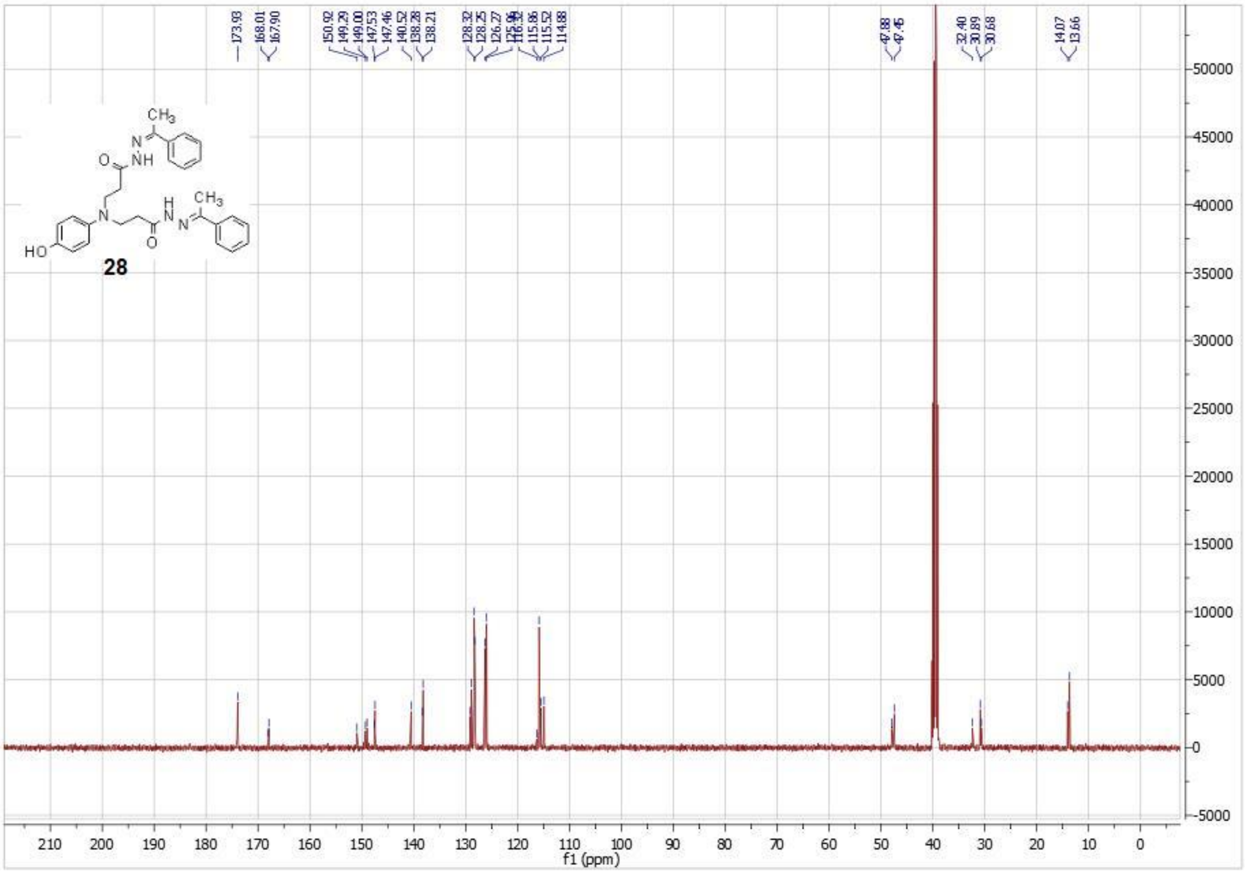

Figure S48. ^{13}C NMR spectrum of compound **28**

3,3'-((4-Hydroxyphenyl)azanediyl)bis(N'-(benzylidene)propanehydrazide) ***(29)***

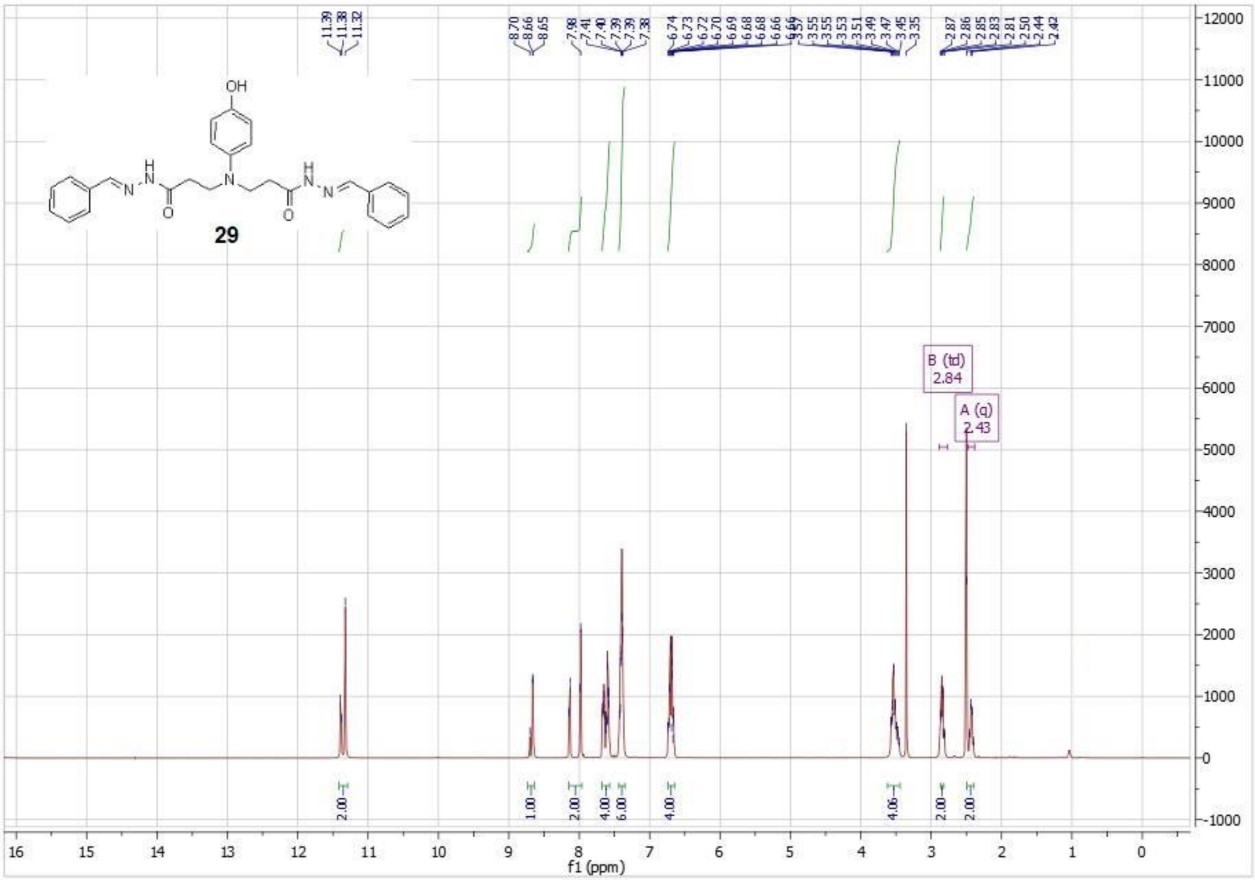

Figure S49. ¹H NMR spectrum of compound **29**

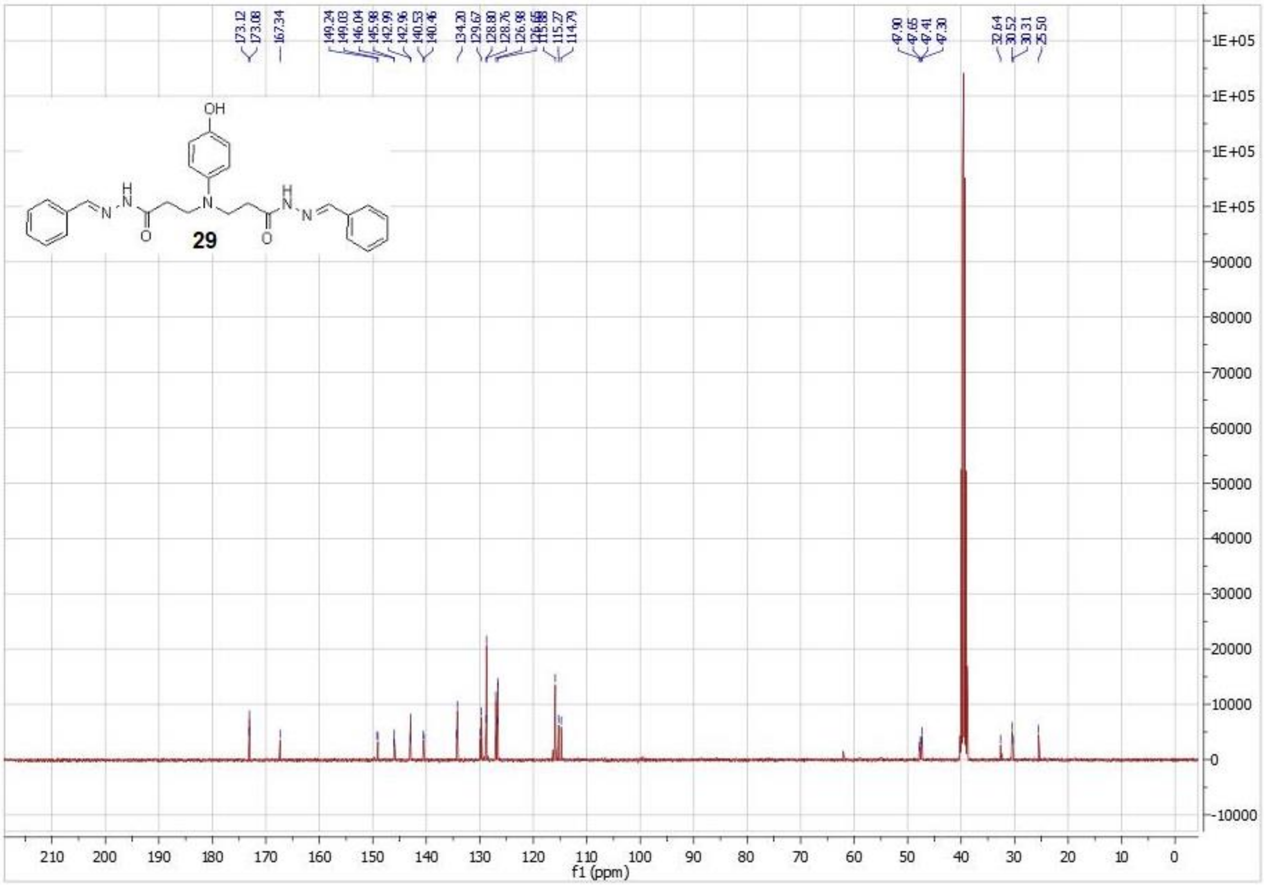

Figure S50. ¹³C NMR spectrum of compound **29**

3,3'-((4-Hydroxyphenyl)azanediyl)bis(N'-(4-nitrobenzylidene)propanehydrazide) ***(30)***

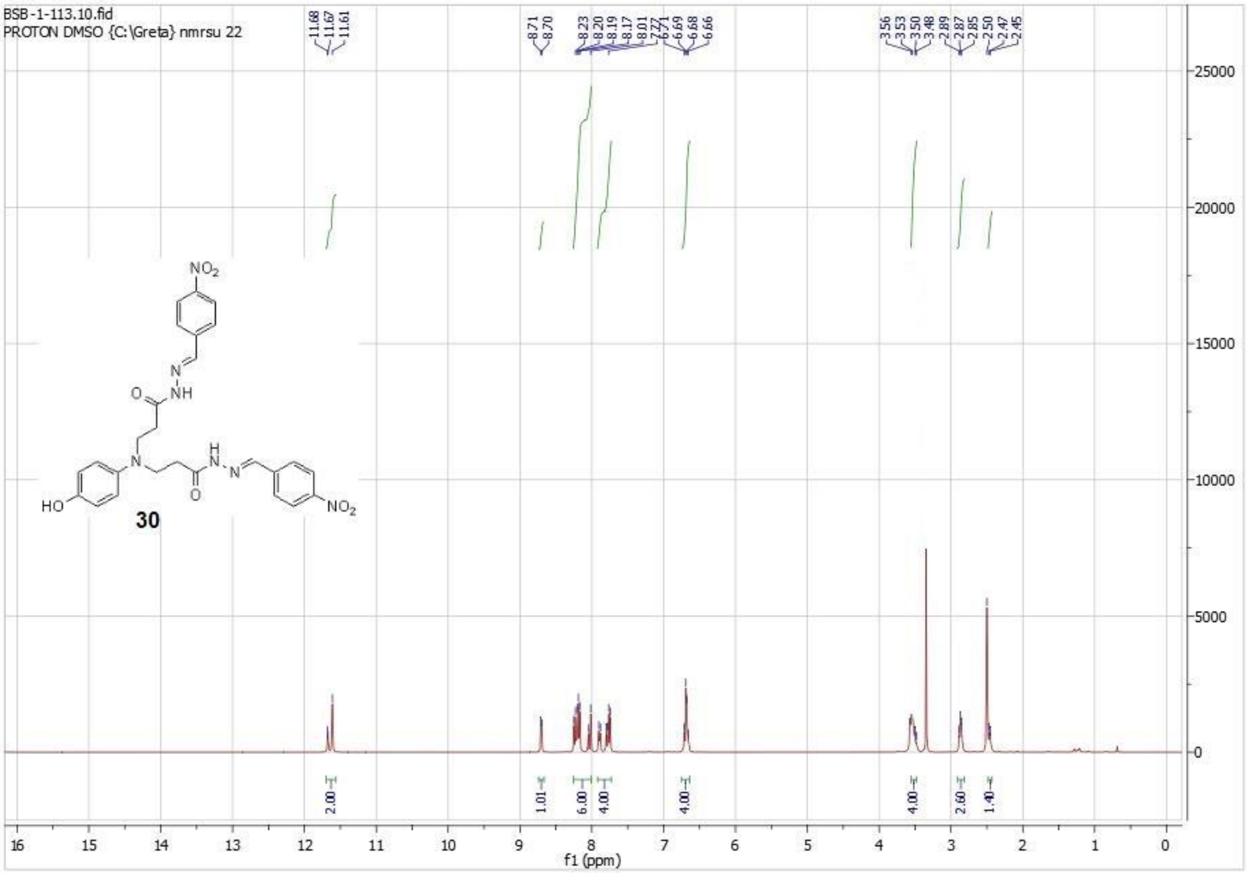

Figure S51. ^{1}H NMR spectrum of compound **30**

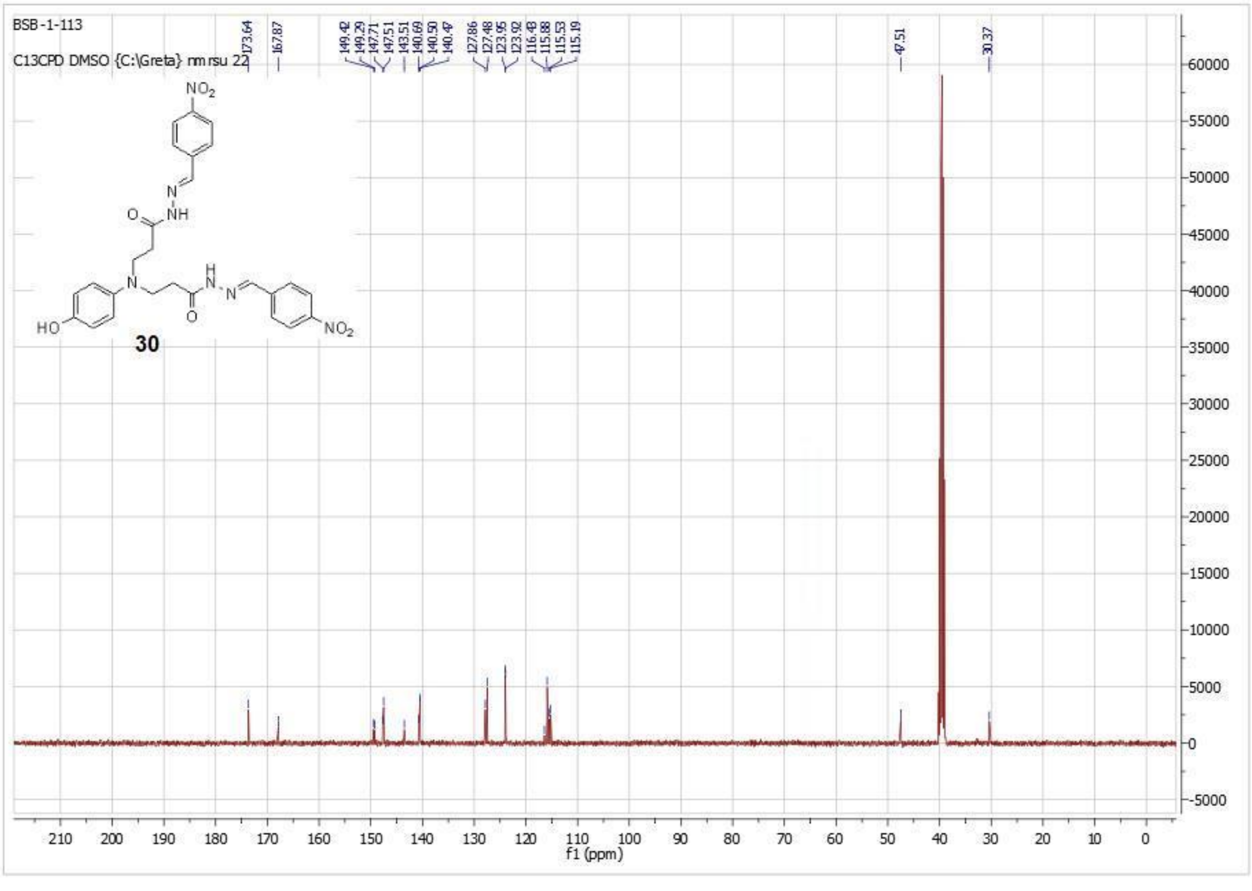

Figure S52. ^{13}C NMR spectrum of compound **30**

3,3'-((4-Hydroxyphenyl)azanediyl)bis(N'-(benzylidene)propanehydrazide) ***(31)***

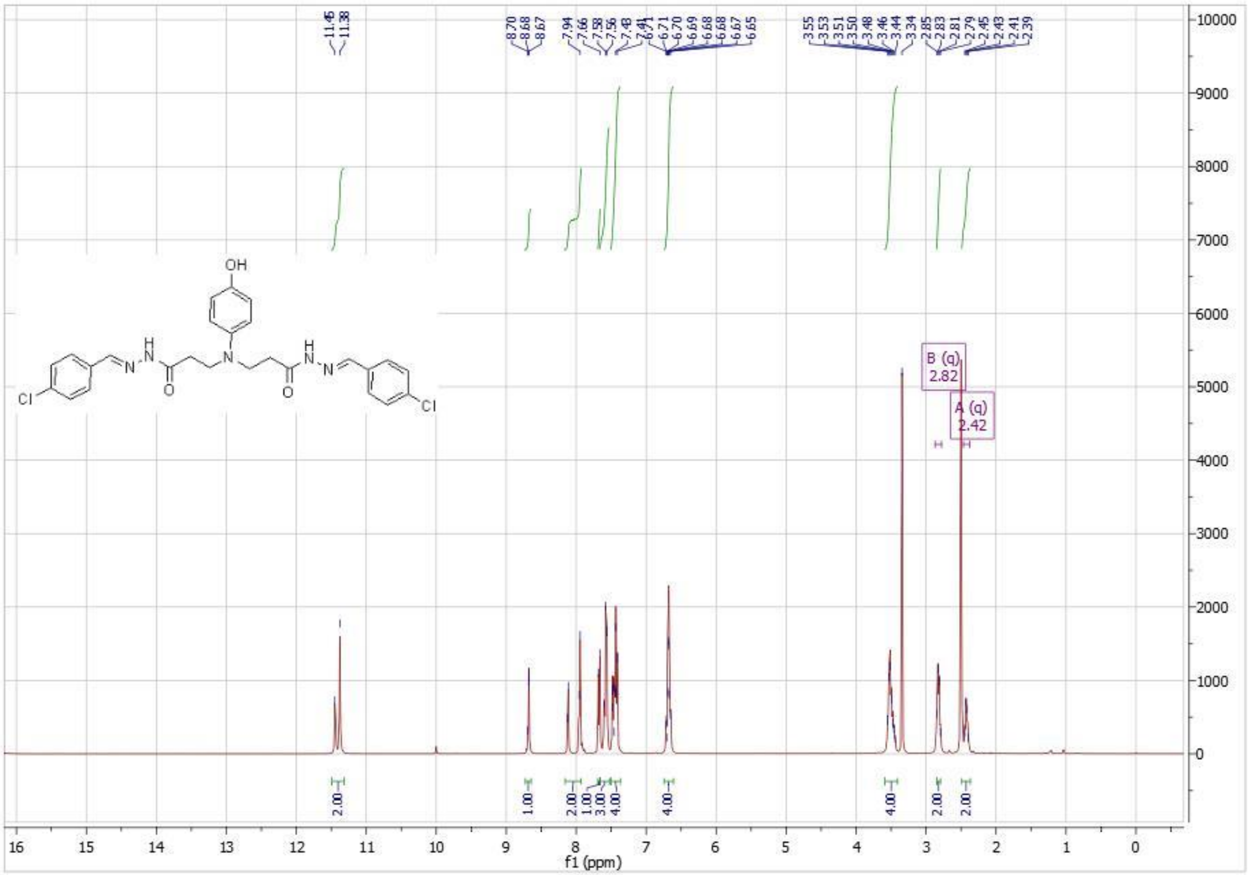

Figure S53. ^{1}H NMR spectrum of compound **31**

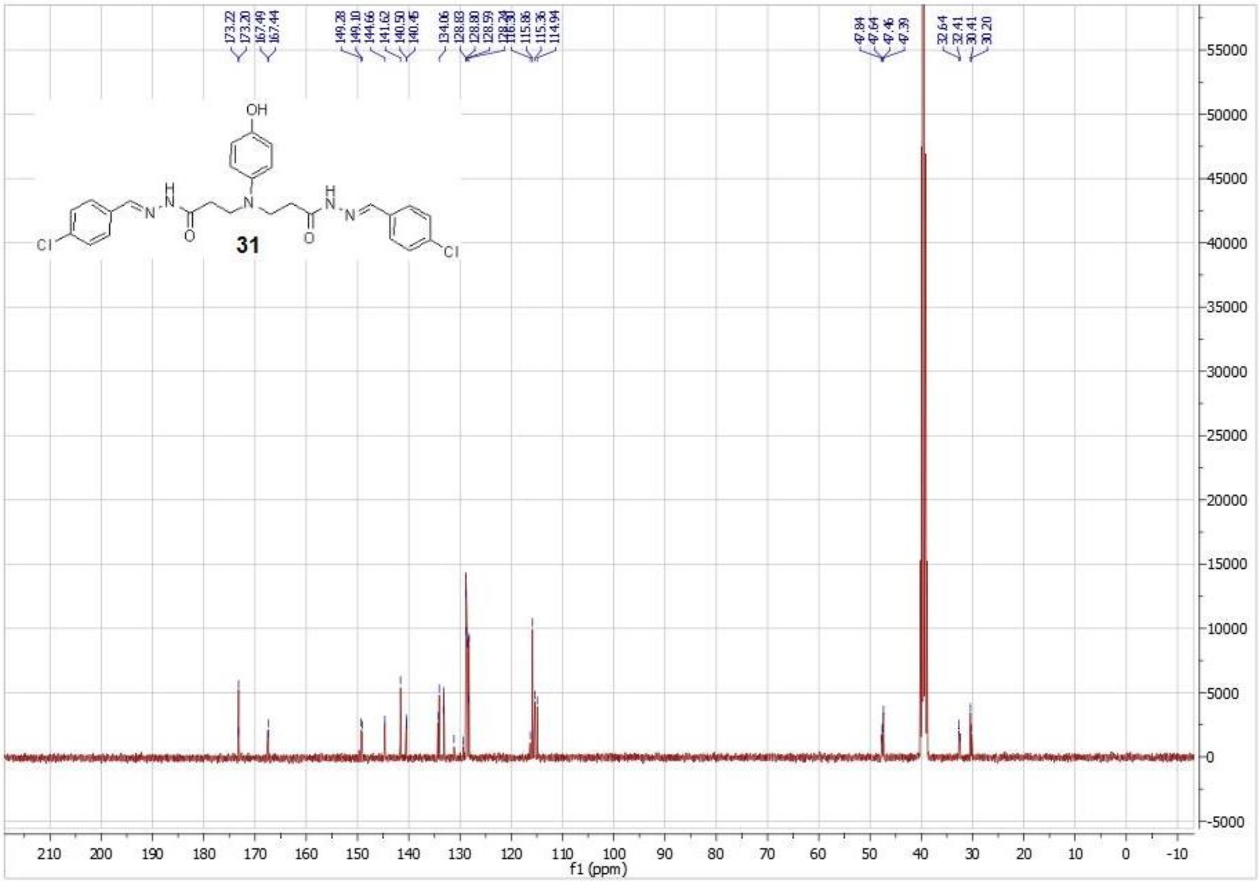

Figure S54. ^{13}C NMR spectrum of compound **31**

3,3'-((4-Hydroxyphenyl)azanediyl)bis(N'-(4-(dimethylamino)benzylidene)propanehydrazide) ***(32)***

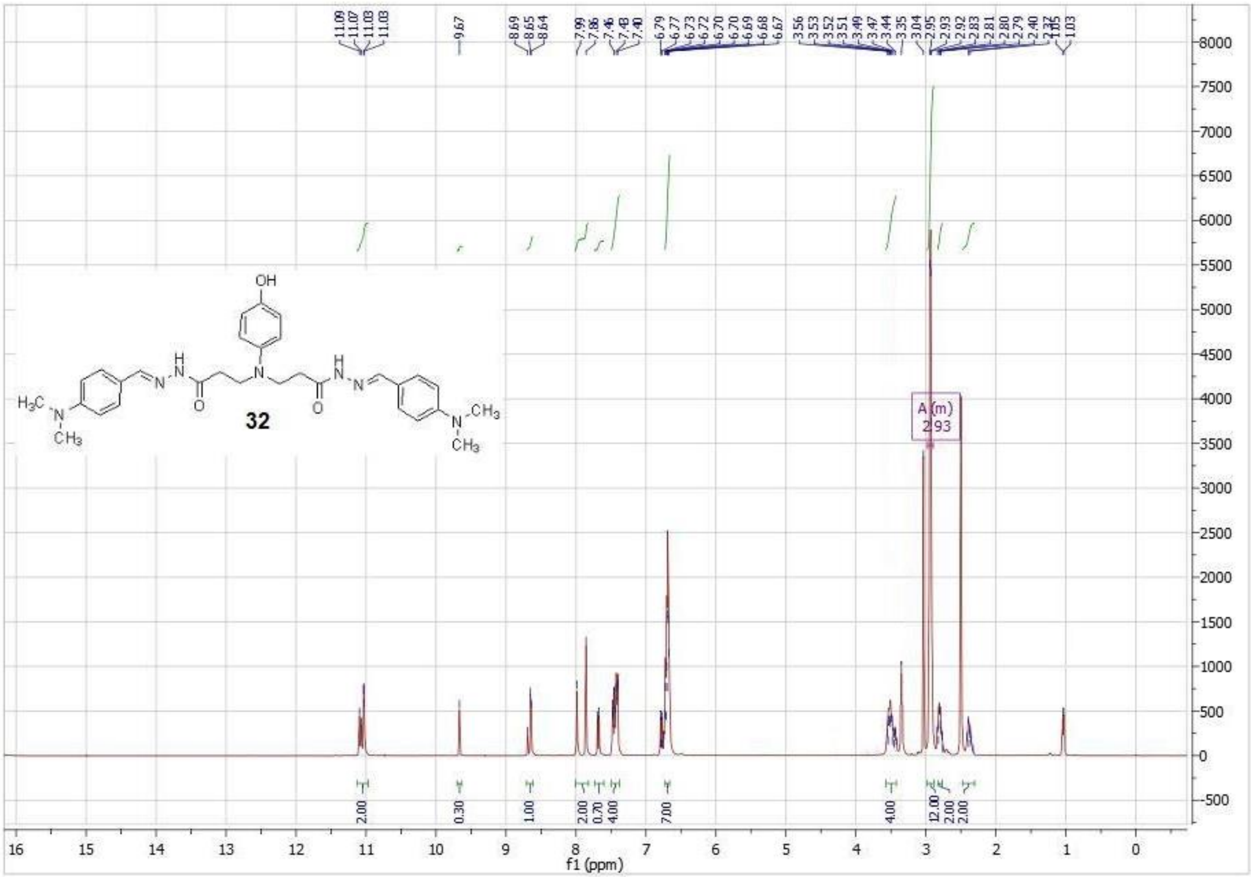

Figure S55. ^{1}H NMR spectrum of compound **32**

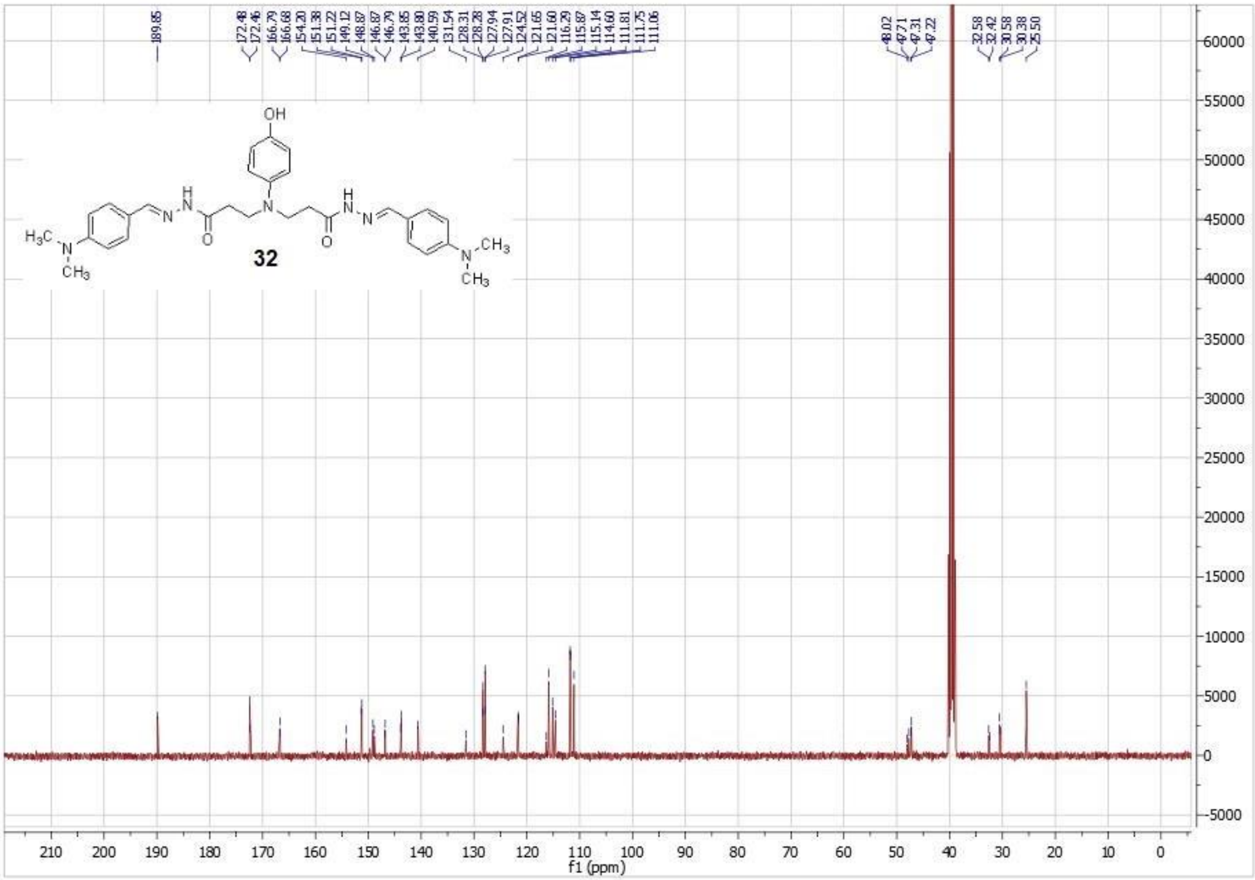

Figure S56. ^{13}C NMR spectrum of compound **32**

3,3'-((4-Hydroxyphenyl)azanediyl)bis(N'-(4-hydroxybenzylidene)propanehydrazide) ***(33)***

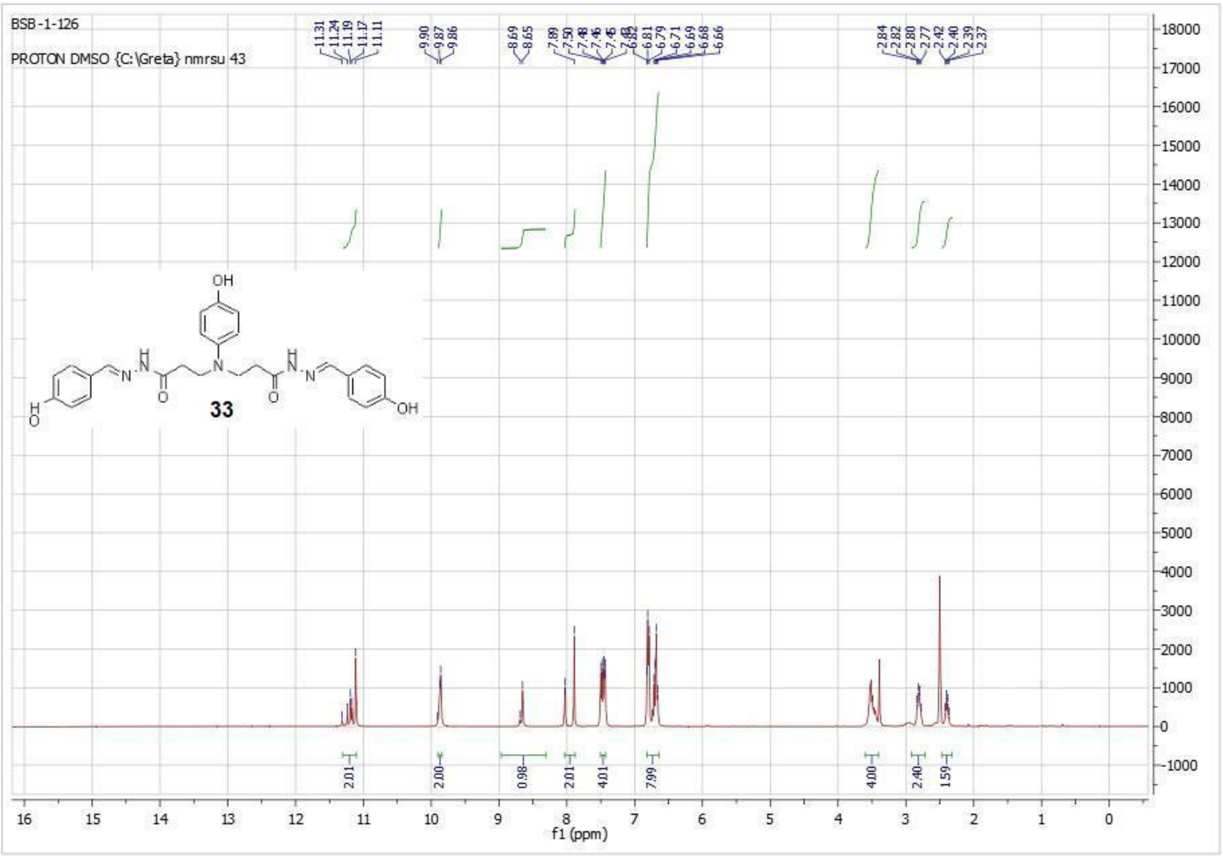

Figure S57. ^{1}H NMR spectrum of compound **33**

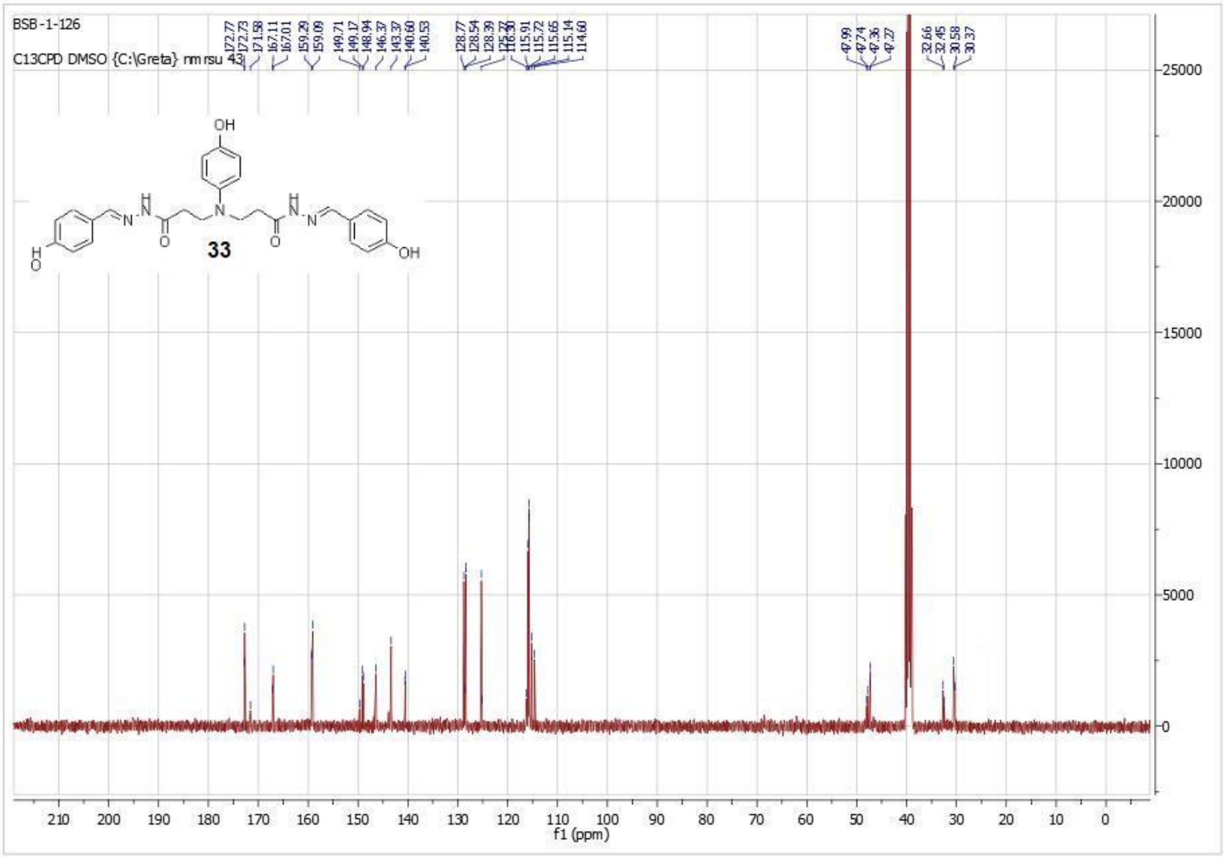

Figure S58. ^{13}C NMR spectrum of compound **33**

3,3'-((4-Hydroxyphenyl)azanediyl)bis(N'-(3,4,5-trimethoxybenzylidene)propanehydrazide) ***(34)***

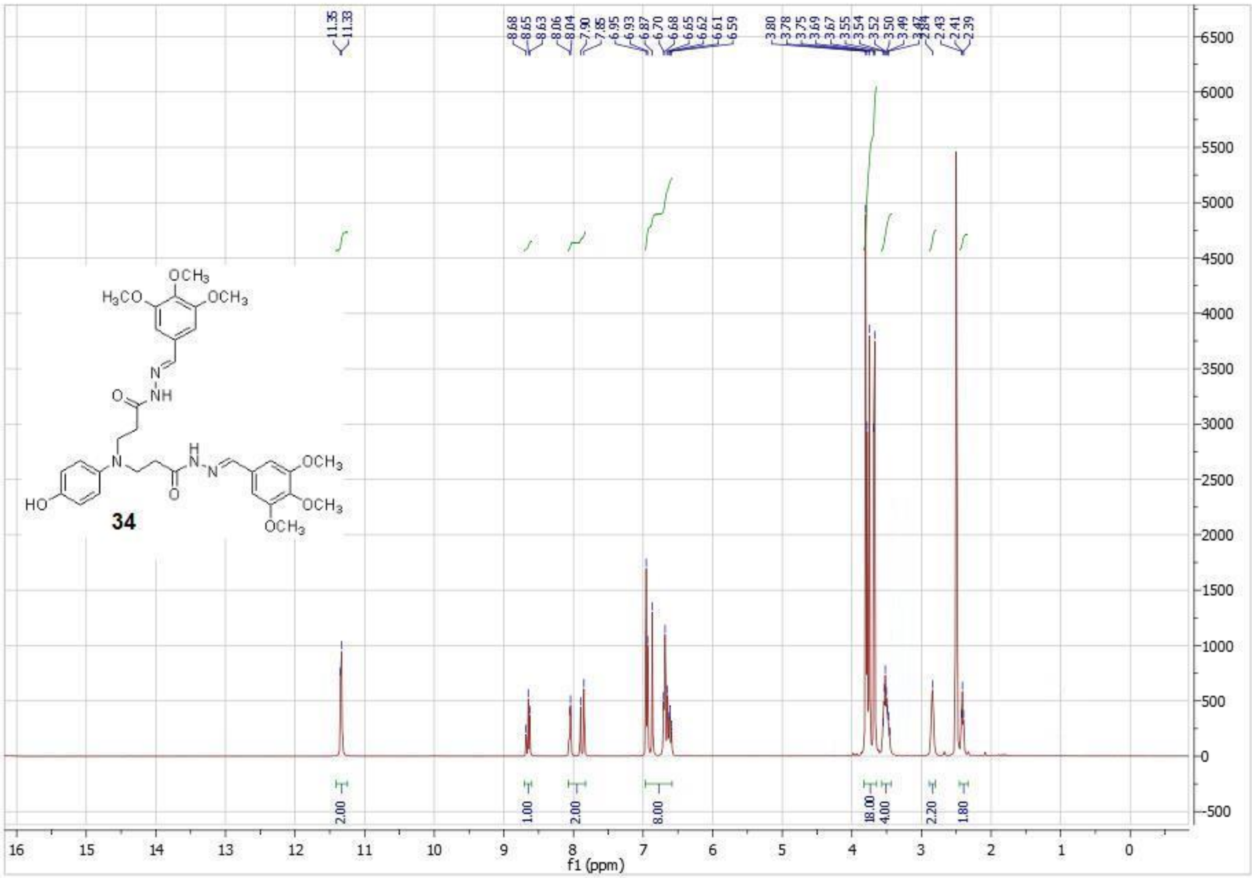

Figure S59. ^{1}H NMR spectrum of compound **34**

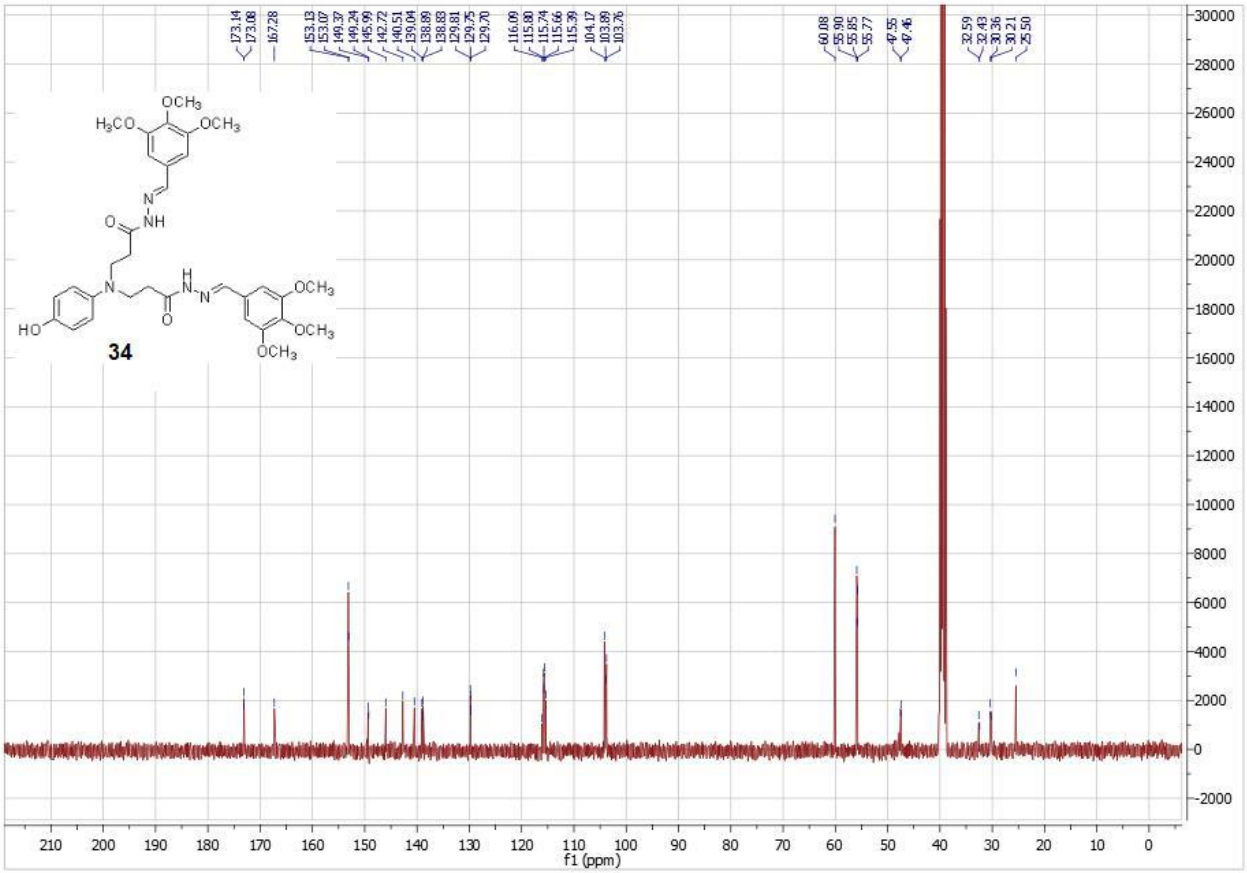

Figure S60. ^{13}C NMR spectrum of compound **34**

3,3'-((4-Hydroxyphenyl)azanediyl)bis(N'-(naphthalen-1-ylmethylene)propanehydrazide) ***(35)***

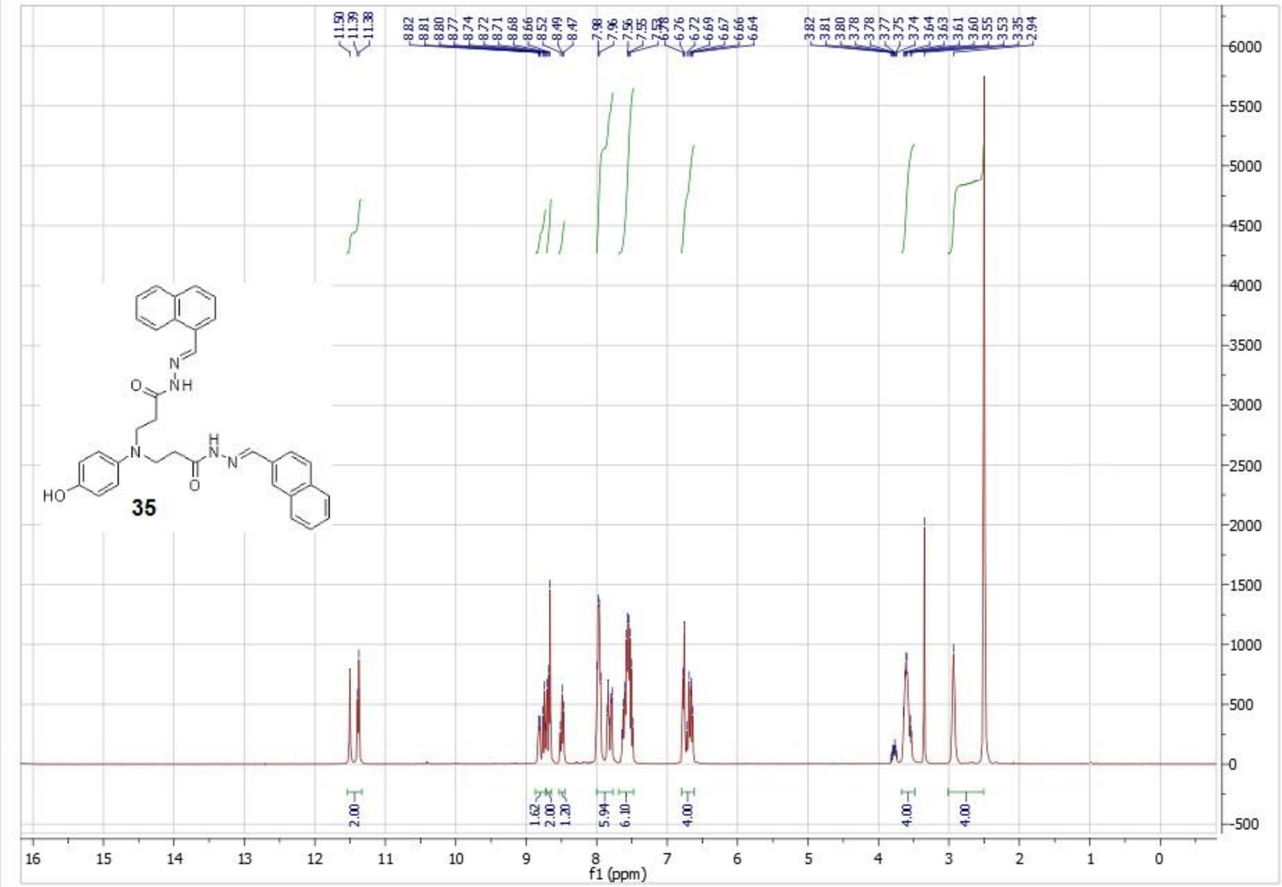

Figure S61. ^{1}H NMR spectrum of compound **35**

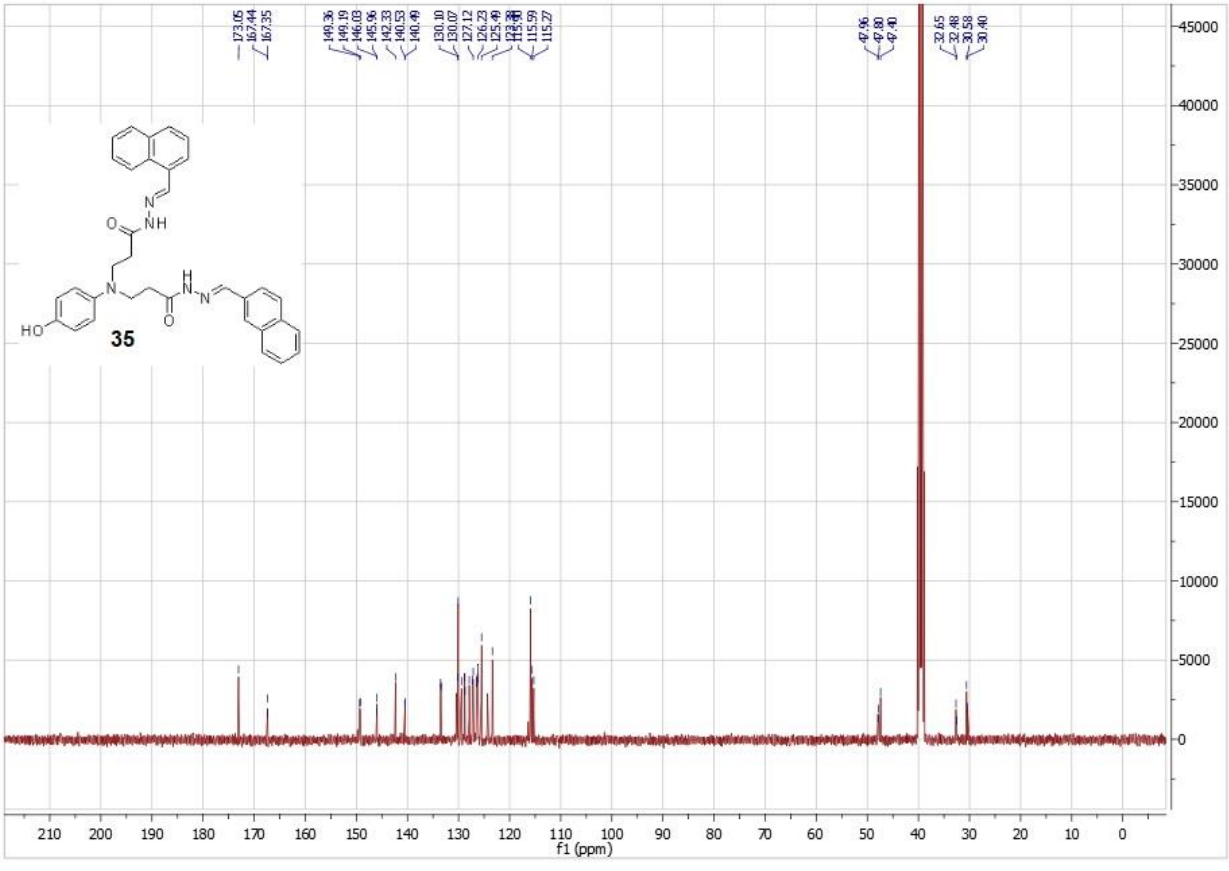

Figure S62. ^{13}C NMR spectrum of compound **35**

3,3'-((4-Hydroxyphenyl)azanediyl)bis(1-(3,5-dimethyl-1H-pyrazol-1-yl)propan-1-one) ***(36)***

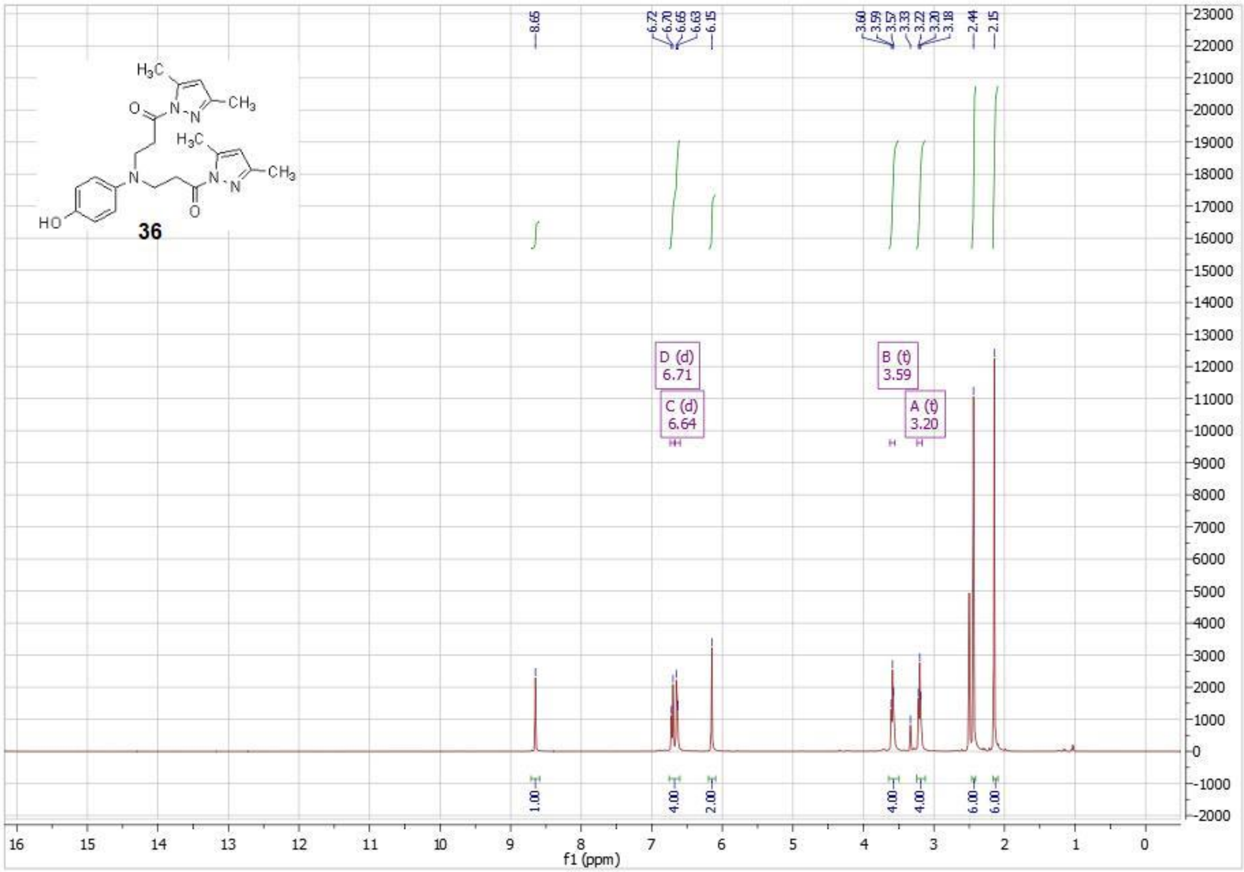

Figure S63. ^1^H NMR spectrum of compound **36**

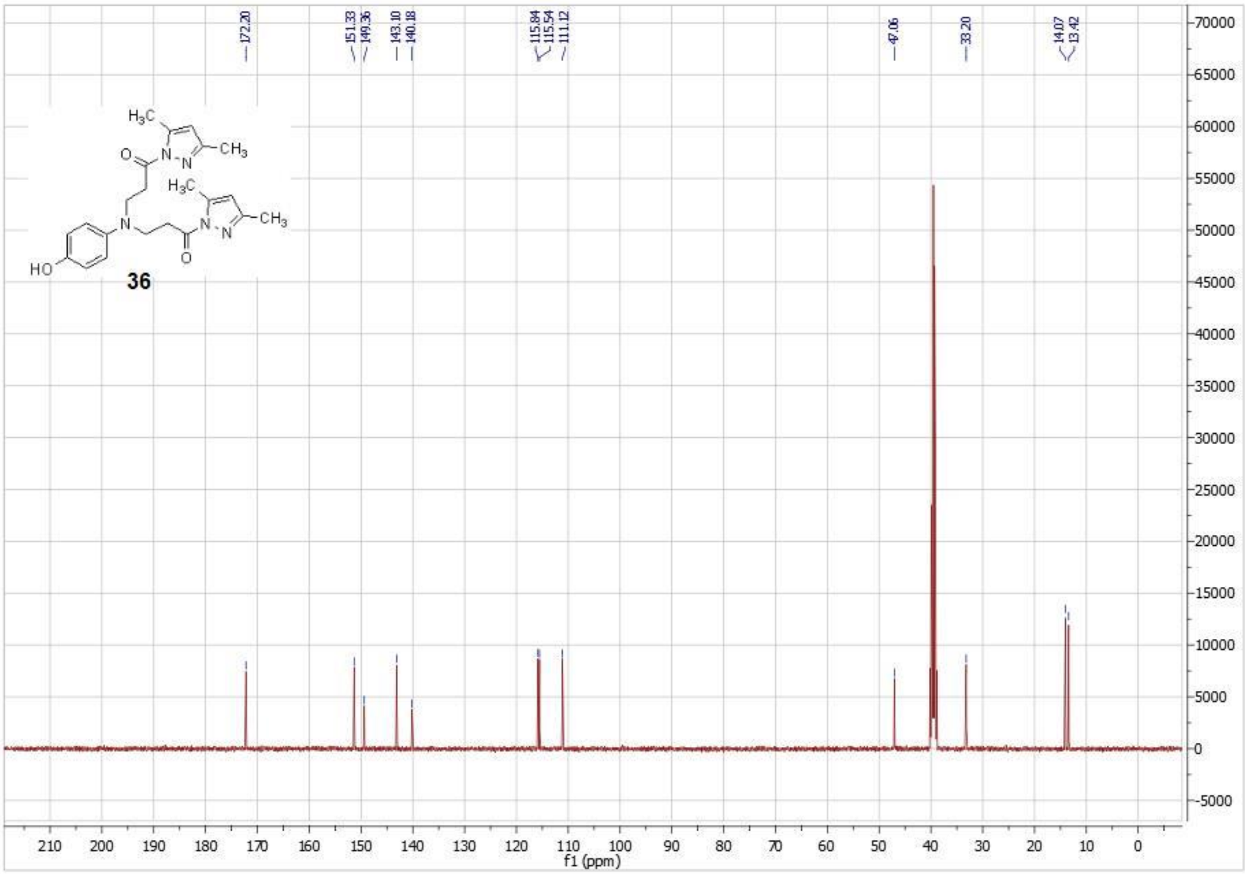

Figure S64. ^13^C NMR spectrum of compound **36**

3,3'-((4-Hydroxyphenyl)azanediyl)bis(N-(2,5-dimethyl-1H-pyrrol-1-yl)propanamide) ***(37)***

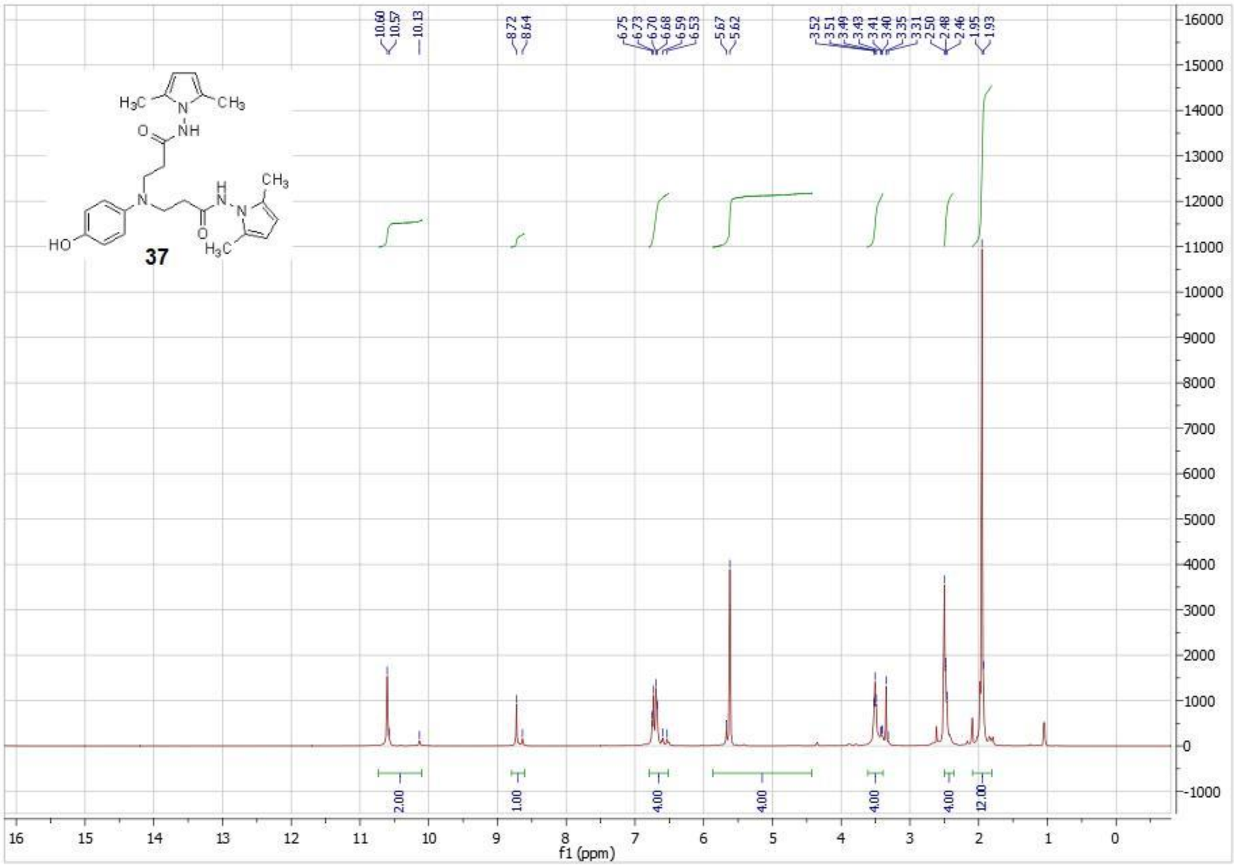

Figure S65. ^{1}H NMR spectrum of compound **37**

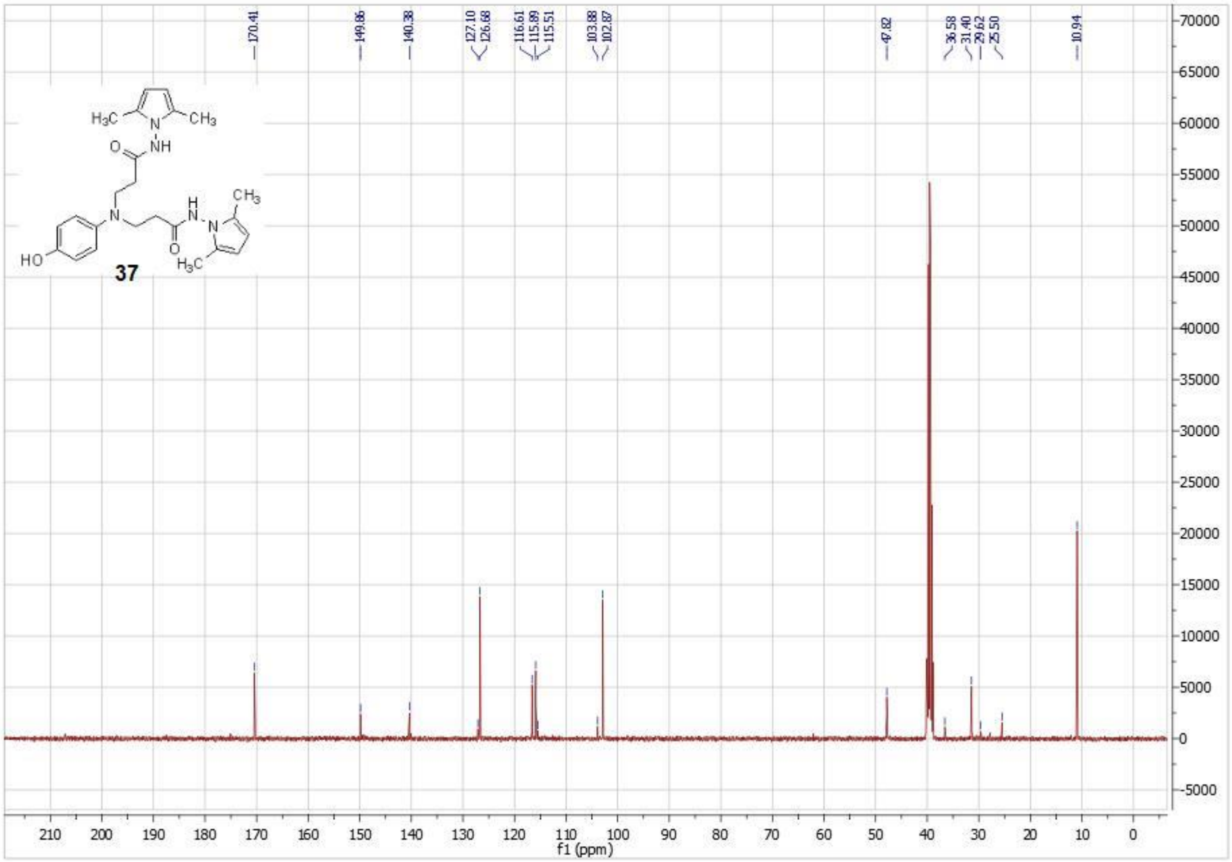

Figure S66. ^{13}C NMR spectrum of compound **37**

Acquisition Parameter

Source Type	ESI	Ion Polarity	Positive	Set Nebulizer	1.5 Bar
Focus	Not active	Set Capillary	4500 V	Set Dry Heater	180 °C
Scan Begin	40 m/z	Set End Plate Offset	-500 V	Set Dry Gas	8.0 l/min
Scan End	1800 m/z	Set Collision Cell RF	350.0 Vpp	Set Divert Valve	Waste

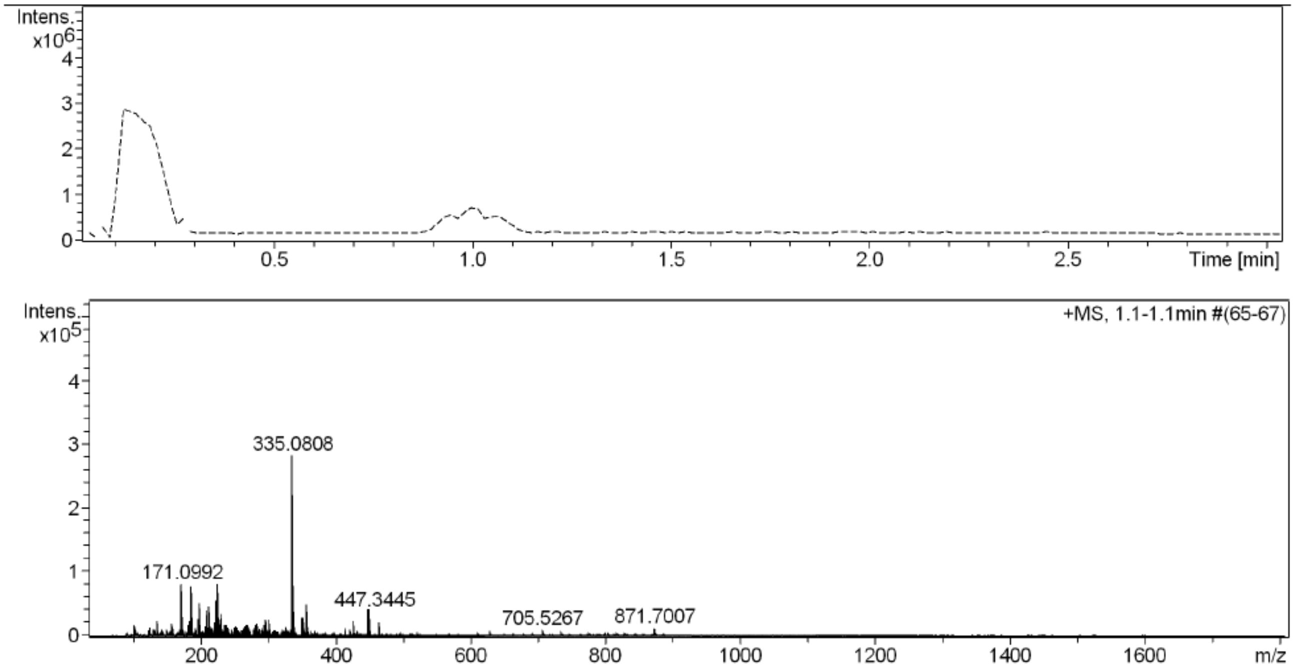

Figure S67: Mass spectrum of compound **15**
